# Large language model guided automated reaction pathway exploration

**DOI:** 10.1038/s42004-025-01630-y

**Published:** 2025-08-24

**Authors:** Ruzhao Chen, Yubang Liu, Zhe Chen, Yinwu Li, Fuyi Yang, Jiaxin Lin, Zhuofeng Ke

**Affiliations:** School of Materials Science and Engineering, PCFM Lab, the Key Laboratory of Low-carbon Chemistry & Energy Conservation of Guangdong Province, Sun Yat-sen University, Guangzhou, P. R. China

**Keywords:** Computational chemistry, Reaction mechanisms, Reaction mechanisms

## Abstract

Fast and efficient automated exploration of reaction pathways is essential for studying reaction mechanisms and advancing data-driven approaches for reaction development and catalyst design. Here, we present a new program (utilizing Python and Fortran), capable of conducting automated, fast, and efficient exploration of reaction pathways for potential energy surfaces (PES) studies. This program integrates quantum mechanics and rule-based methodologies, underpinned by a Large Language Model-assisted chemical logic. Both active-learning methods in transition states sampling and parallel multi-step reaction searches with efficient filtering help enhance efficiency and accelerate PES searching. Its effectiveness and versatility in automating searches are exemplified through case studies of multi-step reactions, including the organic cycloaddition reaction, asymmetric Mannich-type reaction, and organometallic Pt-catalyzed reaction. ARplorer’s capability to scale up for high-throughput screening significantly enhances its utility, positioning it as an efficient tool for data-driven reaction development and catalyst design.

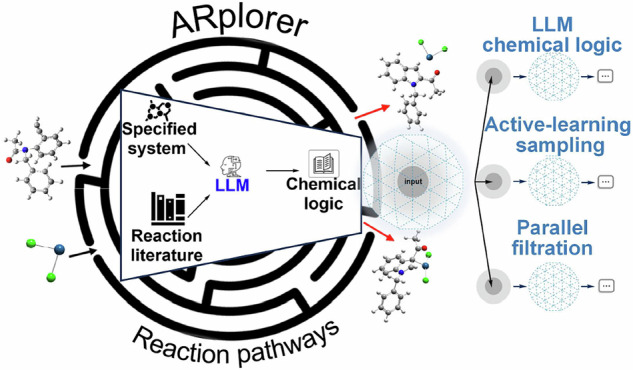

## Abstract (web summary)

Automated exploration of reaction pathways is crucial for advancing reaction mechanisms and catalyst design. Here, the authors introduce ARplorer, a Python and Fortran-based program that integrates quantum mechanics and rule-based methods, underpinned by a large language model-assisted chemical logic, enhancing efficiency in automating searches of multi-step reactions.

## Introduction

Chemical reactions are important in shaping the modern world, greatly impacting research, society, and various scientific fields. These reactions, fundamental to chemistry, involve the transformation of the reactants (RC) into products^1,2^. Predicting all chemical reactions presents a significant challenge due to the exponential growth in reaction pathways with each additional step^3^. Understanding these reactions hinges on the potential energy surface (PES), a complex function that depicts the energy states associated with atomic positions. In the PES, RC, and intermediates (IM) are identified as energy minima, whereas the transition state (TS) represents a crucial first-order saddle point connecting these minima. These three states—RC, IM, and TS—are essential for analyzing chemical reactions. Although RCs and IMs can often be detected experimentally, TSs are more elusive, typically revealed through theoretical simulations. Investigating TSs is fundamental to understanding reaction kinetics and assessing the feasibility of reaction pathways^4,5^, thereby serving as a key component in chemical reaction and PES analysis of reaction pathways.

Expanding upon this foundation, the importance of theoretical calculations in predicting chemical reactions becomes evident, highlighted by various methods used to automate the exploration of the PES for reaction pathways^6^. Conventional approaches such as quantum mechanics (QM) and molecular dynamics (MD) simulations are traditionally used to investigate multistep reaction pathways and various reaction mechanisms. However, these approaches often generate impractical pathways due to unfiltered search strategies and are notorious for their substantial time requirements^7^. Given the desire for efficiency and rapidity in theoretical reaction prediction, driven by the growing attention to data-driven reaction development and catalyst design, there is a strong demand for a fast, efficient, and automated approach to exploring reaction pathways^8^.

A foundational technique in exploring chemical reactions is global reaction route mapping, which utilizes a fully automated search of the PES through methods like anharmonic downward distortion following and artificial force-induced reaction to identify potential IMs and TSs^9^. Additionally, ab initio molecular dynamics represents a significant QM simulation method, which has shown promise in certain systems^10^. Besides, methods combined molecular dynamic and coordinate driving method called automated design of chemical reaction (ADCR) was developed^11,12^, which can further consider the influence of solvent molecules on the reaction mechanism while maintaining a comprehensive search of reaction. In response to the limitations of unfiltered searches, rule-based systems were introduced, applying predetermined biases to discard unlikely pathways, refine the search, and enhance the search process. These rule-based approaches involve a guided search of the PES based on encoded rules. Zimmerman^13^ and Aspuru-Guzik^14^ proposed using representative reaction patterns as rules to guide the PES search, and some software such as Kinbot^15^, AutoMech^16^ and RMG/T3/ARC/AutoTST^17^, which rely on a collection of reaction templates curated by the developers, contribute a lot to this area as well. These rule-based approaches generally mitigate some limitations of PES search and TS identification techniques.

Another intriguing approach involved encoding RC as simplified molecular input line entry systems (SMILES) and matching potential paths with existing reaction templates^18^. Reiher^19^ applied local quantum parameters, such as atom charges, to guide the exploration of the PES. Emerging from the rule-based domain, machine learning has been integrated to develop or enhance reaction pathways search strategies^6^. Coley has successfully employed machine-learning models to search reaction routes and discover new active compounds^20^. However, the thermodynamic and kinetic information based on PES is highly desired for reaction development and catalyst design, in comparison to the simple reaction routes analysis.

Recently, LLMs have played an important role in chemistry research. The study by Jablonka et al. indicated that fine-tuned LLMs can excel in chemical and materials science tasks, including predicting molecular properties and chemical reaction yields, especially with limited data^21^. Su et al. discussed how LLMs enhance automation and machine learning in catalysis, streamlining information extraction, optimizing experiments iteratively, and generating new insights^22^. The study by White demonstrated that large language models (LLMs) can understand and apply chemical knowledge, when prompted, with accuracy significantly improved through prompt engineering strategies^23^. Wu introduced Prompt-MolOpt, a tool leveraging LLMs for advanced multi-property molecular optimization, which shows remarkable performance in optimizing molecules for specific properties while preserving pharmacophores^24^. These studies show LLMs as integral to the future of chemical research and catalyst development, emphasizing their interactive and collaborative potential with human researchers and their ability to summarize chemical logic or reactions.

Machine learning potential energies, especially neural network potential energies (NNPs), have developed significantly over the last two decades and are now widely used for large-scale atomic simulations in chemistry, physics, and materials sciences^25–29^. By mapping from atomic structure to potential energy, NNPs successfully combines the flexibility and efficiency of machine learning, significantly improving computational efficiency compared to traditional potential energy surface methods, such as density functional theory (DFT) or molecular mechanics force fields, while maintaining high accuracy.After development, NNPs have shown great versatility and flexibility in describing a variety of chemical bonds and interatomic interactions^30–32^. Many outstanding works, such as LASP^33–35^ and MLatom^36,37^ have been widely applied in the field of computational chemistry, physics, and materials science for large-scale atomic simulations. Despite the clear advantages of NNPs in simulation accuracy and computational efficiency, they still face several challenges and disadvantages. The performance of NNPs is largely limited by the quality and diversity of training data, there is a risk of overfitting, and it may lack accuracy for new or chemical environments not covered by training data^38,39^. In addition, the training process of NNPs requires significant computational resources and is challenging in terms of interpretability, as they are usually treated as “black box” models with less than transparent decision-making processes^40^.

**Fig. 1:**
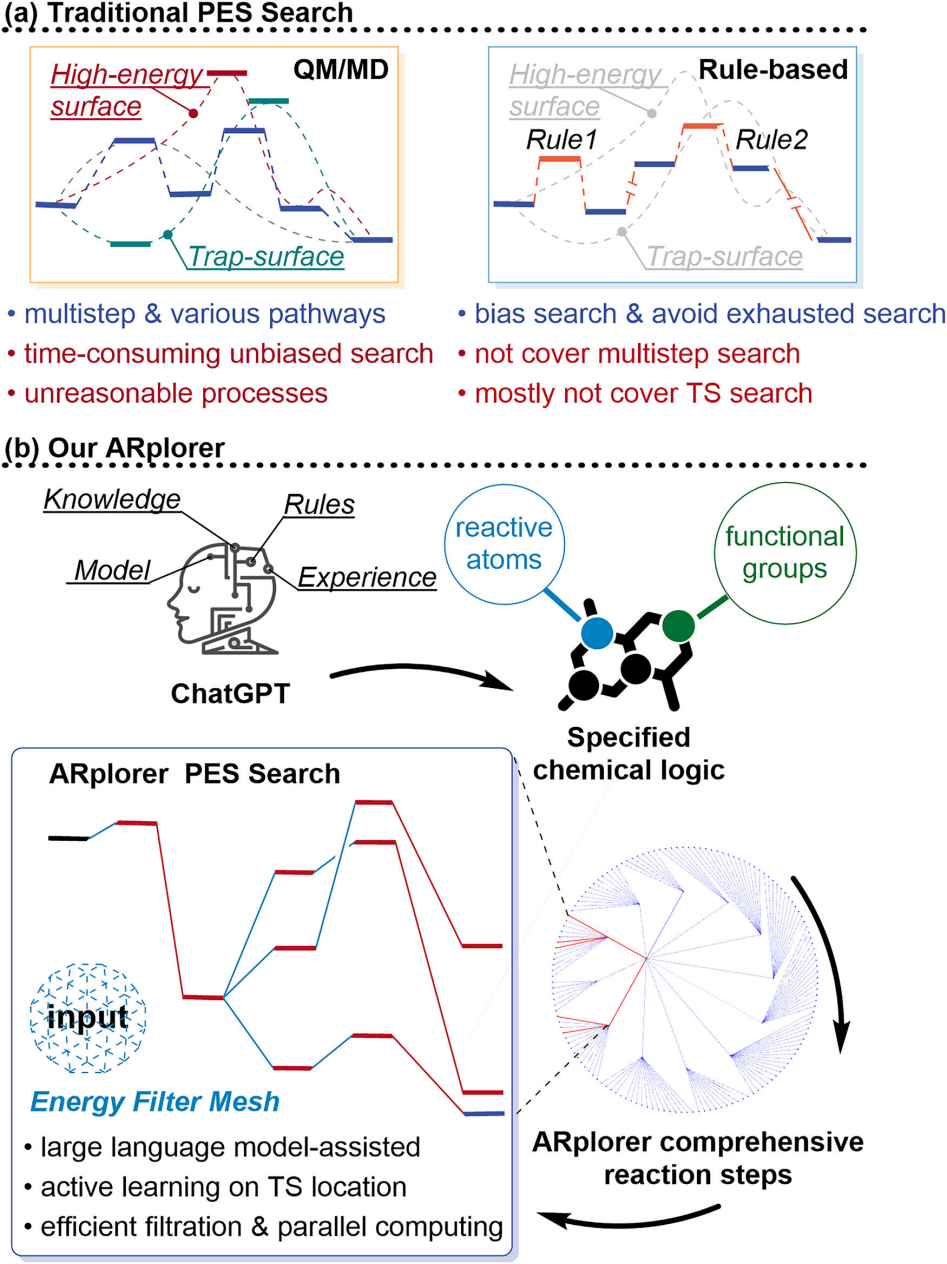
BackGround and ARploer. **a** Limitation of traditional approaches. **b** Advancement of ARplorer.

Despite the development of these methods, there are still limitations to be overcome, especially when dealing with complicated organic and organometallic systems with complex reactants and multistep reaction pathways. This limitation primarily arises from the absence of chemical logic implementation based on existing literature and the need for system-specific modifications, as depicted in Fig. 1a. There remains an opportunity to enhance the automated exploration of reaction pathway programs for PES studies by merging universally applicable chemical logic derived from the literature with system-specific chemical logic based on functional groups.

To overcome PES exploration challenges, we developed ARplorer, an automated computational program utilizing Python and Fortran, which integrates QM methods with rule-based approaches (Fig. 1b). ARplorer combines a biased auto-search mechanism with LLM-guided chemical logic, substantially increasing computational efficiency in identifying multistep reaction pathways and TS. It performs rule-guided PES searches augmented by case-specific chemical logic to filter unconventional paths and adaptively explore relevant reaction pathways. Furthermore, ARplorer employs an energy filter-assisted parallel computing framework and an active learning method for transition-state localization, effectively minimizing unnecessary computations. By integrating precise QM techniques with rule-based practicality, ARplorer achieves accurate and efficient identification of reaction pathways. Our results demonstrate ARplorer’s significant improvements in computational efficiency, practicality, and accuracy over conventional approaches.

## Results

### Program overview

**Fig. 2:**
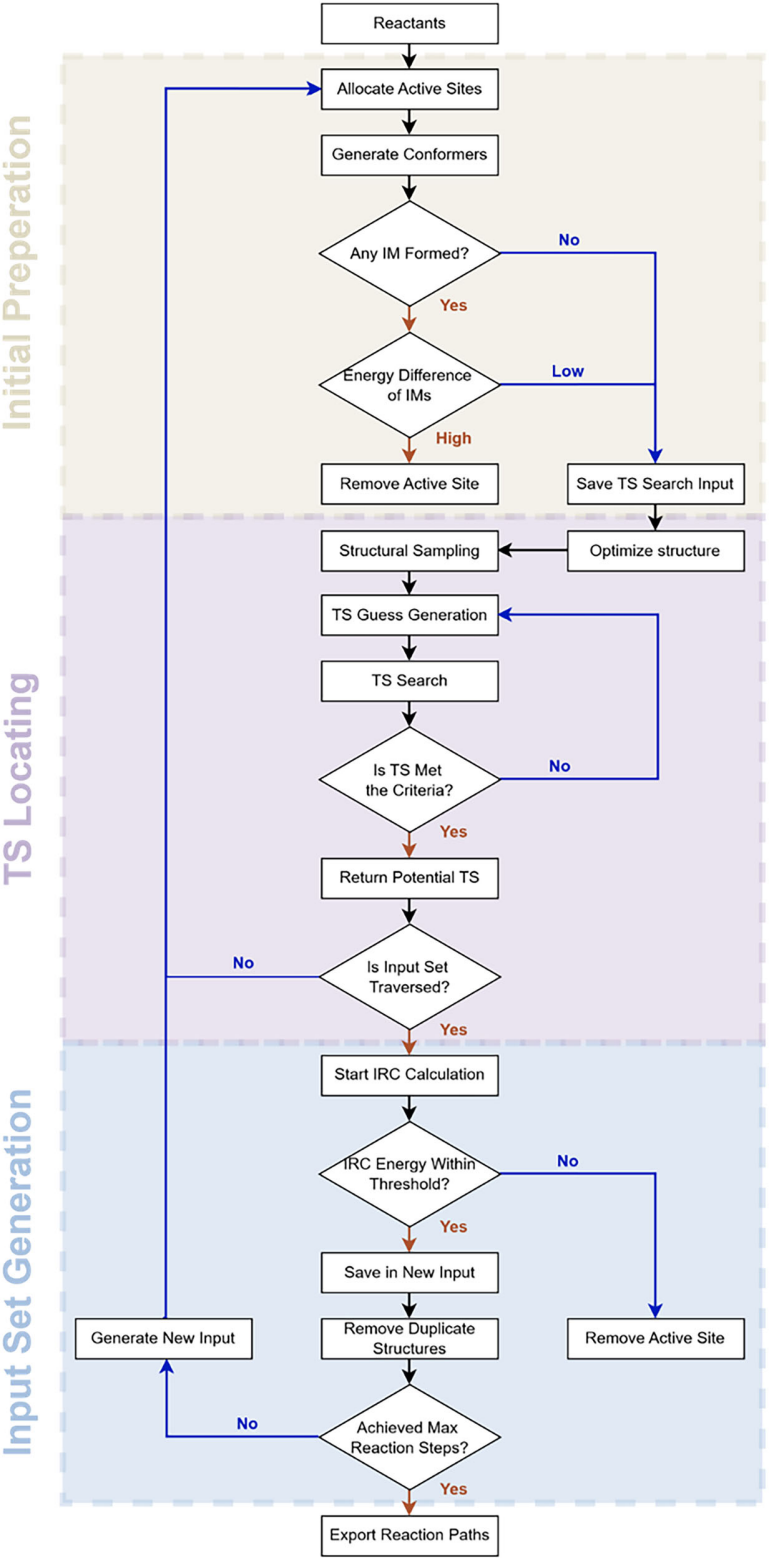
Brief workflow of ARplorer. The workflow proceeds through three main stages: Initial Preparation, TS Locating, and Input Set Generation, as indicated by the section labels.

The program overview of ARplorer is shown in Fig. 2. The ARplorer operates on a recursive algorithm, with each iteration involving the steps below: (1) Identify active sites and potential bond-breaking locations to set up multiple input molecular structures and perform analysis of IM for analyzing reaction pathways. (2) Optimize the molecular structure through iterative TS searches, employing a blend of active-learning sampling and potential energy assessments to hone in on potential intermediates. (3) Perform Intrinsic Reaction Coordinate (IRC) analysis to derive new reaction pathways from the optimized structures, eliminate duplicates, and finalize the structure post-IRC for subsequent input. For efficient reaction pathway exploration, in this work, Gaussian 09^41^ (of course, other programs’ algorithms can also be used) is combined with GFN2-xTB^42^. The ARplorer program uses GFN2-xTB to generate the potential energy surface and uses Gaussian 09’s algorithm to search the potential energy surface generated by GFN2-xTB. The flexibility and practicability of ARplorer are evident in the ability to switch between different levels of computational methods based on the requirements of the task at hand. For instance, GFN2-xTB can be employed for quick, large-scale screening, while DFT can be reserved for more detailed and precise calculations when necessary. It is important to note that the program’s workflow is independent of the choice of software package, and only minor adjustments are required to make it compatible with a specified software package.

### LLM-guided chemical logic for general and specific reactions

**Fig. 3:**
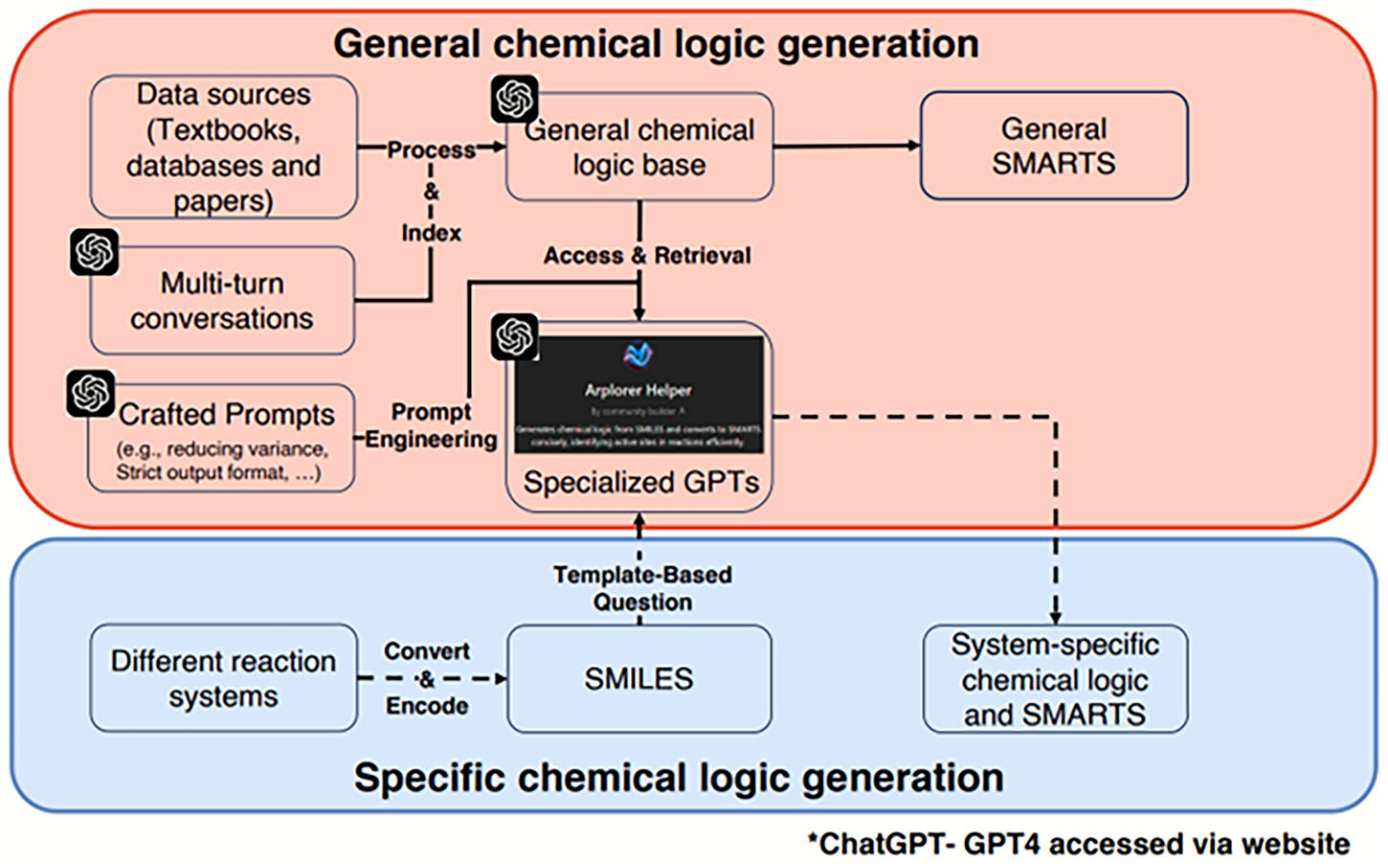
Large language model-assisted general and modifiable chemical logics. General chemical logic is constructed from diverse data sources, multi-turn conversations, and engineered prompts, forming a general logic base and generalized SMARTS patterns. Specialized GPT models retrieve and apply this logic to user queries. In the specific chemical logic generation layer, different reaction systems are encoded as SMILES, allowing for template-based questions and generation of system-specific chemical logic and SMARTS.

The chemical logic in ARplorer is built from two parts: pre-generated general chemical logic from literature and system-specific chemical logic from specialized LLMs, as depicted in Fig. 3. Initially, General chemical logic generation begins by processing and indexing prescreened data sources, such as books, databases and research articles, to form a general chemical knowledge base. Subsequently, this knowledge base is refined to create general SMARTS patterns. It is processed with prompts generated through prompt engineering to reduce output variance and implement strict formatting guidelines to assist specialized LLMs in generating template-based questions. These prompts and data accessed and retrieved from the general knowledge base enable the LLM models to generate targeted chemical logic and SMARTS patterns for specific systems. To encode system-specific rules, we convert reaction systems into SMILES format. Then, this conversion allows us to generate system-specific chemical logics and SMARTS patterns using specialized LLMs. Once the chemical logic library is curated, the ARplorer can explore the full PES automatically. In the current ARplorer workflow, users conduct fully deterministic reaction space exploration through combinatorially generated pathways that are strictly assessed by quantum mechanical (QM) calculations. The LLM serves exclusively as a literature mining tool during the initial knowledge curation phase, with no involvement in energy evaluation or pathway ranking. Reaction plausibility and reaction kinetics are evaluated exclusively via first-principles computations. This guarantees that ARplorer performs autonomous reaction path discovery and intrinsic activity evaluation while adhering strictly to rigorous quantum chemical foundations; a detailed workflow illustrating how ARplorer operates is provided in Fig. S15.

For a given reaction system, Pybel, a Python module, was utilized to compile a list of active atom pairs (including potential bond-breaking) from chemical logic in SMARTS format to align the system with the encoded rules. This list, obtained after filtering out repeated pairs, concretizes abstract chemical logic and aids in guiding the PES search. If elementary reactions are expected, in theory, we can map out any complex reaction pathway, regardless of its intricacy. While the PES search, guided by established chemical rules, may overlook unknown elementary reaction steps, it is expandable and contributes to the enhancement of searching for a deeper understanding of the PES in complex systems. This adaptability is particularly valuable as such complexity often surpasses human capability due to cognitive limitations and potential oversights. Although LLMs will provide some faulty answers inevitably, ARplorer’s reaction pathways search based on first principles can more objectively judge whether the resulting reaction pathways caused by this logic are reasonable and feasible, with energy threshold, from the perspective of first-principle PESs. To test if there is obvious stochasticity in ARplorer, a replicate run of case 2 (see the section of Result and Disscusion) and tests of change of energy threshold have been performed. The results of the stochasticity test was shown in the section of Stochasticity test of ARplorer in Supporting Information.

### Parallel computing of reaction pathways with highly efficient energy filtration

Parallel computing is also one of the important features in this work because when the guidance of chemical logic is combined with TS-location and multi-step search, the consumption of time inevitably increases. In order to achieve highly efficient filtration, a customized function **U** [Eq. (1)] from Duarte’s work would be introduced^43^. This function relaxed a given structure to a stable structure with low potential energy, i.e., optimizes and minimizes the total energy of the structure. The detailed application of function U in this work is shown in Fig. S8.

1 $$U={\sum}_{i,j\,\in {bonds}}{a\left({r}_{{ij}}-{r}_{{{ij}}_{{bond}}}\right)}^{2}+{\sum}_{i < j}\frac{b}{{r}_{{ij}}^{n}}$$

**Fig. 4:**
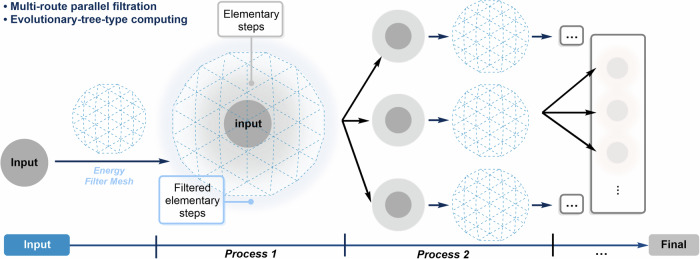
Parallel framework of the program. Input structures are screened using an energy filter mesh to generate a set of filtered elementary steps. These steps are then processed in parallel through sequential stages, with each stage applying further filtration and branching.

The parallel computing in this program is realized through the multiprocessing module and concurrent module built-in python3, as depicted in Fig. 4. For the input reaction system, ARplorer generates the corresponding active site pairs through reaction logic. The program then performs further processing of the active site pairs in parallel, including conformational search, intermediate analysis, TS search, and IRC analysis. Through energy filtering, a number of new reaction paths and corresponding intermediate sets were obtained (Fig. 4. Process 1). The intermediate set is used as a new input structure set to carry out the next step of pathways search in parallel (Fig. 4. Process 2), that is, similar to the first step, until the reaction system reaches the specified number of reaction steps. The extent of parallel computing is regulated by the “npool” and “npool_tes” parameters, which are adjusted based on the server’s core count and memory capacity.

### Active-learning approach for sampling TS locations

**Fig. 5:**
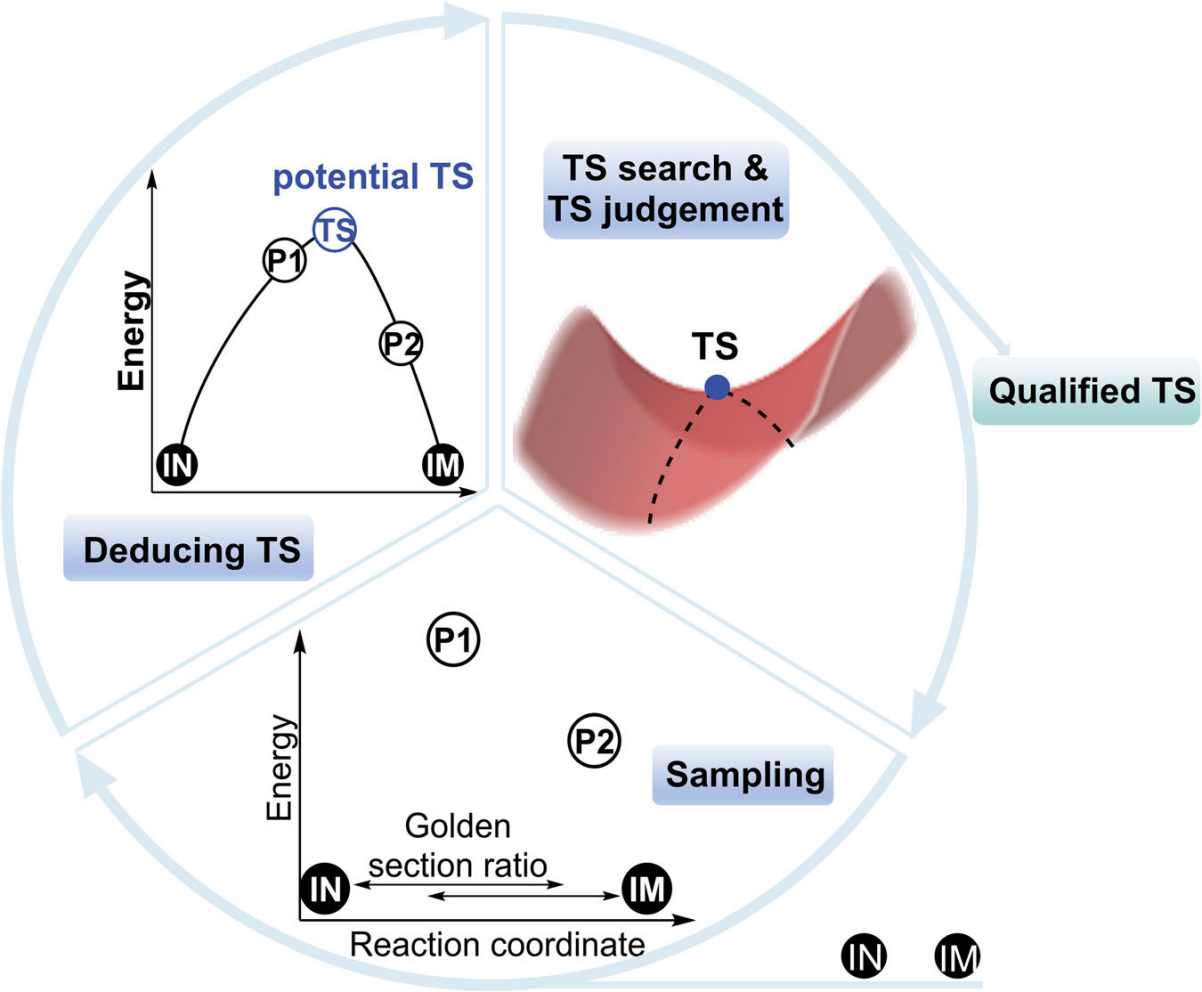
Concept map of active-learning methods in TS sampling. The approach involves iterative sampling between initial (IN) and intermediate (IM) structures, utilizing the golden section ratio to generate probe points and fitting energy profiles to estimate potential TS locations.

The TS location holds significant importance in the program due to its role in evaluating the kinetic feasibility of reaction pathways. We have incorporated a streamlined and effective TS location approach termed “active learning for sampling.” This strategy is predicated on learning from sampled data points to update potential TS locations. The approach entails an iterative algorithm, illustrated in Fig. 5, and a detailed explanation in Fig. S10. For a given elementary step, an initial structure (IN) is provided, and the subsequent IM is identified using the custom function U, bypassing the direct TS location. The brief workflow of the active-learning approach is as follows: (1). For a given IN (begin from IM is similar), probe structures were constructed in the first golden ratio point (the one is close to IN) between IN and IM. Then, the probe structures were optimized with the active site constrained. (2). Three consecutive probe points, including the one with the highest energy, would be fitted into a quadratic function curve, of which the extreme point would be guessed as the potential saddle point. (3). If a qualified TS was not obtained, this failed TS would be served as new probe point and the program return to step 2 until a qualified TS was found or reached the maximum step.

The program analyses energy trends among the probe points to predict potential TS locations. If the potential TS is deemed unjustifiable, this was considered unrealistic and would become a new probe point after optimization. This iterative cycle persists until a potential TS is validated or a set maximum number of iterations is reached. To validate a potential TS, a two-step method is employed. The first step involved the weight coefficient of the vibration of the active atom pair in the eigenvector corresponding to the negative eigenvalue of the last step of the TS location algorithm. If this coefficient ranks within the top 10 of all coefficients in the last phase, the first criterion is met. The subsequent step initiated only upon meeting the first criterion, involves checking for a unique virtual frequency in frequency analysis. To test the efficiency and stability of this method, a set of 59 reactions that from RMG database^17^ was chosen as the benchmark set for the TS searching. The detail of this benchmark could be found in SI. The total success ratio is up tp 90%, which means active learning sampling on TS location is stable and efficient.

### Variety

**Fig. 6:**
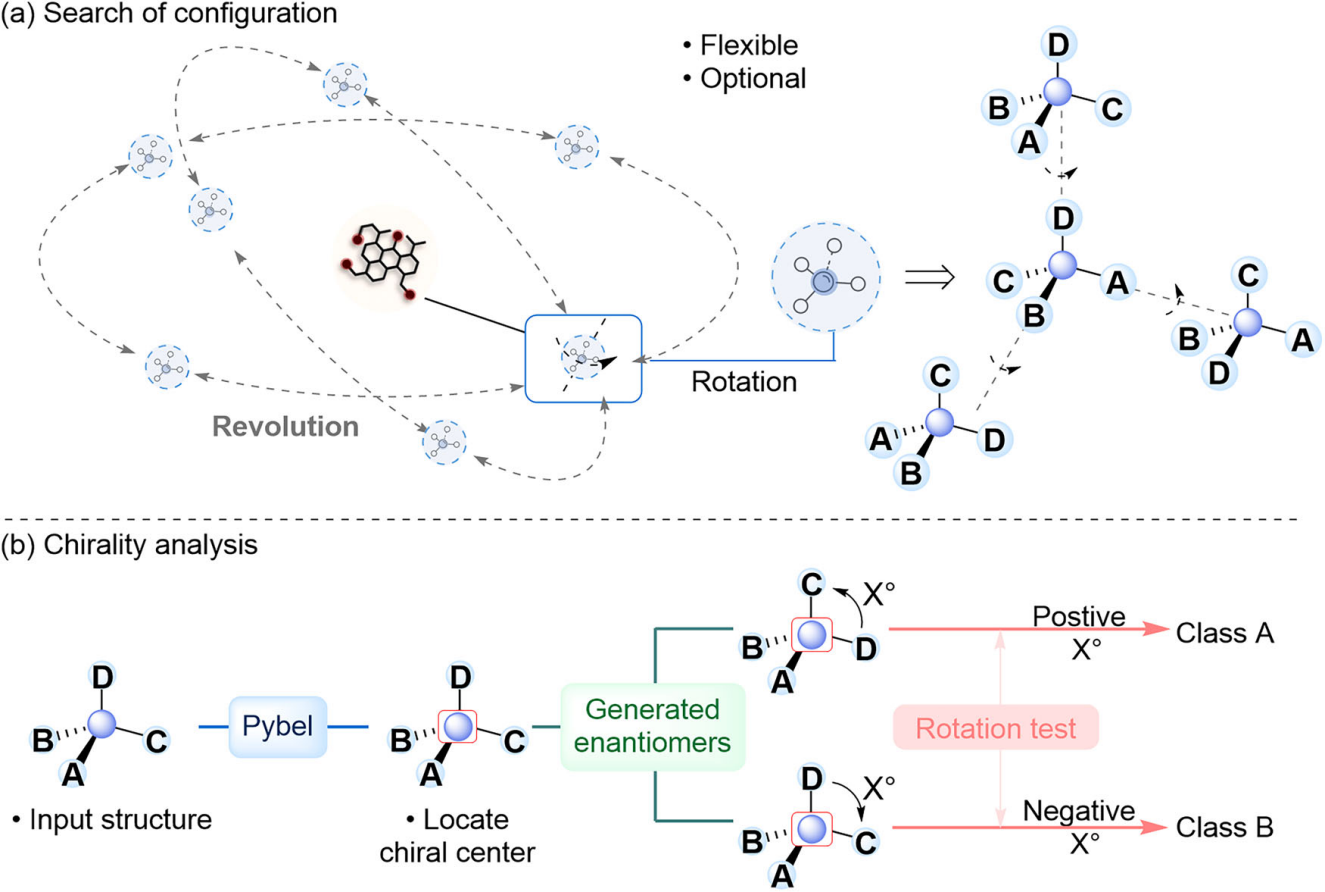
Conformation and chirality searching. **a** Enriched configuration through spin coupled with revolution. **b** Difference between enantiomers when the rotation test was performed.

The number of conformations of the whole reaction system is very large, and most of the conformations do not affect the direction of the reaction. Therefore, ARplorer focuses only on conformations associated with the active sites. This was accomplished through structure rotation and the use of function U. When a specific structure and active atom pair were provided, multiple configurations were created through rotation, as illustrated in Fig. 6a. The portion with active site B rotated while simultaneously revolving around the part with active site A. To avoid overlaps between parts A and B, function U was utilized to relax the structure following rotation. Subsequently, an initial calculation was conducted. The conformations are extracted at a certain frequency, and the ones with high energy are screened out by the energy threshold and the root-mean-square deviation of distances between atoms was employed to eliminate redundant structures.

### Chirality

The program is equipped to handle asymmetric chemical reactions involving chiral atoms. This feature could be activated or deactivated by adjusting the “chiral” parameter, which could be set to True or False. It consisted of three components: The first part involved the recognition and collection of chiral carbon atoms using Pybel; The second part aimed to generate enantiomers through rotation and the use of function U; The third step classifies the elementary step accurately by rotating the structure to maintain the same atomic connections but considering the different spatial relationship of the chiral atom and its connected atoms, illustrated in Fig. 6b rotation test. This process of classification results in varied outcomes.

## Applications and discussion

For this program to be applicable for fast and efficient automated exploration of reaction pathways, we further demonstrated it in representative multi-step reactions. These include the organic cycloaddition reaction, asymmetric Mannich-type reaction, and organometallic Pt-catalyzed reaction.

### Case 1: Lewis acid-promoted cycloaddition reaction

**Fig. 7:**
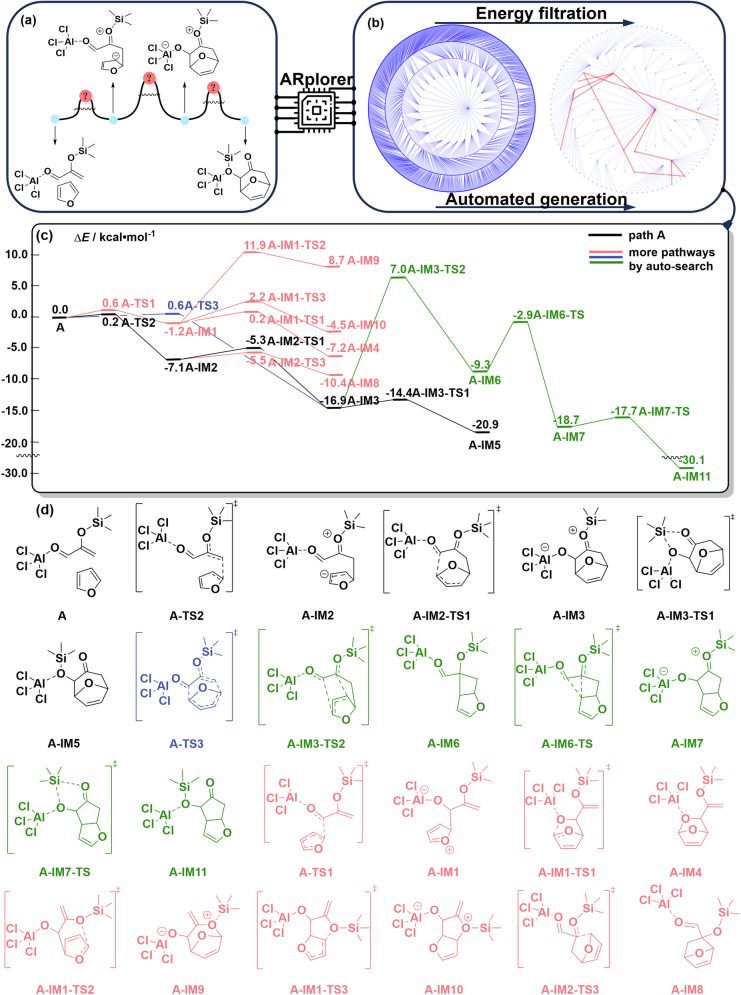
Important reaction pathways and structures of the PES disclosed by ARplored in Case 1. **a** Previously proposed reaction mechanism. **b** Overall network of exploration of reaction pathways by ARplorer. **c** The important reaction pathways generated by ARplorer, the black path (the path A) is the most plausible. **d** Selected structures.

The program was utilized to investigate the reaction pathways of the reported Lewies-acid-promoted [4 + 3] cycloaddition of 2-(trimethyl silyloxy)acrolein with furan^44^. The previously proposed reaction pathway resulted from manual DFT calculations (path A) as shown in Fig. 7a.

ARplorer exhibited excellent performance in setup and systematically explored all potential reaction pathways guided by the LLM-based chemical logic. The comprehensive exploration of the reaction pathways and the determination of the reaction mechanism required only 1.5 days of computational time on a 24-core (Xeon GOLD 6342) setup. The automated exploration network of the whole reaction pathways is shown in Fig. 7b, and the detail of postprocessing of a total of 874 reaction steps is shown in Fig. S9. ARplorer not only successfully identified a reaction pathway (path A) that aligns with the previously proposed one but also discovered additional plausible reaction pathways (filtered by 0.05 Hartree, as shown in Fig. 7c, enhancing our understanding of the investigated AlCl_3_-promoted cycloaddition reaction. The selected chemical structures for key intermediates and TS for the plausible reaction pathways are shown in Fig. 7d. ARplorer proposed the most plausible reaction mechanism for the Lewis-acid-promoted cycloaddition reaction, including a concerted pathway not previously discussed^44^. This concerted cycloaddition pathway undergoes TS **A-TS3** (0.6 kcal/mol), which is nearly equal to the literature-proposed stepwise reaction pathway (path A) via **A-TS2** (0.2 kcal/mol) and **A-IM2-TS1** (-5.3 kcal/mol).

**Fig. 8:**
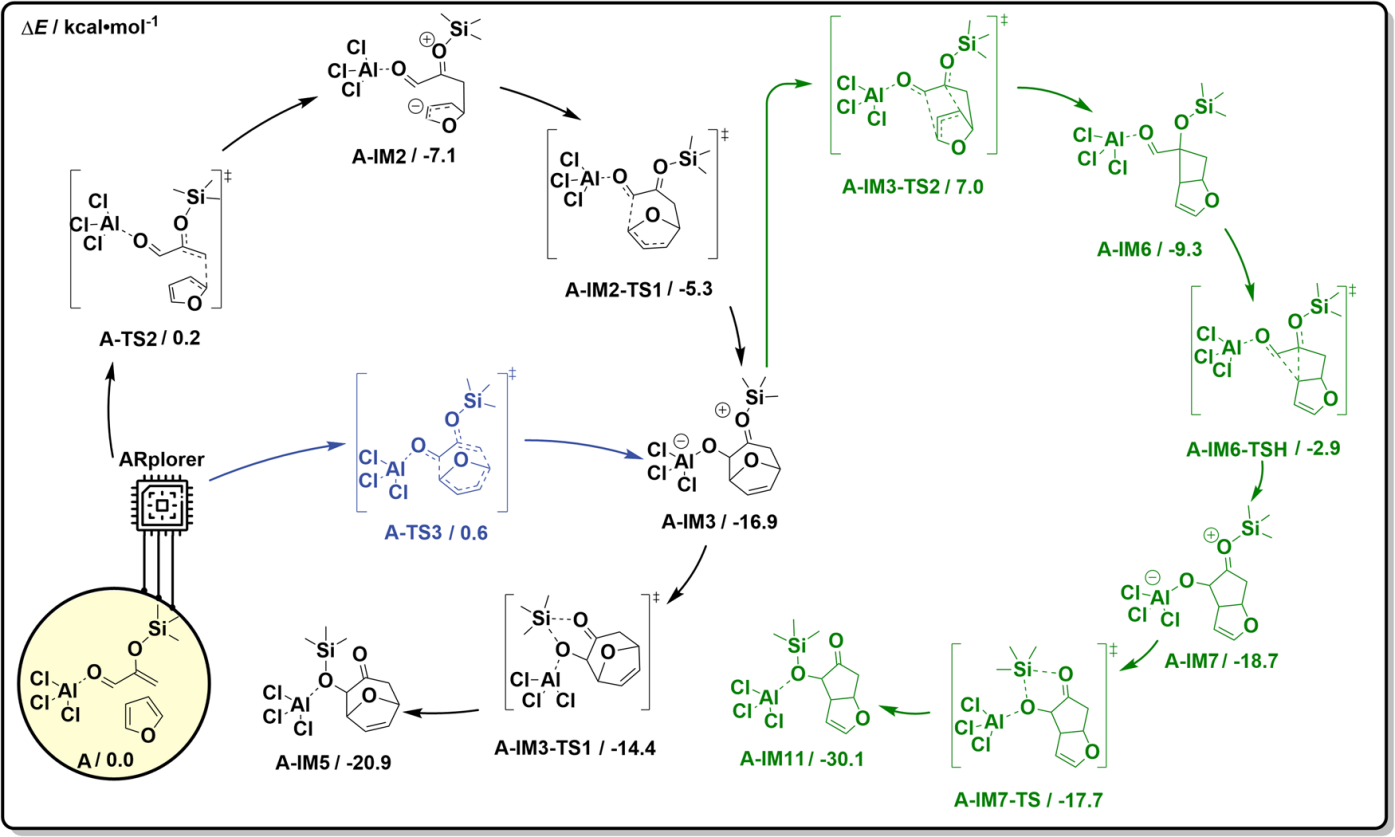
ARplorer proposed the most plausible reaction mechanism for the Lewis-acid-promoted cycloaddition reaction. The black pathway is the previously reported one reproduced by ARplorer, the blue pathway highlights a previously unreported concerted channel, and the green pathway presents a previously unreported side reaction.

Significantly, ARplorer uncovered a new side reaction, which was yet unexplored in the literature, shown as the green reaction pathway in Fig. 8, starting from **A-IM-3**. This reaction proceeds through a rearrangement via TS **A-IM3-TS2** (7.0 kcal/mol) to form an oxabicyclo[3.2.0]heptane intermediate **A-IM6** (−9.3 kcal/mol). This [2 + 2] cycloaddition product **A-IM6** could not be directly formed from **A** because of the Woodward–Hoffmann rules, or from **A-IM2** due to ring constraint. Then, a 1,2-migration via TS **A-IM6-TS** (-2.9 kcal/mol) would result in a cyclopenta[b]furan intermediate **A-IM7** (−18.7 kcal/mol) and the final thermodynamically more stable product **A-IM11**(-30.1 kcal/mol). Although this side pathway (in green) presents a higher barrier compared to the [4 + 2] cycloaddition, it suggests a potential variation toward a thermodynamically controlled product under different reaction conditions, not considered in previous manual DFT calculations. This example well demonstrates how our large language model-guided ARplorer can serve as a fast and effective automated tool for exploring complex reaction mechanisms and contributing to the data-driven development of reactions and catalyst design.

### Case 2: the Asymmetric Mannich-type Reaction

**Fig. 9:**
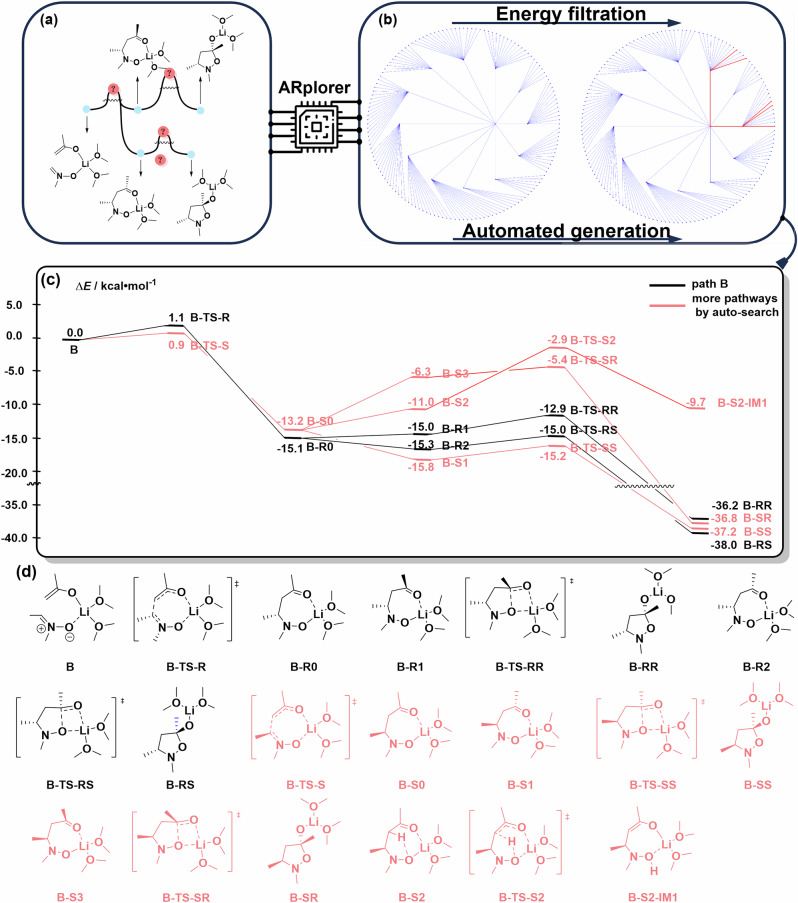
Important reaction pathways and structures of the PES disclosed by ARplored in Case 2. **a**. Reported reaction pathways. **b** Overall network of exploration of reaction pathways by ARplorer. **c**. More plausible reaction pathways generated by ARplorer, the black path is the path B generated by ARplorer. **d**. Selected structures.

Our ARplorer program shows decent potential in handling challenging asymmetric reactions, as evidenced by its application to the study of an asymmetric Mannich-type reaction^45^. The previously discussed reaction pathways in the literature (path B) are shown in Fig. 9a. The chirality function of the program was turned on when letting **B** as the IN and running the program for automated exploration. The comprehensive exploration of the stereoselective reaction pathways of the asymmetric Mannich-type reaction required only 0.25 days of computer time on a 24-core (Xeon GOLD 6342) server. The entire automated exploration network of the whole reaction pathways (Fig. 9b) includes 156 reaction steps because only a few active sites exist in this reaction system. The primary reaction pathways (filtered by 0.04 Hartree of reaction barrier) were shown in Fig. 9c, with the selected key structures depicted in Fig. 9d.

**Fig. 10:**
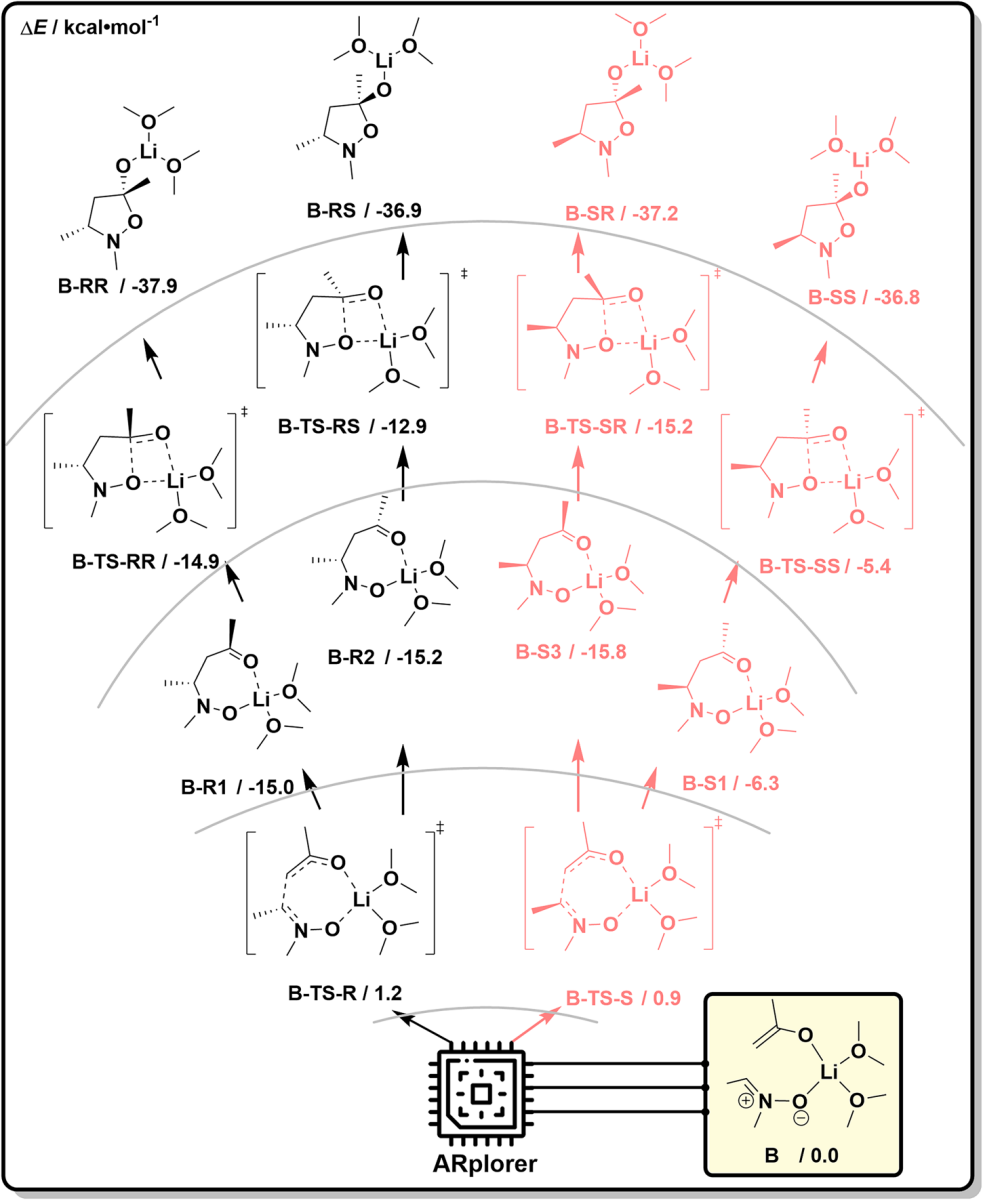
ARplorer proposed the most plausible reaction mechanism for the asymmetric Mannich-type reaction. Black pathways indicate the previously reported ones that reproduced by ARplorer, while red pathways represent additional stereoselective channels mapped out by ARplorer automated exploration.

The results of the large language model-guided automated exploration of asymmetric pathways are summarized in Fig. 10. ARplorer precisely produced the proposed reaction pathways by manual DFT calculations, as shown in the black reaction pathways in Fig. 10. Crucially, it systematically and efficiently mapped all stereoselective pathways in a remarkably short timeframe. The exploration covered all enantio- and diastereoselective pathways towards **B-SS, B-SR, B-RS**, and **B-RR** products. While further refinement with high-level theory and consideration of solvation effects were not yet incorporated in this preliminary exploration with ARplorer, these findings clearly highlight ARplorer’s potential for rapidly and effectively screening and designing asymmetric catalytic systems.

### Case study 3: platinum-catalyzed annulation of alkynes to indoles

**Fig. 11:**
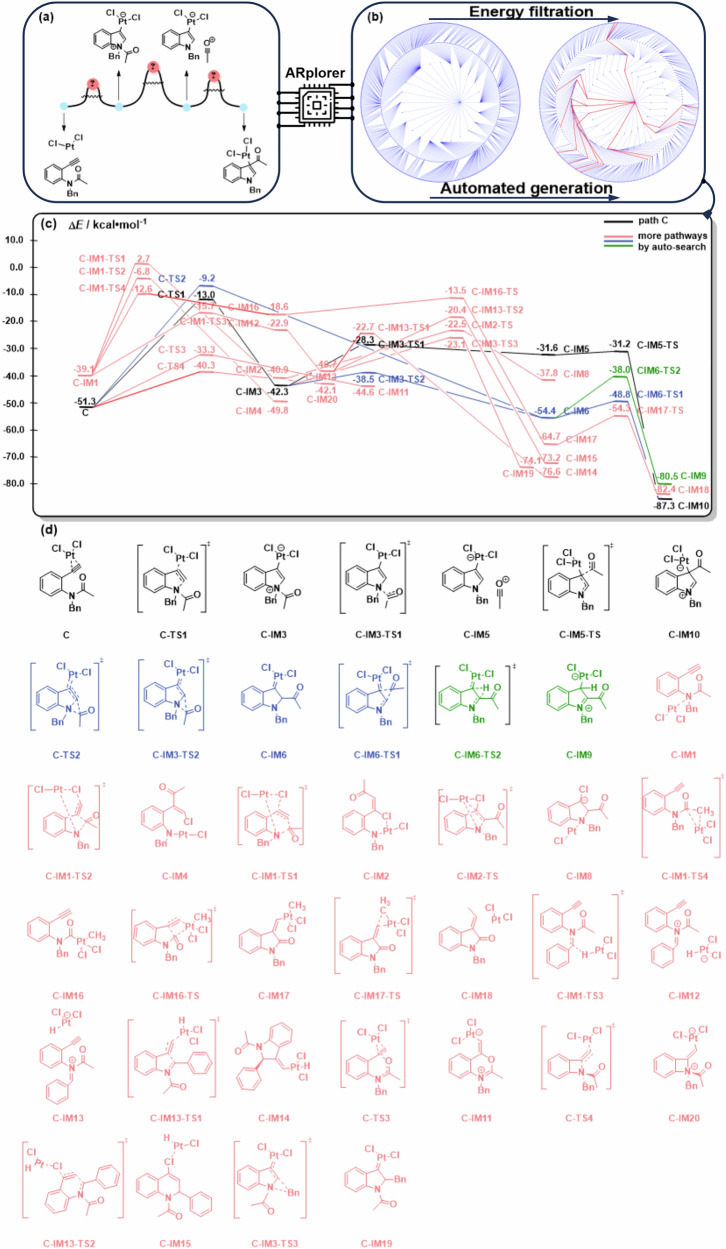
Important reaction pathways and structures of the PES disclosed by ARplored in Case 3. **a** Reported reaction pathways. **b** Overall network of exploration of reaction pathways by ARplorer. **c** The important reaction pathways generated by ARplorer, the black path (the path C) is the most plausible. **d** Selected structures.

Transition metal-catalyzed reactions present one of the most important fields in both industries and academia. However, the reaction pathway exploration and mechanism study of transition metal catalysis is a huge challenge. Especially, when we come to more complicated reactants, it is a laborious task to systematically find the intermediates and TS for all possible reaction pathways, due to the explosive growth of reaction combinations by chemical logic. Herein, we tested our ARplorer program on the platinum-catalyzed annulation of alkynes to indoles^46^, a process complicated by multiple reactive sites in the *N*-(2-ethynylphenyl)acetamide reactant. The mechanism previously suggested in the literature is shown in Fig. 11a, proposing the formation of an acylium intermediate for further intramolecular reaction with indol-3-yl platinum species.

To our delight, the large language model guided ARplorer shows excellent capability in setup and extensively explores all possible reaction pathways for transition metal-catalyzed reactions. Completing the exploration and mechanism determination required only 4.5 days of computational time on a 24-core (Xeon GOLD 6342) server. The automated exploration network of the whole reaction pathways including 2693 reaction steps is shown in Fig. 11b. The major reaction pathways (filtered by 0.08 Hartree of reaction barrier) finally generated by ARplorer were depicted in Fig. 11c. As our expectation, ARplorer accurately replicated the reaction pathway through the acylium intermediate (PES in black), aligning with the previously reported mechanism (Path C)^46^.

Moreover, ARplorer’s extensive search yielded additional pathways beyond those identified in manual DFT calculations, offering deeper insights into the mechanism of the platinum-catalyzed annulation of alkynes to indoles. Notably, ARplorer proposed an alternative mechanism involving an intramolecular acyl group 1,2-migration from intermediate **C-IM3** through TS **C-IM3-TS2** to **C-IM6**. This intramolecular acyl migration TS **C-IM3-TS2** (-38.5 kcal/mol) is kinetically much more favored than the acyl dissociation TS **C-IM3-TS1** (−28.3 kcal/mol). The resulting intermediate **C-IM6** (−54.4 kcal/mol), a typical Fischer-type carbene with a platinum-carbon double bond, is thermodynamically more stable than the previously suggested acylium intermediate **C-IM5** (−31.6 kcal/mol). This carbene further undergoes another intramolecular acyl 1,2-migration to form the final product **C-IM10** (−87.3 kcal/mol).

**Fig. 12:**
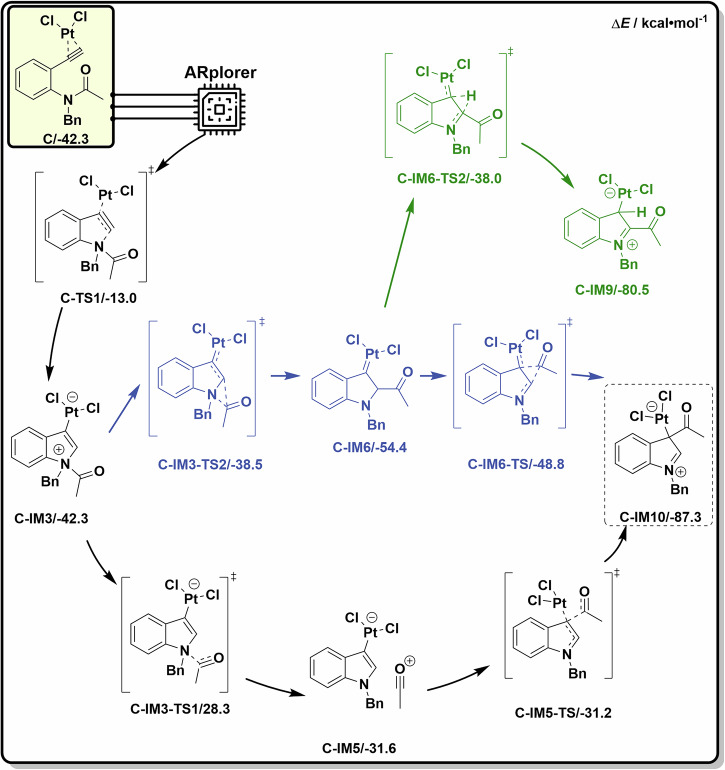
ARplorer proposed the most plausible reaction mechanism for platinum-catalyzed annulation of alkynes to indoles. The black pathway is the previously reported one reproduced by ARplorer, the blue pathway highlights a newly identified intramolecular acyl-migration mechanism involved a platinum carbenoid intermediate, and the green pathway presents a previously unreported side reaction.

ARplorer also discovered many other important side reaction pathways (in red and green color, Fig. 11c, d), enriching our understanding of the reaction mechanism. For instance, a 1,2-hydride migration initiated from the platinum carbenoid **C-IM6** to the electrophilic carbon is a widely discussed mechanism for Fischer-type carbene; however, it is found to be a less favored side reaction here, with a located TS **C-IM6-TS2** with a calculated energy of −38.0 kcal/mol. Summarized in Fig. 12, the extensive, however fast and effective, exploration of ARplorer not only reproduced the previously suggested acylium reaction pathway (in black) in literature but also unfolded an intramolecular acyl-migration mechanism via platinum carbenoid (in blue), as well as other important side reaction pathways (in green for example). Although the kinetic and thermodynamic difference between the formation of acylium intermediate and carbene intermediate vary depending on the solvent effect, it is really an amazing surprise that the systematic exploration with ARplorer was able to discover such a classical and theoretically plausible carbene mechanism, which was not discussed by manual DFT calculations, to help further understand the platinum-catalyzed annulation reaction of alkynes toward indoles. This example demonstrated the extraordinary advantages of ARplorer in an automated, fast, efficient, and systematic exploration of complicated transition metal-catalyzed reactions and transition metal catalyst designs.

### Limitations

There are still some limitations in this work. The ARplorer program, driven by LLM-guided chemical logic, has a chemical logic library that is universal for finding many kinds of elementary reactions, and is suitable for finding homogeneous reaction systems with complex reaction paths, but for some elementary reactions and special synthons, these reaction logic libraries should be further expanded. This is particularly relevant when dealing with systems of varying multiplicity throughout the reaction, suggesting that the chemical logic should be enhanced in future updates. Although the implementation of efficient energy filtration and parallel computing boosts the feasibility and efficiency of the program, the computational complexity still scales exponentially with the number of steps in the reaction pathway. In our studies, the solvation effect and high-level energy refinement were neglected in the process of automated reaction pathway exploration for efficiency. However, incorporating high-level energy refinements and corrections for solvation effects remains a viable and straightforward option in ARplorer, or through other well-established programs like Gaussian^41^ or ORCA^47^, etc., allowing users to balance between accuracy and computational demand according to their project needs.

## Conclusion

Reaction pathway exploration for PES study has been a popular topic in computational chemistry for a long time. Addressing the existing challenges of PES search methods, including time-consuming processes, impracticality due to exponential growth, and the potential for irrelevant searches, we introduce a general tool built on Python and Fortran designed for automated, fast, and efficient PES study of reaction pathways. Our present ARplorer program merges the features of QM approaches and rule-based approaches, performing an automated multi-step search of reaction pathways under the guidance of LLM-based chemical logic. This integration of large language model-assisted chemical logic provides an expandable library for thorough reaction pathway exploration, enhancing PES search efficiency by bypassing futile searches. Active-learning methods in TS sampling help efficiently judge the feasibility of elementary steps. Parallel multi-step reaction searches with efficient filtering, controlled exploration breadth. The effectiveness and utility of ARplorer in automated searches are exemplified through multi-step reactions, including organic cycloaddition, asymmetric Mannich-type reactions, and organometallic Pt-catalyzed reactions. Despite some inherent limitations, our ARplorer program offers excellent advantages in fast and efficient automated exploration of reaction pathways, which should not only be crucial for mechanism research and chemical synthesis innovation, and serve as an inspiration for data-driven reaction development and catalyst design. In future developments, we plan to enhance the reaction logic library by incorporating condition-specific annotations (e.g., temperature/pressure-sensitive reaction types) and enabling post-screening thermodynamic adjustments (e.g., ΔG^‡^ and solvation effect calculations) for user-defined conditions.

## Method

### Computational details

All the calculations were performed by our developed ARplorer program, in which the reaction rules and chemical logic were encoded through SMARTS patterns derived from literature and LLM-guided strategies, with molecular manipulation and active site identification facilitated by Pybel. For computational efficiency, all the efficient potential energy surface exploration was conducted using the semiempirical QM methods GFN2-xTB^42^, and more accurate DFT methods (B3LYP^48,49^/Lanl2DZ^50,51^ choosen herein) were used for higher-level validation when necessarily. All the structures along PES were verified to be the minima or TS by frequency analysis calculations. IRC calculations were also performed to confirm the connection of the TS to two relevant minima.

## Supplementary information

Supplementary Information

## Data Availability

The data that support the findings of this study are provided in the Supporting Information.

## Code availability

All relevant codes are available from the corresponding authors upon reasonable request and with the authors’ permission.
