# Author Correction: Large language model guided automated reaction pathway exploration

**DOI:** 10.1038/s42004-025-01788-5

**Published:** 2025-11-19

**Authors:** Ruzhao Chen, Yubang Liu, Zhe Chen, Yinwu Li, Fuyi Yang, Jiaxin Lin, Zhuofeng Ke

**Affiliations:** School of Materials Science and Engineering, PCFM Lab, the Key Laboratory of Low-carbon Chemistry & Energy Conservation of Guangdong Province, Sun Yat-sen University, Guangzhou, P. R. China

**Keywords:** Computational chemistry, Reaction mechanisms, Reaction mechanisms

Correction to: *Communications Chemistry* 10.1038/s42004-025-01630-y, published online 24 August 2025.

In the version of this article initially published, the image shown as Fig. 11 was an inadvertent duplicate of Fig. 7. The correct Fig. 11 now appears in the HTML and PDF versions of the article.

