# Supplementary material for: Large language model guided automated reaction pathway exploration
Source: Commun Chem. 2025 Aug 24;8:255. doi: 10.1038/s42004-025-01630-y (PMC12375046; doi:10.1038/s42004-025-01630-y)
Supplement: Supplementary file 1 — Supplementary Information [file 42004_2025_1630_MOESM1_ESM.pdf]

Electronic Supplementary Information

Large Language Model Guided Automated Reaction Pathway Exploration

Ruzhao Chen, Yubang Liu, Zhe Chen, Yinwu Li, Fuyi Yang, Jiaxin Lin, and Zhuofeng Ke*

School of Materials Science and Engineering, PCFM Lab, the Key Laboratory of Low-carbon Chemistry & Energy Conservation of Guangdong Province, Sun Yat-sen University, Guangzhou 510006, China

* Correspondence： kezhf3@mail.sysu.edu.cn

Content

Original chemical logic from a Large Language Model 2
Functions of customized potential U 8
Postprocessing 9
Detail process of active-Learning Approach for Sampling Transition State Locations. 10
Curation of Chemical Logic library 13
TS benchmark 17
Comparsion between ARplorer and other existing programs of automated reaction pathways search 18
Variance test of ChatGPT. 20
Stochasticity test of ARplorer 22
Overview of the ARplorer workflow 24
Design of Chain-of-through (COT) prompt 24
DFT-optimized cartesian coordinates of all species involved in the cycloaddition reaction 26
DFT-optimized cartesian coordinates of all species involved in the asymmetric Mannich-type reaction 46
DFT-optimized cartesian coordinates of all species involved in the platinum-catalyzed reaction 65

Original chemical logic from a Large Language Model

The original chemical logic could be classified into general chemical logic and specified chemical logic. The original chemical logic used in this program is constituted by general organic chemical (**Figure S1**) logic and general transition metal chemical logic (**Figure S2**). Both organic and transition metal chemical logic could be generated in detail by interacting with ChatGPT further, where Electrophilic active sites (**Figure S3**) and Bond strain active sites (**Figure S4**) are shown as examples. Then for each specified system performed with ARplorer, specified chemical logic associated with the system would be generated using ChatGPT (The specified chemical logic of three examples shown in this paper are shown in **Figure S5**, **Figure S6**, **Figure S7**). Both the general chemical logic and specified chemical logic constitute the system-specified chemical logic. All the chemical logic would be coded with SMARTS rules and used to guide the direction of automatic reaction search in ARplorer.

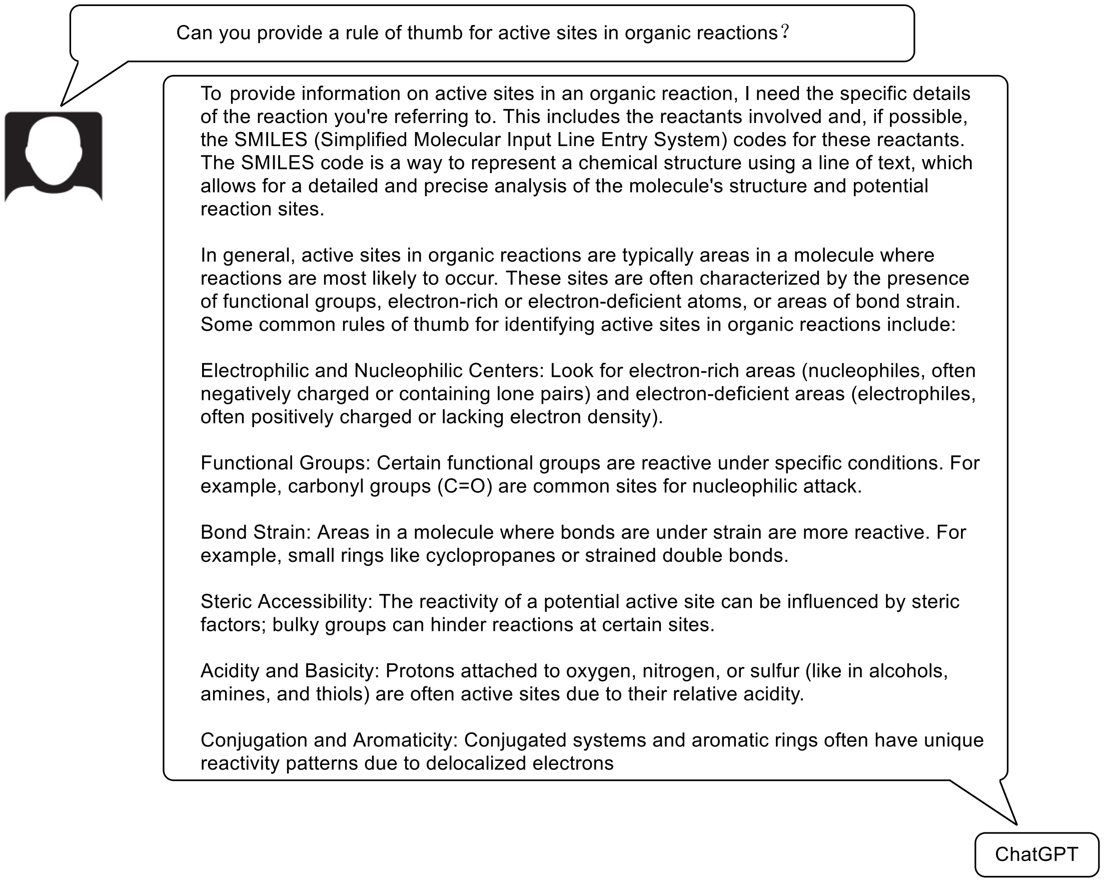

Figure S1. Create generalized organic chemical logic from ChatGPT

please focus on the active sites or reaction patterns in reaction involves transition metals, provide as many examples as possible

Reactions involving transition metals play a crucial role in organic chemistry, particularly in catalysis. Transition metals can facilitate a variety of reactions due to their ability to adopt multiple oxidation states and to form complex geometries. They often act as catalysts by providing a site for substrates to bind and react. Here are several examples illustrating active sites or reaction patterns in reactions involving transition metals:

Hydrogenation Reactions (Pd, Pt, Ni): Transition metals like palladium, platinum, and nickel are commonly used in hydrogenation reactions. They adsorb hydrogen and the unsaturated substrate (like an alkene or alkyne), facilitating the addition of hydrogen across the double or triple bond.

Cross-Coupling Reactions (Pd, Ni): Palladium and nickel catalyze cross-coupling reactions, such as Suzuki, Heck, and Negishi couplings. These reactions typically involve the formation of a new carbon-carbon bond between an organohalide and an organometallic reagent.

Olefin Metathesis (Ru, W, Mo): Ruthenium, tungsten, and molybdenum catalysts are used in olefin metathesis, where double bonds between carbons are broken and reformed, exchanging substituents.

Hydroformylation (Rh, Co): Rhodium and cobalt catalysts are employed in hydroformylation, where an alkene, carbon monoxide, and hydrogen react to form an aldehyde. The transition metal facilitates the formation of an acyl metal intermediate.

C-H Activation (Rh, Pd, Ir): Transition metals like rhodium, palladium, and iridium can activate C-H bonds in hydrocarbons, a process used in functionalization reactions to form new C-C, C-N, C-O bonds, etc.

Oxidation Reactions (Mn, Cr, Ru): Manganese, chromium, and ruthenium are used in oxidation reactions. For example, KMnO4 (potassium permanganate) is used for the oxidation of alcohols.

Alkene Epoxidation (Ti, Mo, Re): Transition metals like titanium, molybdenum, and rhenium are catalysts in alkene epoxidation, where an alkene is converted to an epoxide.

Carbon Monoxide Insertions (Co, Ni, Fe): In reactions like carbonylation, transition metals like cobalt, nickel, and iron can facilitate the insertion of carbon monoxide into organic substrates.

Cyanation Reactions (Ni, Pd): Nickel and palladium catalysts are used in cyanation reactions, where a cyanide group is introduced into organic substrates.

Alkyne Cyclotrimerization (Co, Ni, Mo): Transition metals like cobalt, nickel, and molybdenum can catalyze the cyclotrimerization of alkynes to form aromatic rings.

In each of these reactions, the transition metal acts as a catalyst by coordinating to substrates, altering their electronic structure, and lowering the activation energy for the reaction. This coordination often involves empty d-orbitals of the metal and can include oxidative addition, reductive elimination, migratory insertion, and ligand exchange as key steps in the catalytic cycle. Transition metal-catalyzed reactions are central to modern synthetic chemistry due to their versatility and efficiency.

ChatGPT

Figure S2. Create generalized chemical logic with transition metals from ChatGPT

please focus on the active sites in reaction involves electronphilic , provide as many examples as possible,
only provide the example with active fragments and functioanl groups

In reactions involving electrophilic active sites, the electrophile is a fragment or functional group that can accept an electron pair. These electrophiles are often central to many important organic reactions, as they seek out electron-rich regions (nucleophiles) to form new chemical bonds. Here are examples of electrophilic active sites, focusing on the functional groups or fragments that act as the electrophiles:

Carbocations (R-C+): A classic example of electrophiles. Carbocations are central to many reactions, such as Friedel-Crafts alkylation, where an alkyl halide forms a carbocation intermediate that reacts with an aromatic ring.

Aldehydes and Ketones (R-C=O): The carbonyl carbon in aldehydes and ketones is electrophilic due to the partial positive charge. They are key players in nucleophilic addition reactions, like the Grignard reaction or aldol condensation.

Alkyl Halides (R-X): In these compounds, the carbon bonded to a halogen (like Cl, Br, or I) is electrophilic. They are commonly used in nucleophilic substitution reactions (SN1 and SN2).

Acyl Chlorides (R-CO-Cl): The carbonyl carbon in acyl chlorides is highly electrophilic and readily reacts with nucleophiles, making them useful in acylation reactions like Friedel-Crafts acylation.

Sulfonic Acids (R-SO3H): The sulfur atom in sulfonic acids and its derivatives (like sulfonyl chlorides) can act as an electrophile, especially in sulfonation reactions.

Epoxides (R-O-R'): The strained three-membered ring in epoxides makes the carbon atoms electrophilic, leading to ring-opening reactions with various nucleophiles.

Nitronium Ion (NO2+): A powerful electrophile used in nitration reactions, typically formed in situ from nitric acid and sulfuric acid.

Proton (H+): Although a simple ion, the proton is a common electrophile in acid-base reactions, and in catalytic processes where an acid catalyst donates a proton to an organic substrate.

Alkenes (R-CH=CH-R') in Electrophilic Addition: In the presence of strong acids, the alkene double bond can become electrophilic, leading to electrophilic addition reactions.

Carbon Dioxide (CO2): While not electrophilic in its ground state, under certain conditions, CO2 can act as an electrophile, reacting with nucleophiles like Grignard reagents to form carboxylic acids.

ChatGPT

is there more active fragments and functioanl groups

Indeed, there are more examples and types of electrophilic active sites in organic chemistry, each with distinct reactivity profiles. Here are additional examples, focusing on specific functional groups or fragments that act as electrophiles:

Nitriles (R-C≡N): The carbon atom in nitriles has a partial positive charge due to the electronegativity of nitrogen, making it an electrophilic site for reactions like nucleophilic addition.

Isocyanates (R-N=C=O): The electrophilic carbon in isocyanates can react with various nucleophiles, leading to products like ureas and carbamates.

Carbon Disulfide (CS2): Similar to carbon dioxide, carbon disulfide can behave as an electrophile, reacting with nucleophiles such as amines.

Sulfur Dioxide (SO2): In certain reactions, sulfur dioxide can act as an electrophile, especially in the presence of chlorosulfonic acid or other activating agents.

Electrophilic Aromatic Substitution: In this class of reactions, the aromatic ring acts as a nucleophile. Electrophiles include nitronium ion (NO2+), sulfonyl chlorides (RSO2Cl), and acylium ions (RC+=O), among others.

Imine (R-C=N-R'): The carbon-nitrogen double bond in imines makes the carbon electrophilic, facilitating nucleophilic attacks, particularly in the formation of enamines and iminium ions.

Alkylidene Malonates (R-CH=C(COOR)2): The α-carbon next to the carbonyl group is electrophilic due to the electron-withdrawing effect of adjacent ester groups.

Acid Anhydrides (R-C(O)OC(O)-R'): The carbonyl carbon in acid anhydrides is electrophilic and can react with various nucleophiles, similar to acyl chlorides.

Quinones (O=C6H4=O): The carbonyl groups in quinones are electrophilic and can undergo nucleophilic addition, particularly in redox reactions.

Halogenation Reagents: Reagents like N-bromosuccinimide (NBS) or N-chlorosuccinimide (NCS) can generate electrophilic bromine or chlorine species, used in selective halogenation reactions.

Michael Acceptors: Compounds with an α,β-unsaturated carbonyl group (like enones or acrylates) act as electrophiles in Michael addition reactions, where the β-carbon is the electrophilic site.

Tosylates and Mesylates (ROTs, ROMs): These groups convert alcohols into good leaving groups, creating an electrophilic carbon center that is susceptible to nucleophilic attack.

ChatGPT

Anhydrides of Phosphoric Acid (e.g., P2O5): These act as strong electrophiles, often used in dehydration reactions or as activating agents in nucleotide synthesis.

Silyl Chlorides (R3SiCl): Used in the protection of alcohols and other nucleophilic sites. The silicon atom is electrophilic due to its attachment to chlorine.

Alkylboranes (R-BH2): In hydroboration-oxidation reactions, the boron atom in alkylboranes is electrophilic, facilitating the addition to alkenes.

Acid Halides of Phosphorus (e.g., POCl3): These compounds are electrophilic, often used in the conversion of alcohols to chlorides or as a reagent in the Vilsmeier-Haack reaction.

Disulfides (R-SS-R'): The sulfur atoms in disulfides can act as electrophiles, particularly in redox reactions or when activated by oxidizing agents.

Trialkylaluminum Compounds (R3Al): These organometallic compounds have electrophilic aluminum atoms and are used in various organic reactions, including polymerization and alkylation.

Electrophilic Selenium Compounds (e.g., SeO2): Selenium dioxide is an example where selenium acts as an electrophile, particularly in allylic oxidation reactions.

Peracids (RC(O)OOH): In these compounds, the oxygen atom is electrophilic and is used in epoxidation reactions and other oxidations.

Organic Azides (R-N3): In certain reactions, the terminal nitrogen atom in azides can behave as an electrophile, particularly in cycloaddition reactions.

Acyl Imidazoles (R-CO-Imidazole): These are activated forms of carboxylic acids where the acyl group is electrophilic and readily reacts with nucleophiles.

Carbonyl Compounds with α-Electrophilic Atoms: Compounds like chloral (CCl3CHO), where the α-carbon is highly electrophilic due to the presence of electron-withdrawing halogens.

Diazonium Salts (Ar-N2+X-): The aromatic diazonium group acts as an electrophile in reactions like Sandmeyer or Gomberg-Bachmann reactions.

ChatGPT

Figure S3. Electrophilic active site in organic chemical logic from ChatGPT

please focus on the active sites in Bond Strain, provide as many examples as possible

Focusing on active sites related to bond strain, we find that areas of high strain in a molecule are typically more reactive. This is because the atoms in these strained regions are often in unfavorable configurations, making them more susceptible to chemical reactions as a way to relieve the strain. Here are several examples illustrating how bond strain can create active sites:

Small Ring Strain: Cyclopropanes and cyclobutanes are classic examples. The bond angles in these small rings deviate significantly from the ideal tetrahedral angle (109.5°), creating significant angle strain. This makes them more reactive compared to larger rings like cyclohexanes, which can adopt conformations that minimize strain.

Bicyclic Strain: Bicyclo compounds, especially those with bridgehead carbons like in bicyclo[2.2.1]heptane (norbornane), experience significant strain. The bridgehead carbons are forced into an almost planar arrangement, creating angle strain and making these sites more reactive.

Transannular Strain: In medium-sized rings (8-12 members), transannular (across the ring) interactions can lead to strain. For example, in cyclodecane, the hydrogen atoms on opposite sides of the ring can be in close proximity, leading to steric hindrance and increased reactivity.

Allylic Strain: In conjugated dienes, the double bonds can cause allylic strain. This strain can make the allylic positions (the carbons adjacent to the double bonds) more reactive, particularly in reactions like allylic bromination.

Torsional Strain in Alkenes: In certain alkenes, especially those with bulky substituents, torsional strain can arise due to eclipsing conformations. This strain can increase the reactivity of the double bond.

Axial vs Equatorial Strain in Cyclohexanes: In substituted cyclohexanes, substituents in the axial position experience more steric strain compared to those in the equatorial position. This can affect the reactivity of the substituents and the ring itself.

Baeyer Strain in Polycyclic Compounds: Named after Adolf von Baeyer, this strain is observed in polycyclic compounds where the rings are fused in a way that forces the atoms into unfavorable angles, increasing reactivity.

Ring Strain in Epoxides: Epoxides, or three-membered cyclic ethers, have significant angle strain due to the small ring size. This makes the epoxide ring highly reactive, particularly towards nucleophilic opening reactions.

Strain in Bridgehead Alkenes: Bridgehead double bonds in bicyclic systems (like in bicyclo[2.2.2]oct-2-ene) are highly strained due to the inability of the double bond to adopt the ideal planar geometry, increasing their reactivity.

Spiro Compounds Strain: In spiro compounds, where two rings are connected through a single atom, the shared atom experiences strain due to the difficulty in satisfying the ideal geometry for both rings.

ChatGPT

Figure S4. Bond strain active site in organic chemical logic from ChatGPT

Explain the active sites in the reaction.
"""""""" c1ccco1.[CH2]/C(=C/O[Al](Cl)(Cl)Cl)/O[Si](C)(C)C """"""""

The SMILES code "c1ccco1.[CH2]/C(=C/OAl(Cl)Cl)/OSi(C)C" represents a chemical reaction involving two distinct molecules. Let's dissect this code to understand the structures and identify the potential active sites:

1. c1ccco1: This is a furan ring, a five-membered aromatic ring with one oxygen and four carbon atoms. The aromatic nature of furan suggests that it could undergo electrophilic aromatic substitution reactions, but furans are also known for participating in nucleophilic addition due to the electron-rich oxygen atom.
2. [CH2]/C(=C/O[Al](Cl)(Cl)Cl)/O[Si](C)(C)C: This is a more complex molecule. It features several functional groups:

- [CH2]: A methylene group, which could be a site for nucleophilic attack, especially if it is adjacent to electron-withdrawing groups.
- C(=C/O[Al](Cl)(Cl)Cl): This part of the molecule is an aluminum alkoxide complex with a vinyl group adjacent to it. The aluminum center, bonded to oxygen and three chlorides, is likely to be electrophilic due to the electron-withdrawing nature of the chloride atoms.
- O[Si](C)(C)C: This is a silyl ether group. Silicon, being less electronegative than carbon, makes the oxygen slightly electron-rich. However, silyl ethers are generally stable and less reactive under normal conditions.

Considering these structures, the potential active sites in this reaction are:

- Furan Ring (c1ccco1): The furan oxygen can act as a nucleophile, and the aromatic system might undergo electrophilic aromatic substitution.
- Methylene Group ([CH2]): This could be a site for nucleophilic attack, depending on its position relative to other groups.
- Aluminum Center (OAl(Cl)Cl): The aluminum alkoxide is likely electrophilic, making it a potential site for nucleophilic attack, especially by oxygen-containing nucleophiles like the furan ring.

ChatGPT

Figure S5. Specified chemical logic about example of cycloaddition reaction from ChatGPT

Explain the active sites in the reaction.
"""" N([O])([CH]C)C.C(=C)(O[Li])C.O(C)C.O(C)C """"

The SMILES code you've provided represents a chemical reaction. To analyze the active sites, we need to break down the code into its respective reactants and understand the chemical functionalities involved. The SMILES code "N([O])([CH]C)C.C(=C)(O[Li])C.O(C)C.O(C)C" appears to describe a reaction with multiple reactants. Let's dissect it:

1. N([O])([CH]C)C: This part of the SMILES code represents a nitrogen atom bonded to an oxygen atom, a methylene group (CH2) attached to another carbon, and another carbon atom. The presence of nitrogen with an adjacent oxygen suggests a potential for nucleophilic activity, particularly at the nitrogen or the methylene carbon.
2. C(=C)(O[Li])C: This fragment represents a lithium alkoxide with a vinyl group. The lithium cation (Li+) is likely to be associated with the alkoxide oxygen, making it an electrophilic center due to the polarization of the C-O bond. The vinyl group (C=C) could be involved in addition reactions.
3. O(C)C: This structure is repeated twice in the SMILES code and represents an ether, specifically a dialkyl ether. Ethers typically are not very reactive, but they can be involved in reactions under certain conditions, like cleavage by strong acids.

Considering the reaction as a whole, the active sites likely involve:

- Nucleophilic Center: The nitrogen or the methylene carbon in N([O])([CH]C)C could act as a nucleophile.
- Electrophilic Center: The lithium alkoxide oxygen in C(=C)(O[Li])C presents an electrophilic site due to its association with the lithium cation.
- Vinyl Group: The vinyl group in C(=C)(O[Li])C could participate in addition reactions, potentially with the nucleophilic nitrogen or carbon mentioned earlier.

ChatGPT

Figure S6. Specified chemical logic about example of asymmetric Mannich-type reaction from ChatGPT

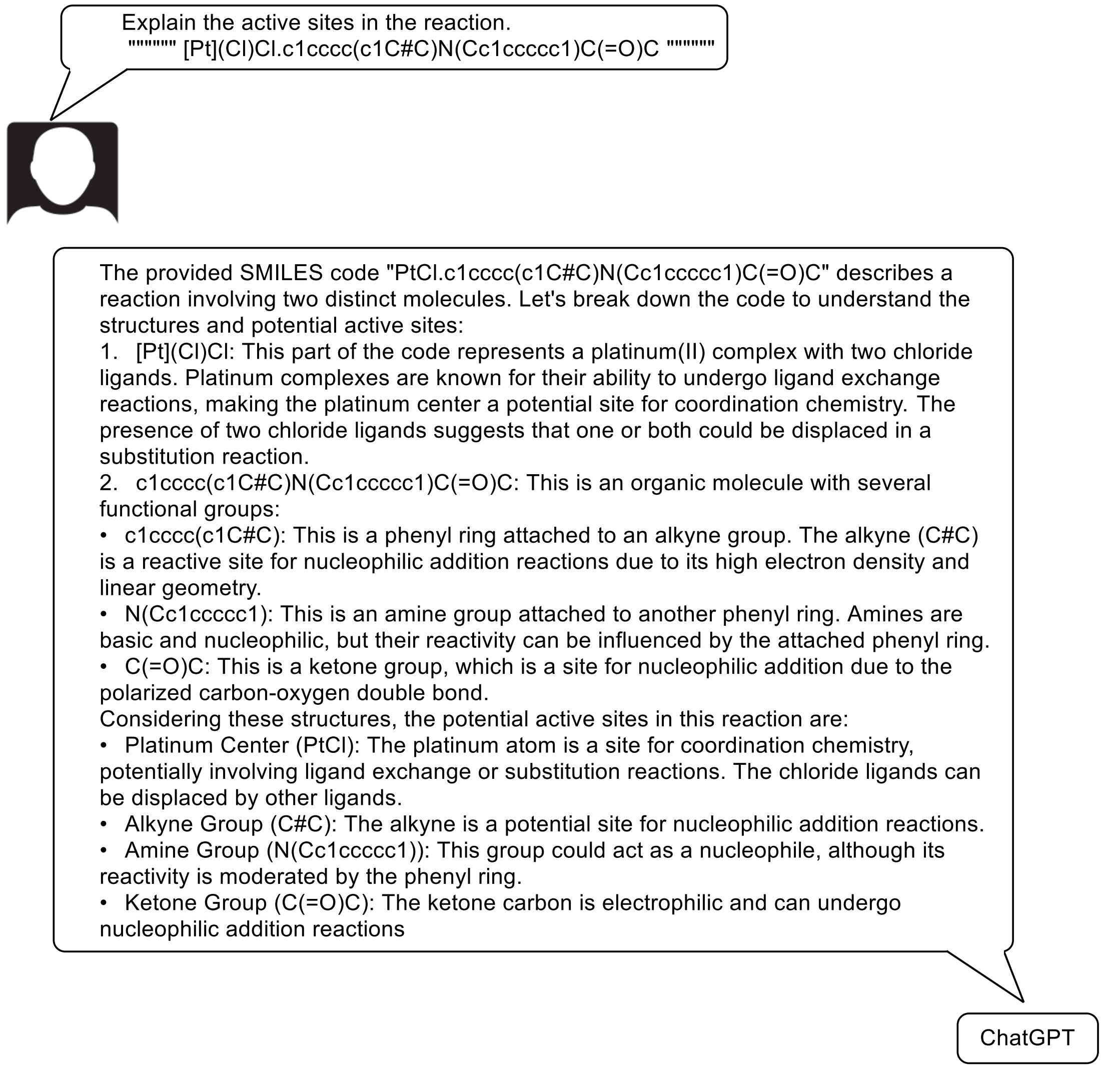

Figure S7. Specified chemical logic about example of asymmetric Mannich-type reaction from ChatGPT

Functions of customized potential U

Figure S8 shows the preparation and the mechanism to use U in optimizing structure and the main applications of U in this program.

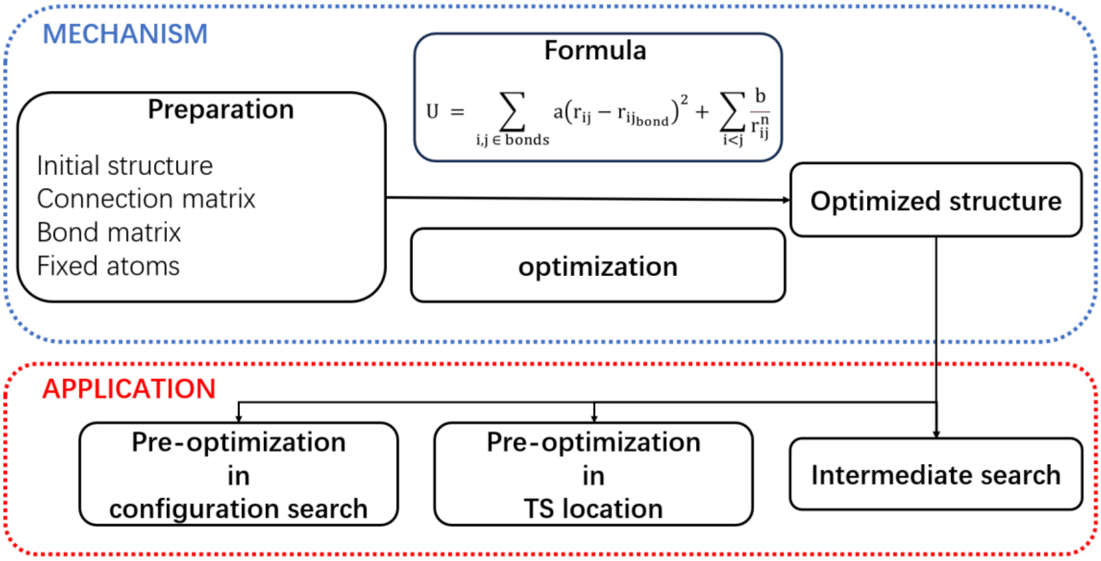

Figure S8. Functions of customized potential U.

Postprocessing

The postprocessing of the program is following. As shown in **Figure S9**, when the set of total reaction paths was got, it would be filtered by certain energy barrier which is set manually and a new set of filtered reaction paths forms. Then this set would be sorted by energy barrier and classified by the structure of final intermediate. Finally, a diagram of each filtered reaction path would be produced automatically.

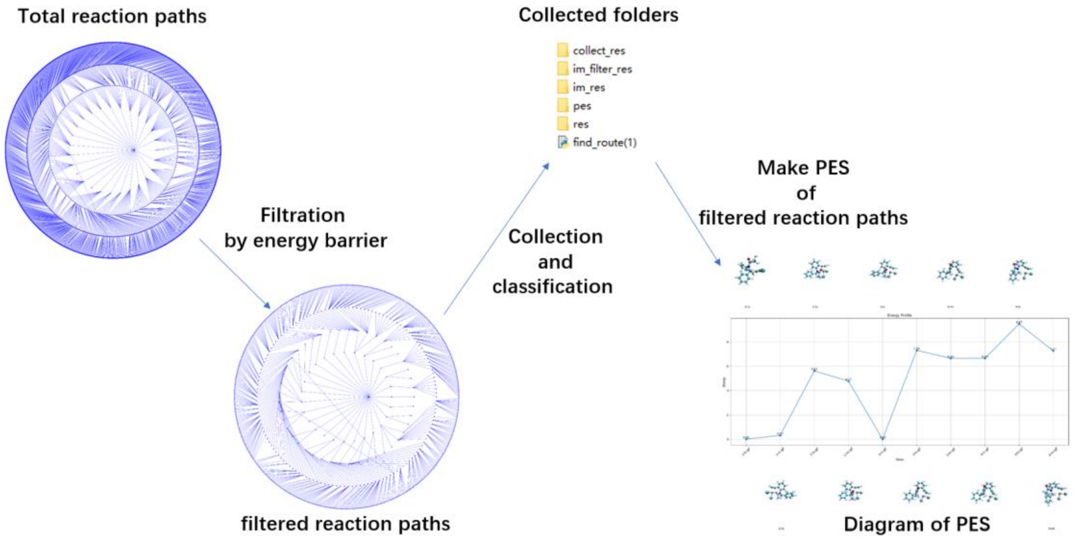

Figure S9. Workflow of postprocessing in the program.

Detail process of active-Learning Approach for Sampling Transition State Locations.

This method is a single-end method, as shown in **Figure S10-1**.

1. For a given IN (beginning from IM is similar), two probe structures were constructed in the first golden ratio point (the one is close to IN) of the range default reasonable distance (as described in **Response 2-11**). Then, both probe structures were optimised with the active site constrained.
2. Three consecutive probe points including the one with highest energy would be fitted into a quadratic function curve. The extreme point, which was close to one of the previous probes in terms of conformation space (0.1Å), would be guessed as the potential saddle point.
3. If a qualified TS was not obtained, this failed TS would be served as new probe point and the program return to step 2 until a qualified TS was found or reached the maximum step.

If a qualified TS could not be located when IN was a beginning point, similar process beginning from IM would be conducted.

Take a simple elementary reaction for an example. Active learning sampling took 7 probe points to locate the qualified TS. As for the normal scan method, the traditional scan should involve a twenty-step search to find the qualified TS (curve d) (**Figure S10-1**). Active learning sampling is more efficient.

It is worth noting that the quadratic function curve used here does not mean the shape around the TS is always quadratic-like. The quadratic curve is a way to explore the potential energy surface widely rather than search the potential energy surface step by step (we do not mean the normal scan method is not a good approach to locate TS). This method is different from other existing methods, and according to the result above, the active learning sample is a practical approach to efficiently locate TS.

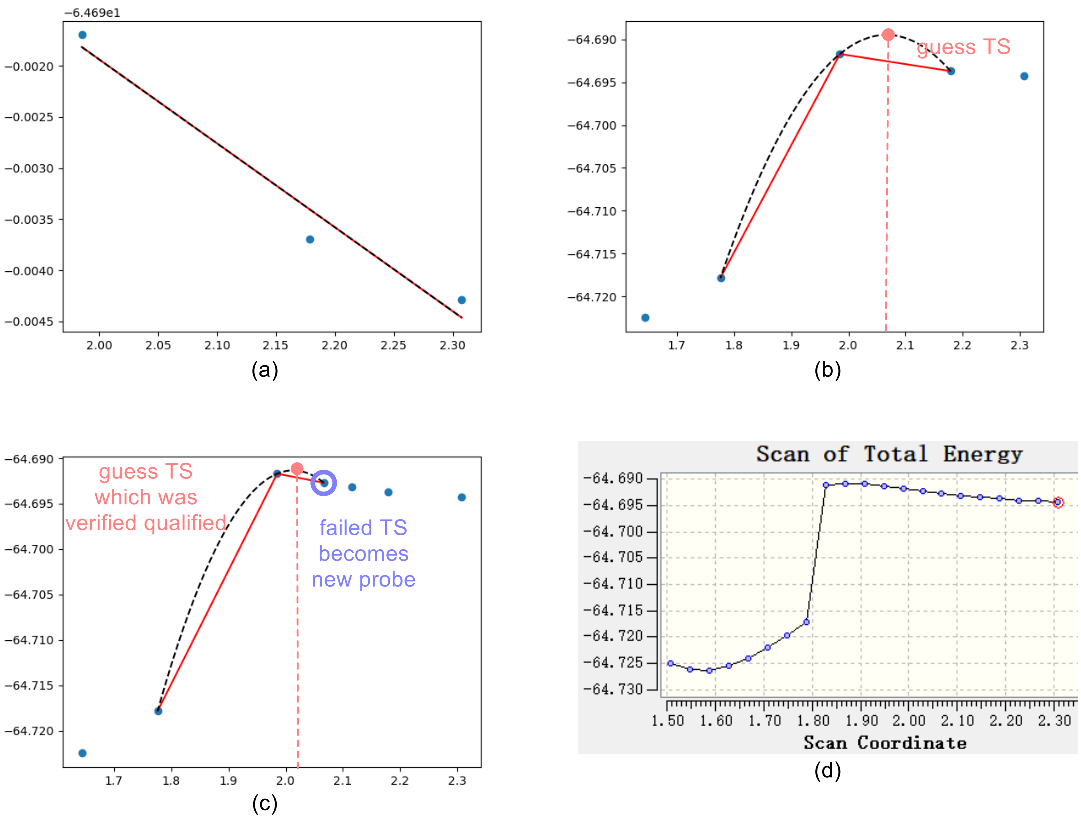

Figure 10-1. presentation of comparison between active learning sampling and normal scan approach for a simple elementary reaction. From (a) to (c) is a presentation of step 2 to step 4. (d) is the normal scan for TS location.

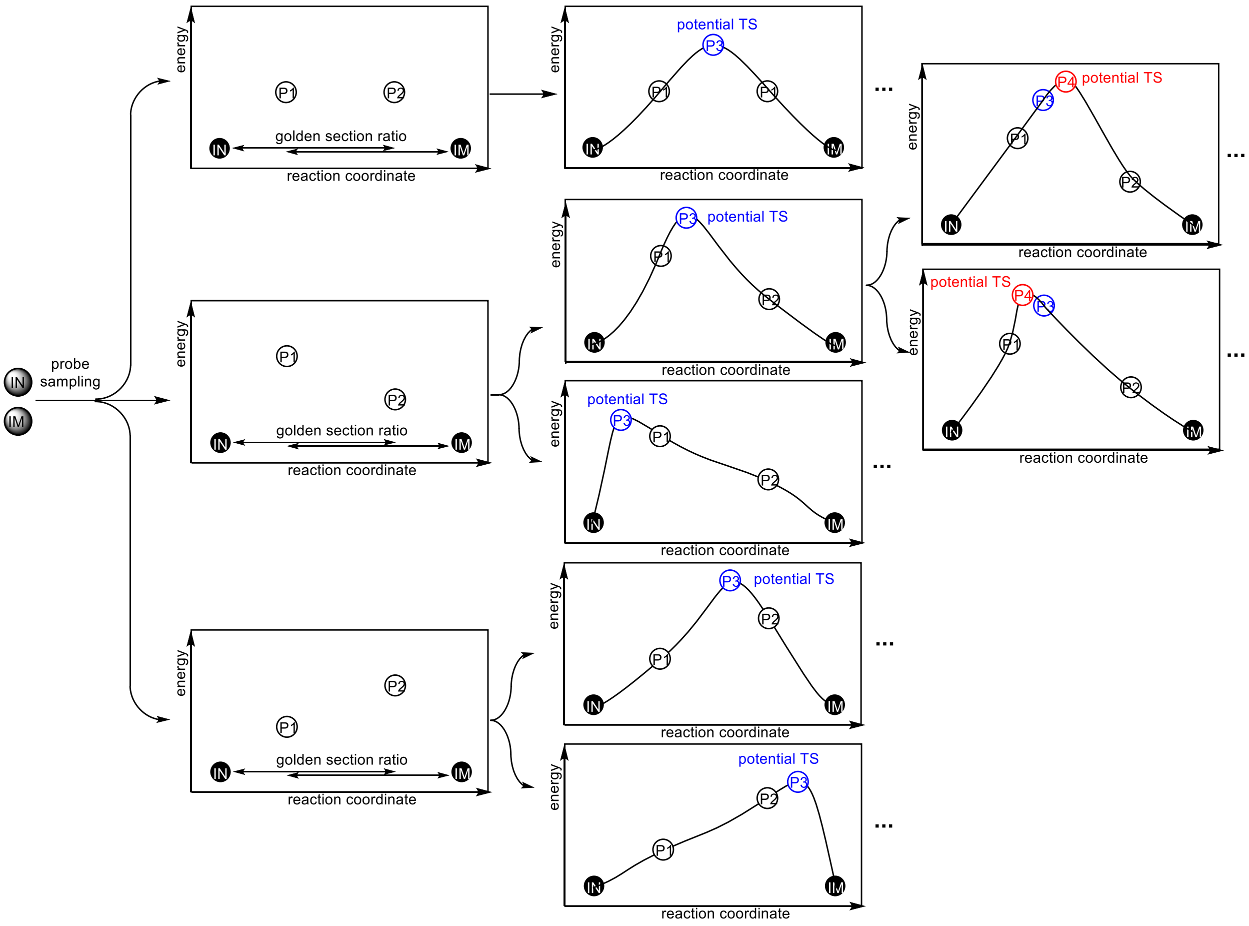

Figure S10-2. Detailed explanation of active-Learning Approach for Sampling Transition State Locations

Curation of Chemical Logic library

Table S1. Curated Chemical Logic Library(partial) from different reaction type

Reaction Type	Description	Extract SMARTS	Library
Michaelis–Arbuzov Reaction	A reaction where a trivalent phosphorus ester reacts with an alkyl halide to form a pentavalent phosphorus compound and another alkyl halide.	[P;H2,H1]~[C;H0][X;H0]	[$([P;H2,H1]~[C;H0][I,Cl,Br,F])]
Appel Reaction	Converts alcohols into alkyl halides using triphenylphosphine and a tetrahalomethane.	[O;H1]~[C;H0,H1]	[$(O[$([H])])]-[H] [$(O[$([H])]),$(O=*)]
Alder–Ene Reaction	A pericyclic reaction between an alkene with an allylic hydrogen and a compound containing a multiple bond, resulting in a new σ-bond and a shifted double bond.	[C;R]=[C;R],[C;R]=[C;R]	[$([CX3]~[$([CX3])])]-[!$([CX3])] [$([CX3]~[$([CX3])])]
Acyloin Condensation	A reductive coupling of two esters using metallic sodium to yield an α-hydroxyketone (acyloin).	[C;H1,H2]([O;H0])=[O;H0]	[$(C=[OX1])] [$(O[$([H])]),$(O=*)]

Acetoacetic Ester Synthesis	A method to synthesize methyl ketones by alkylating ethyl acetoacetate followed by hydrolysis and decarboxylation.	[C;H1,H2]([C;H0]=[O;H0])[C;H0]=[O;H0]	[$(O[$([H])]),$(O=*)]
Wittig Reaction	Transforms aldehydes or ketones into alkenes using a phosphonium ylide.	[P]=[C;H0],[#6][OX2]=O	[$(C=[OX1])] [$(O[$([H])]),$(O=*)]
Ullmann Reaction	A copper-catalyzed coupling of aryl halides to form biaryl compounds.	[C;R]([c;H0])[X;H0]	[$(C;R(-[c;H0])-[I,Br,Cl,F;H0))]
Ugi Reaction	A four-component condensation reaction involving an aldehyde, amine, carboxylic acid, and isocyanide to form α-aminoacyl amide derivatives.	[N;H2],[C;H0]=[O;H0]	[$(N[$([H])]),$(O[$([H])]),$(S[$([H])]),$(N=*),$(O=*),$(S=*),$([OX1]!#*)]
Swern Oxidation	Oxidizes primary and secondary alcohols to aldehydes and ketones using DMSO and oxalyl chloride under mild conditions.	[C;H0](=O)[O;H0],[C;H0]([O;H0])(=O)	[$(C=[OX1])] [$(O[$([H])]),$(O=*)]
Shi Epoxidation	An asymmetric epoxidation of alkenes using a fructose-derived organocatalyst and oxone as the oxidant.	[C;R]=[C;R],[O;H0]-[O;H0]	[$([CX3]~[$([CX3])])]-[!$([CX3])] [$([CX3]~[$([CX3])])]
Fischer Indole Synthesis	Produces indoles from phenylhydrazines and aldehydes	[C;H0]=[O;H0],[N;H2]~[c;H0]	[$(C=[OX1])] [$(N[$([H])]),$(O[$([H])]),$(S[$([H])]),$(N=*),$(O=*),$(S=*),$([OX1]!#*)]

	or ketones under acidic conditions.		
Fleming–Tamao Oxidation	Converts carbon–silicon bonds to carbon–oxygen bonds using peroxy acids or hydrogen peroxide.	[Si;H0]~[c;H0],[Si;H0]~[X;H0]	[$(Si;H0-[$([c;H0]),$(I,Cl,Br,F;H0)])]
Fischer Esterification	Forms esters by reacting carboxylic acids with alcohols in the presence of an acid catalyst.	[C;H0](=O)[O;H1],[C;H0][O;H1]	[$(C=[OX1])] [$(O[$([H])]),$(O=*)]
Friedländer Synthesis	Synthesizes quinoline derivatives from 2-aminobenzaldehydes and ketones.	[N;H2]~[C;H0]=[O;H0],[C;H0]=[C;H1]	[$(C=[OX1])] [$(N[$([H])]),$(O[$([H])]),$(S[$([H])]),$(N=*),$(O=*),$(S=*),$([OX1]!#*)]
Fukuyama Coupling Reaction	A palladium-catalyzed coupling of thioesters with organozinc reagents to form ketones.	[S;H0]~[C;H0]=[O;H0],[Zn;H0]~[C;H0]	[$(C=[OX1])] [$(N[$([H])]),$(O[$([H])]),$(S[$([H])]),$(N=*),$(O=*),$(S=*),$([OX1]!#*)]
Grignard Reaction	Forms carbon–carbon bonds by reacting organomagnesium halides with electrophilic carbon atoms.	[C;Mg],[#6][OX2]=O	[$(C=[OX1])] [$(O[$([H])]),$(O=*)]
Hofmann Rule	Predicts that in elimination reactions, the less substituted alkene will be the major product.	[N;H0]([C;H0])([C;H0])([C;H0])	[$(N;H0([C;H0])([C;H0])([C;H0]))]
Negishi Coupling Reaction	A palladium- or nickel-catalyzed cross-coupling of organozinc	[Zn;H0]-[C;H0],[*][X;H0]	[$(Zn;H0-[C;H0]),$(*-[X;H0])]

	compounds with organic halides to form carbon–carbon bonds.		
Sakurai Allylation Reaction	Adds allyl groups to aldehydes or ketones using allylsilanes in the presence of a Lewis acid.	[C;H0]=[C;H0],[Si;H0]~[C;H0]=[C;H0]	[$([CX3]~[$([CX3])])]-[!$([CX3])] [$([CX3]~[$([CX3])])]
Reformatsky Reaction	Forms β-hydroxy esters by reacting α-halo esters with aldehydes or ketones in the presence of zinc.	[C;H0][Br;H0],[C;H0][O;H0]	[$(C;H0-[Br;H0]),$(C;H0-[O;H0])]

TS benchmark

The ARplorer could discover reactions in different manual-unexplored systems rather than DFT-unexplored ones. It is easy to understand that a manual search of reaction pathways is generally incomplete and negligent. Besides, to verify the effectiveness of active learning sampling on TS location, our method was applied to the reaction database of RMG. From this database, some reactions involving the change of multiplicity were excluded, and 59 reactions were chosen. The result of active learning sampling on TS location on these 59 reactions is shown in **Table S2**. Active learning sampling on TS location used in ARplorer presented well in general situations of TS location. This test on the RMG database has been supplied with supporting information.

Table S2. Benchmark of active learning sampling on TS location

Entry	Reaction	Success (√) or failure (×)
1	[1+2] Cycloaddition	√
2	1,2-Insertion (CO)	√
3	1,2-Insertion (Carbene)	×
4	1,2-Elimination (NH_3)	√
5	1,2-Shift (C)	√
6	1,2-Shift (S)	√
7	1,2-Interchange	√
8	1,3-Insertion (ROR)	√
9	1,3-Insertion (RSR)	√
10	1,3-Insertion (CO_2)	×
11	1,3-Elimination (NH_3)	√
12	1,3-Sigmatropic Rearrangement	√
13	[2+2] Cycloaddition	√
14	6-Membered Central C-C Shift	√
15	Baeyer-Villiger Reaction Step1 (**Cat**)	√
16	Baeyer-Villiger Reaction Step2	√
17	Baeyer-Villiger Reaction Step2 (**Cat**)	√
18	Br atom Abstraction	√
19	Cl atom Abstraction	√
20	Cyclic-Formation (Ether)	√
21	Cyclic-Formation (Thioether)	√
22	Scission (Cyclopentadiene)	√
23	Diels-Alder Addition	√
24	Diels-Alder Addition (Aromatic)	√
25	F Atom Abstraction	√
26	Elimination (Peroxy Radical)	√
27	H Atom Abstraction	√
28	Intra-[2+2] Cycloaddition	√

29	Intra-5-Membered Conjugated Addition (C=C-C=C)	√
30	Intra-Diels-Alder (Monocyclic)	√
31	Concerted Intra-Diels-Alder (Monocyclic 1,2-Shift)	×
32	Intra-Ene Reaction	√
33	Intra-Halogen Migration	√
34	Intra-H Migration	√
35	Intra-OH Migration	√
36	Intra-Retro-Diels-Alder Reaction (Bicyclic)	√
37	Intra-RH-Addition (Endocyclic)	√
38	Intra-RH-Addition (Exocyclic)	√
39	Intra-R-Addition (Endocyclic)	√
40	Intra-R-Addition (Exocyclic)	√
41	Intra-R-Addition (ExoTetCyclic)	√
42	Intra-R-Addition (Exo Scission)	×
43	Intra-Substitution Cyclization (CS)	√
44	Intra-Substitution Isomerization (CS)	√
45	Intra-Substitution Cyclization (S)	√
46	Intra-Substitution Isomerization (S)	√
47	Ketoenol	√
48	Korcek Reaction Step1	√
49	Korcek Reaction Step2	×
50	Korcek Reaction Step1 (Cat)	√
51	Retroene	√
52	R-Addition (CO)	√
53	R-Addition (CS)	√
54	R-Addition (MultipleBond)	√
55	Singlet-Carbene Intra-Disproportionation	√
56	Substitution (S)	√
57	Substitution (O)	√
58	XY-Addition (MultipleBond)	√
59	XY-Elimination (Hydroxyl)	×
Total number: 59; Succeed cases: 53; **Successful Rate: 90%**		

Comparsion between ARplorer and other existing programs of automated reaction pathways search

To show the distinct features of ARplorer, a comparison of ARplorer with other existing automated reaction pathways search programs (Kinbot, ADCR, GRRM and LASP) was performed through the pathways search of a representative organic reaction, the Claisen rearrangement of CH_2=CH-O-CH_2-CH=CH_2, and a transition metal-catalyzed reaction,

platinum-catalyzed annulation of alkynes to indoles. The results are shown in **Table S3-1** and **Table S3-2**.

ADCR is a MD-QM combined reaction pathways search program, GRRM is QM-based global reaction route mapping program, and LASP is based on large-scale atomistic simulation with neural network potential. ARplorer and Kinbot are rule-based automated reaction pathways search programs. As for the organic reaction (**Table S3-1**), It could be seen that the outcome yield (it means the obtained complete elementary reactions, including IN, TS, IM and PC, among the total searches) of the rule-based search is generally more efficient than the global search because the chemical logic or the rules could exclude many unimportant processes. LASP, based on machine learning potential, is also efficient in reaction pathways search; however, only limited elements were supported by LASP. The outcome yield of ARplorer is the highest among the tested programs, and it shows the efficiency of ARplorer in reaction pathway search.

As for the system with transition metal atoms, as shown in **Table S3-2**, the QM-based programs that use global search approaches, such as ADCR and GRRM, became heavily cost and made it difficult to obtain complete results of accessible reaction pathways for relatively larger systems. The Kinbot and LASP are limited, and the catalytic system with transition metal is unsupported in these two programs. According to the above comparison, ARplorer can be used as an automatic reaction pathway search program with broad applicability, high generality and high efficiency.

Table S3-1. Comparison between ARplorer and other programs
(Claisen rearrangement of $CH_2=CH-O-CH_2-CH=CH_2$)

Program	Total reactions	Outcome reaction	Outcome yield	Total number of TSs	Total number IMs	Costed Time (h)	Calculation level
ARplorer	427	156	36.5%	156	178	5	xTB (GFN-2)
ADCR	24949	221	0.8%	221	282	18.9	xTB (GFN-2)
GRRM	-	200	-	200	197	168	B3LYP /6-31G
Kinbot	220	64	29.1%	34	-	72	B3LYP /6-31G
LASP	1575	303	19.2%	303	2182	8	MLP

In ARplorer and ADCR, the reaction is specified to proceed in two reaction steps. In other programs, reaction steps could not be specified, and these programs were terminated at a given time. In ADCR, four of the twelve atoms were frozen. The site with “-” means this data could not be found.

Table S3-2. Comparison between ARplorer and existing programs
(platinum-catalyzed annulation of alkynes to indoles)

Program	result	Calculation level
ARplorer	As shown in Case 3 of the paper	xTB (GFN-2)
ADCR	Too costly and no output in 5 days	xTB

		(GFN-2)
Kinbot	Transition metal elements are not supported	B3LYP /6-31G
LASP	ML potential for transition metal was not found	MLP

Variance test of ChatGPT.

Three repeat consecutive rounds of production of chemical logic of case 2 were used to test the variance of the specific ChatGPT, as shown below in **Figure S11**, **S12**, **S13**. It could be seen that the variance introduced by this specific ChatGPT is minimal and does not significantly affect the extracted chemical logic

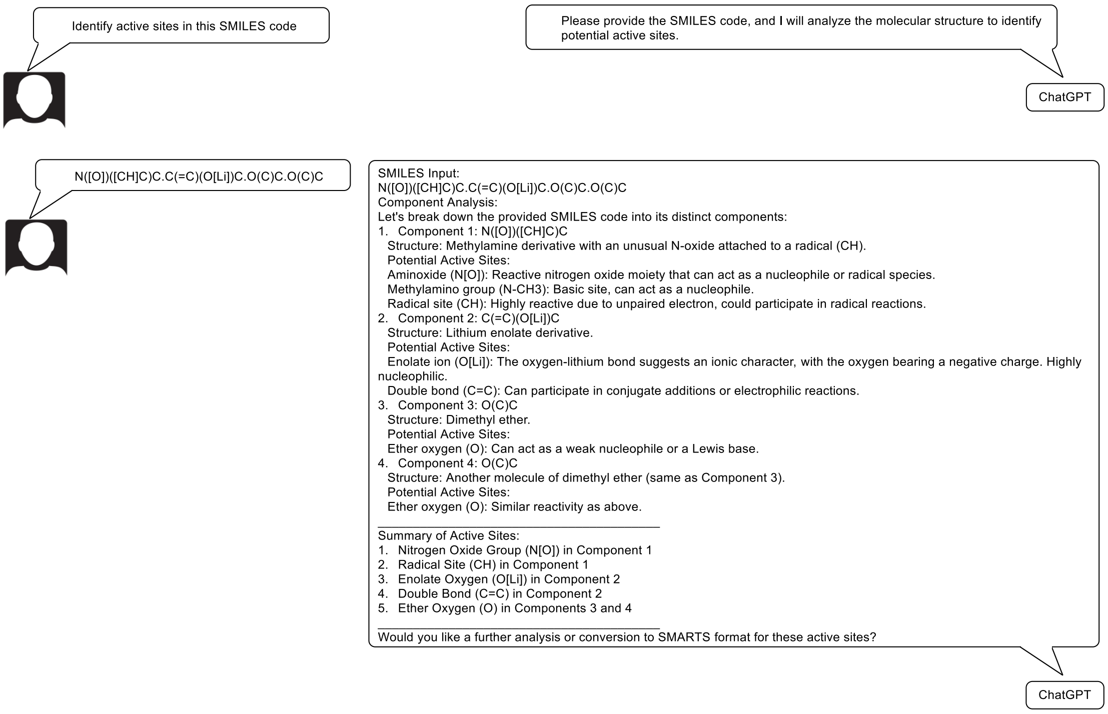

Figure S11. The first round of the variance test of the chemical logic of case 2.

N([O])([CH]C)C.C(=C)(O[Li])C.O(C)C.O(C)C

SMILES Input:
N([O])([CH]C)C.C(=C)(O[Li])C.O(C)C.O(C)C
Component Analysis:
The provided SMILES code represents a mixture of four distinct molecular components. Let's analyze each one to identify potential active sites:

Component Breakdown:

1. Component 1: N([O])([CH]C)C
   Structure: A methylamine derivative with a nitrogen oxide group.
   Potential Active Sites:
   Aminoxide Group (N[O]): This nitrogen-oxide linkage is reactive and can participate in nucleophilic reactions or act as a radical source.
   Methylamino Group (N-CH3): The nitrogen with an attached methyl group may act as a basic or nucleophilic site.
   Radical Carbon ([CH]C): The presence of an unpaired electron (indicated by [CH]) on the carbon suggests high reactivity, possibly in radical reactions.
2. Component 2: C(=C)(O[Li])C
   Structure: A lithium enolate derivative.
   Potential Active Sites:
   Enolate Ion (O[Li]): The oxygen bonded to lithium indicates a negative charge localized on oxygen, making it a strong nucleophile.
   Double Bond (C=C): The carbon-carbon double bond is electron-rich and can be involved in electrophilic or conjugate addition reactions.
3. Component 3: O(C)C
   Structure: Dimethyl ether.
   Potential Active Sites:
   Ether Oxygen (O): The oxygen atom can act as a weak nucleophile or a Lewis base, but it is generally less reactive than other functional groups.
4. Component 4: O(C)C
   Structure: Identical to Component 3 (dimethyl ether).
   Potential Active Sites:
   Ether Oxygen (O): Same reactivity as above.

Summary of Active Sites:
Component Active Site Description
1 (N([O])([CH]C)C) N[O] Nitrogen-oxide group, nucleophilic or radical site
1 (N([O])([CH]C)C) [CH] Radical carbon site
2 (C(=C)(O[Li])C) O[Li] Enolate oxygen, strong nucleophile
2 (C(=C)(O[Li])C) C=C Double bond, electrophilic reaction site
3 & 4 (O(C)C) O Ether oxygen, weak nucleophile/Lewis base

Would you like a SMARTS pattern for these identified active sites, or do you have further questions about potential reactivity?

ChatGPT

Figure S12. The second round of the variance test of the chemical logic of case 2.

N([O])([CH]C)C.C(=C)(O[Li])C.O(C)C.O(C)C

SMILES Input:
N([O])([CH]C)C.C(=C)(O[Li])C.O(C)C.O(C)C
Thank you for providing the SMILES code once again. Let's ensure a clear and accurate analysis of the components and their active sites.

Component Analysis:
The given SMILES represents four separate chemical species. We'll identify each molecule and pinpoint their reactive centers.

1. Component 1: N([O])([CH]C)C
   Structure: A methylamine derivative with a nitrogen oxide substituent and a radical site.
   Active Sites:
   Aminoxide Group (N[O]): The nitrogen-oxide functionality is a highly reactive group, potentially acting as a nucleophile or radical species.
   Radical Carbon ([CH]): The carbon with an unpaired electron indicates radical reactivity.
   Amine Nitrogen (N-CH3): This nitrogen atom could act as a base or a nucleophile.
2. Component 2: C(=C)(O[Li])C
   Structure: A lithium enolate with a central double bond.
   Active Sites:
   Enolate Oxygen (O[Li]): The negatively charged oxygen (complexed with lithium) is a strong nucleophile, likely to participate in nucleophilic addition reactions.
   Double Bond (C=C): The alkene can participate in electrophilic additions or conjugate reactions.
3. Component 3: O(C)C
   Structure: Dimethyl ether.
   Active Sites:
   Ether Oxygen (O): Though typically unreactive, the oxygen can act as a weak nucleophile or Lewis base in the presence of strong electrophiles.
4. Component 4: O(C)C
   Structure: Another molecule of dimethyl ether (same as Component 3).
   Active Sites:
   Ether Oxygen (O): Same reactivity profile as above.

Summary of Identified Active Sites:
Component Active Site Description
N([O])([CH]C)C N[O] Nitrogen-oxide, nucleophilic or radical site
N([O])([CH]C)C [CH] Radical carbon, highly reactive
C(=C)(O[Li])C O[Li] Enolate oxygen, strong nucleophile
C(=C)(O[Li])C C=C Double bond, reactive toward electrophiles
O(C)C & O(C)C O Ether oxygen, weak nucleophile/Lewis base

Would you like to proceed with generating SMARTS patterns for these identified active sites?

ChatGPT

Figure S13. The third round of the variance test of the chemical logic of case 2.

To test if there is obvious stochasticity in ARplorer, a replicate run of case 2 and tests of change of energy threshold have been performed. The results are in Table 2, and the

corresponding PES are in Figure R1. No obvious stochasticity exists in the reaction pathways search with ARplorer. The parameters of the energy threshold mainly affect the total number of reactions found and barely affect the reaction with a low energy barrier.

Stochasticity test of ARplorer

To test if there is obvious stochasticity in ARplorer, a replicate run of case 2 and tests of change of energy threshold have been performed. The results are in **Table S4,** and the corresponding PES are in **Figure S14**. No obvious stochasticity exists in the reaction pathways search with ARplorer. The parameters of the energy threshold mainly affect the total number of reactions found and barely affect the reaction with a low energy barrier.

Table S4. Test of stochasticity (Case 2)

	IM_energy threshold (a.u.)	IRC_energy threshold (a.u.)	Number of reactions
original	0.08	0.2	156
Replication	0.08	0.2	156
Test1	0.04	0.1	29
Test2	0.08	0.1	55

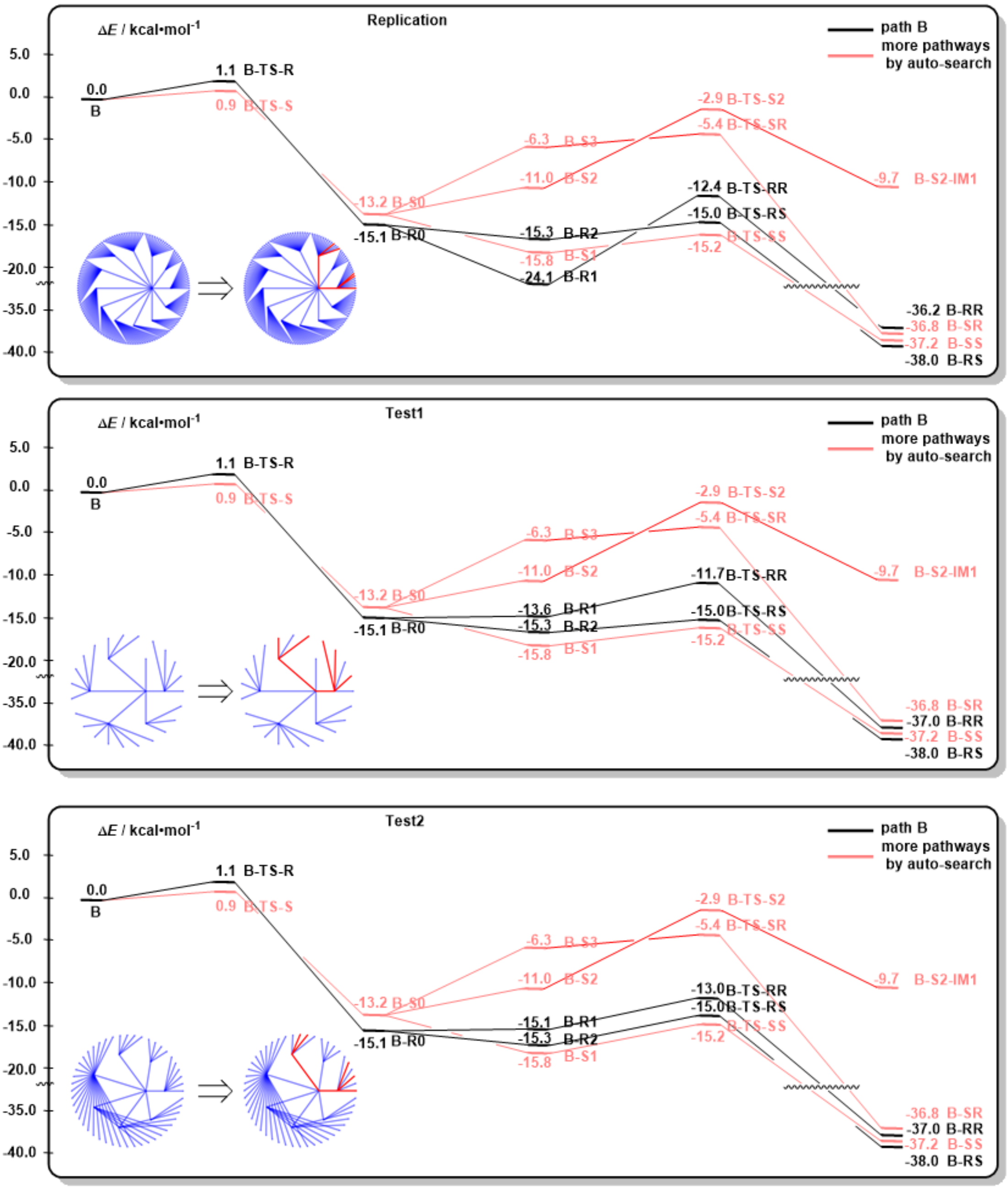

Figure S14 Reaction pathways with low energy barriers in replication test1 and test2.

Overview of the ARplorer workflow

To clarify the mechanism by which the chemical logic base and active-site library are integrated within ARplorer, an overview of the ARplorer workflow is presented in Figure S15. As depicted, the workflow begins with the systematic collection and curation of relevant documents, such as books, patents, and scientific literature (step 1). In step 2, text-mining and data-processing techniques are employed to extract key chemical logic terms, reaction types, and mechanistic details from these sources. The extracted information is then processed in to an active-site library (in SMARTS format). In Step 3, during the execution of ARplorer for comprehensive reaction pathway exploration, the system interacts with the active-site library at each iteration to identify the next possible reaction, which is subsequently filtered if it is not feasible.

z

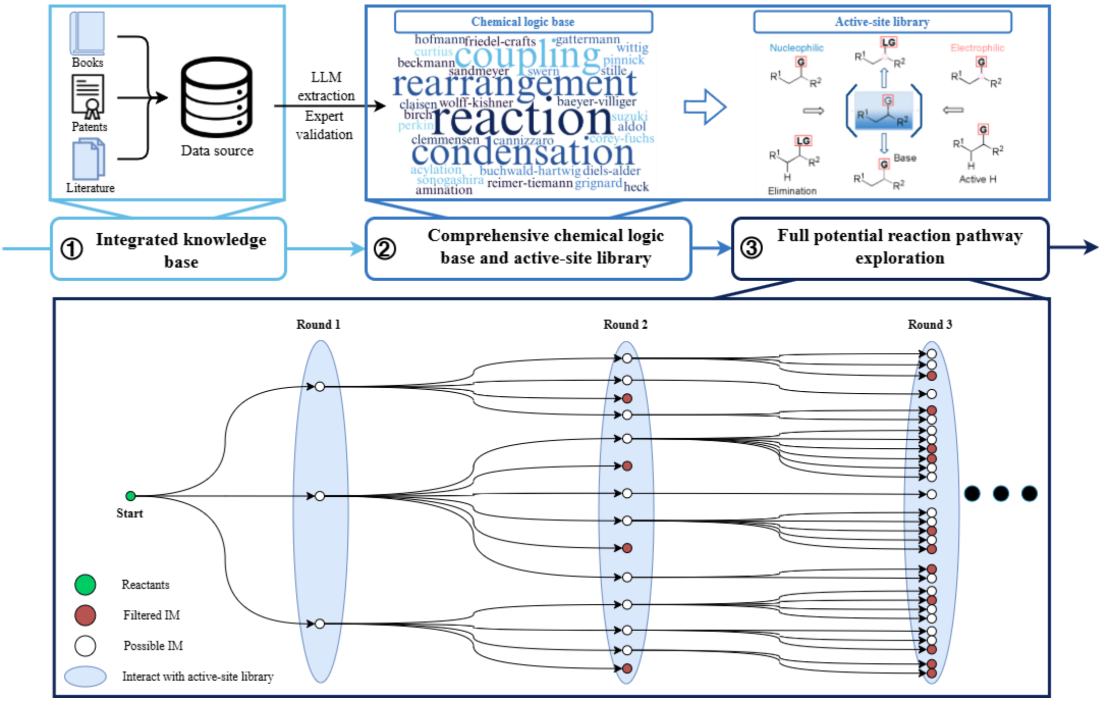

Figure S15 Overview of the ARplorer workflow

Design of Chain-of-through (COT) prompt

An effective strategy for designing Chain-of-Thought (COT) prompts to curate chemical knowledge bases involves structuring the prompts to mirror expert reasoning and reinforce scientific rigour. For instance, a well-designed COT prompt can sequentially guide the model through stages such as clarifying inputs[1], conducting structural analysis, decomposing functional groups, identifying reactive sites, and formulating reaction logic

based on explicit chemical principles. This approach not only improves the interpretability and transparency of the model’s outputs but also reduces the risk of errors and unsupported inferences during knowledge extraction. By embedding domain-specific reasoning steps directly into the prompt design, this method provides a robust framework for the reliable curation and expansion of chemical knowledge bases. An example of CoT prompt design is shown below.

“”””

1. Input Clarification
    - Prompt Example: “Verify the input reaction: Are all reactants specified? If not, list ambiguities.”
    - Purpose: Eliminates errors from misinterpreted or incomplete inputs.
2. Structural Analysis
    - Prompt Example: “Analyze the molecular structures: Identify bonds broken/formed and stereochemical constraints.”
    - Purpose: Grounds the model in explicit structural features.
3. Functional Group Decomposition
    - Prompt Example: “List all functional groups in reactants/products and their known reactivity patterns.”
    - *Anti-Hallucination Measure*: Limits proposals to documented group behaviors (e.g., “Carboxylates may undergo nucleophilic attack; avoid suggesting radical steps unless evidence exists”).
4. Reactive Site Identification
    - Prompt Example: “Rank potential reactive sites by electrophilicity/nucleophilicity, citing textbook rules (e.g., Markovnikov’s rule).”
    - Purpose: Prioritizes chemically logical sites over speculative ones.
5. Reaction Logic Formulation
    - Prompt Example: “Propose a mechanism only if all steps satisfy three criteria: (a) Orbital symmetry conservation, (b) Thermodynamic feasibility ($\Delta G < 0$), (c) No hypothetical intermediates.”
    - Validation: Requires self-checking before final output.

“”””

1. Liu, H., Yin, H., Luo, Z. & Wang, X. Integrating chemistry knowledge in large language models via prompt engineering. *Synth. Syst. Biotechnol.* **10**, 23–38 (2025).

DFT-optimized cartesian coordinates of all species involved in the cycloaddition reaction

A-IM1-TS1

C	0.4764	2.7820	-0.1171
C	1.8551	3.0314	-0.3492
C	2.5391	2.2875	0.5916
C	1.5621	1.7270	1.4094
O	0.3618	2.1272	1.0692
H	-0.3465	3.4262	-0.3831
H	2.2590	3.5842	-1.1736
H	3.5959	2.1428	0.6806
H	1.6605	1.0852	2.2668
C	-2.0061	1.5012	-2.2535
H	-1.5706	1.9435	-3.1291
H	-3.0638	1.3198	-2.2583
C	-1.2548	1.1438	-1.2123
C	0.2356	1.2946	-1.2788
H	0.5879	1.7262	-2.2245
O	0.9486	0.3586	-0.7806
O	-1.6736	0.6175	-0.0723
Si	-2.9303	-0.3698	0.4005
C	-2.7042	-0.3319	2.2809
H	-2.8089	0.6719	2.6826
H	-3.4395	-0.9620	2.7699
H	-1.7200	-0.6970	2.5553
C	-4.6164	0.3840	-0.0959
H	-4.8743	0.1793	-1.1316
H	-5.4117	-0.0306	0.5149
H	-4.6306	1.4617	0.0435
C	-2.6966	-2.0736	-0.4041
H	-1.6980	-2.4511	-0.1991
H	-3.4079	-2.8015	-0.0277
H	-2.8078	-2.0311	-1.4851

Al	1.5661	-1.1842	-0.1198
Cl	3.6189	-0.8319	-0.0180
Cl	0.7620	-1.5174	1.7727
Cl	0.9665	-2.5506	-1.5611

A-IM1

C	0.4203	2.7163	-0.2119
C	1.8215	2.9860	-0.3733
C	2.4609	2.2955	0.6282
C	1.4443	1.7597	1.4250
O	0.2633	2.1308	1.0181
H	-0.3686	3.4011	-0.4915
H	2.2592	3.5033	-1.2037
H	3.5130	2.1663	0.7792
H	1.5119	1.1546	2.3128
C	-2.0274	1.3458	-2.3430
H	-1.5815	1.7208	-3.2443
H	-3.0785	1.1285	-2.3580
C	-1.2999	1.1104	-1.2546
C	0.1965	1.3103	-1.2819
H	0.5454	1.6900	-2.2526
O	0.8997	0.3409	-0.7882
O	-1.7401	0.6734	-0.0841
Si	-2.9257	-0.3889	0.4098
C	-2.6862	-0.3075	2.2873
H	-2.8047	0.7028	2.6681
H	-3.4070	-0.9393	2.7950
H	-1.6939	-0.6508	2.5613
C	-4.6522	0.2624	-0.0923
H	-4.8970	0.0352	-1.1264
H	-5.4236	-0.1901	0.5220
H	-4.7257	1.3390	0.0365
C	-2.5986	-2.0887	-0.3691
H	-1.5779	-2.4040	-0.1666
H	-3.2628	-2.8515	0.0239
H	-2.7197	-2.0697	-1.4497
Al	1.5943	-1.1405	-0.1180
Cl	3.6372	-0.7057	-0.0281
Cl	0.8230	-1.4516	1.7989
Cl	1.0743	-2.6055	-1.4919

B

C	0.8826	2.8540	0.2282
C	2.1218	3.0796	-0.3328

C	2.9935	2.1710	0.2863
C	2.2313	1.4837	1.1958
O	0.9739	1.9285	1.1924
H	-0.0461	3.3814	0.1290
H	2.3556	3.7843	-1.1048
H	4.0342	2.0250	0.0866
H	2.4889	0.7157	1.8991
C	-1.7386	2.0854	-1.8957
H	-1.2717	2.8171	-2.5280
H	-2.8076	2.1020	-1.8166
C	-0.9940	1.1777	-1.2446
C	0.4588	1.1549	-1.4500
H	0.8598	1.8011	-2.2375
O	1.1746	0.2667	-0.9700
O	-1.4189	0.2072	-0.4518
Si	-2.8308	-0.1159	0.3932
C	-2.6468	0.7999	2.0524
H	-2.6472	1.8804	1.9271
H	-3.4531	0.5495	2.7338
H	-1.7116	0.5249	2.5313
C	-4.4004	0.4859	-0.5171
H	-4.3869	0.2216	-1.5711
H	-5.2764	0.0153	-0.0822
H	-4.5499	1.5594	-0.4375
C	-2.8564	-2.0043	0.4992
H	-1.9927	-2.3767	1.0391
H	-3.7453	-2.3524	1.0153
H	-2.8410	-2.4598	-0.4868
Al	1.2036	-1.3905	-0.1050
Cl	3.2699	-1.6311	-0.1016
Cl	0.4564	-1.3283	1.8271
Cl	0.2046	-2.5368	-1.5097

A-IM4

C	-0.4562	2.7952	0.1441
C	-1.8753	3.1470	0.2854
C	-2.5324	2.3335	-0.5736
C	-1.5007	1.6117	-1.2618
O	-0.3410	2.1981	-1.1055
H	0.3405	3.4901	0.3786
H	-2.2693	3.8007	1.0370
H	-3.5836	2.1680	-0.6926
H	-1.6154	0.8944	-2.0589
C	1.7256	1.1476	2.3596

H	1.2208	1.4949	3.2405
H	2.7535	0.8554	2.4556
C	1.0950	1.0465	1.1969
C	-0.3734	1.4038	1.0748
H	-0.8053	1.6474	2.0522
O	-1.0957	0.5126	0.3870
O	1.6069	0.6404	0.0406
Si	2.9147	-0.2912	-0.3930
C	2.7339	-0.2671	-2.2795
H	2.7497	0.7448	-2.6736
H	3.5396	-0.8194	-2.7510
H	1.7976	-0.7246	-2.5818
C	4.5402	0.5572	0.1496
H	4.7945	0.3466	1.1847
H	5.3718	0.2142	-0.4572
H	4.4807	1.6367	0.0390
C	2.7633	-2.0164	0.3881
H	1.8295	-2.4839	0.0867
H	3.5733	-2.6729	0.0872
H	2.7606	-1.9689	1.4743
Al	-1.5230	-1.2464	0.1003
Cl	-3.5895	-1.0566	-0.0844
Cl	-0.6578	-1.7834	-1.7107
Cl	-0.8132	-2.2015	1.7892

A-TS3

C	-0.8475	-2.8004	0.2287
C	-1.2500	-2.0061	1.2936
C	-2.5975	-1.7436	1.0861
C	-2.9255	-2.3503	-0.1283
O	-1.8994	-3.1227	-0.5379
H	0.0671	-3.3434	0.0844
H	-0.6189	-1.6432	2.0789
H	-3.2449	-1.1424	1.6896
H	-3.8929	-2.5673	-0.5426
C	-2.4147	-0.7581	-1.5269
H	-2.5013	-1.4185	-2.3719
H	-3.2443	-0.1020	-1.3326
C	-1.1642	-0.4073	-1.0730
C	-0.0246	-1.2117	-1.3923
H	-0.1492	-1.9900	-2.1529
O	1.1356	-0.9398	-1.0038
O	-0.9521	0.5366	-0.1671
Si	-1.5920	2.0459	0.1536

C	-0.6898	2.4993	1.7502
H	0.3821	2.5276	1.5818
H	-0.9966	3.4750	2.1120
H	-0.8722	1.7755	2.5388
C	-1.2235	3.1611	-1.3434
H	-1.7039	2.7999	-2.2503
H	-1.5604	4.1791	-1.1774
H	-0.1539	3.1944	-1.5296
C	-3.4858	1.9710	0.4268
H	-3.7825	1.1259	1.0410
H	-3.8185	2.8688	0.9375
H	-4.0422	1.9182	-0.5055
Al	2.3187	-0.0067	0.0269
Cl	4.0763	-1.0213	-0.3861
Cl	2.2583	1.9332	-0.6995
Cl	1.6504	-0.3313	1.9703

A-IM3

C	-0.4896	-2.6011	-0.0031
C	-0.9263	-1.9393	1.2700
C	-2.2460	-1.8067	1.1989
C	-2.6623	-2.3473	-0.1447
O	-1.6342	-3.2318	-0.4844
H	0.3539	-3.2845	0.0639
H	-0.2406	-1.6126	2.0274
H	-2.9250	-1.3704	1.9032
H	-3.6276	-2.8472	-0.2065
C	-2.5885	-1.1706	-1.2111
H	-2.6930	-1.6413	-2.1875
H	-3.3808	-0.4458	-1.0457
C	-1.2516	-0.5674	-1.0325
C	-0.0735	-1.4744	-1.1121
H	-0.0613	-2.0360	-2.0603
O	1.0975	-0.9120	-0.8366
O	-1.0765	0.5683	-0.5355
Si	-1.8158	1.9787	0.1244
C	-1.2707	1.8840	1.9320
H	-0.2395	1.5404	2.0004
H	-1.3245	2.8613	2.4010
H	-1.8808	1.2027	2.5204
C	-1.0696	3.3131	-0.9875
H	-1.4480	3.2670	-2.0066
H	-1.2814	4.3059	-0.6031
H	0.0123	3.1946	-1.0303

C	-3.7207	1.9035	-0.0455
H	-4.1685	1.0932	0.5252
H	-4.1478	2.8255	0.3369
H	-4.0486	1.8146	-1.0787
Al	2.2959	0.0598	-0.0528
Cl	4.1130	-0.8806	-0.3907
Cl	2.1850	1.9939	-0.8409
Cl	1.7827	0.0687	1.9993

A-IM3-TS1

C	-1.3232	-1.6629	0.5236
C	-2.0356	-0.8064	1.5359
C	-3.3000	-0.7166	1.1493
C	-3.4288	-1.5129	-0.1292
O	-2.3503	-2.4090	-0.0715
H	-0.5322	-2.3092	0.9007
H	-1.5424	-0.3637	2.3773
H	-4.1106	-0.1729	1.5920
H	-4.3603	-2.0623	-0.2624
C	-3.1680	-0.5652	-1.3567
H	-3.0925	-1.1995	-2.2393
H	-3.9624	0.1698	-1.4841
C	-1.8785	0.1037	-1.0554
C	-0.7320	-0.7230	-0.6146
H	-0.4021	-1.4122	-1.4052
O	0.2871	0.1008	-0.2172
O	-1.7847	1.3263	-0.9430
Si	-0.3351	2.1116	0.0600
C	0.5449	1.9702	1.7519
H	1.3910	1.2901	1.7270
H	0.9176	2.9496	2.0329
H	-0.1197	1.6431	2.5470
C	0.6531	2.7960	-1.4074
H	0.0103	3.0318	-2.2510
H	1.1596	3.7084	-1.1148
H	1.4119	2.0959	-1.7423
C	-1.6921	3.3938	0.5453
H	-2.4329	2.9821	1.2243
H	-1.2340	4.2397	1.0433
H	-2.2209	3.7675	-0.3237
Al	2.0294	-0.6208	-0.0148
Cl	2.0592	-1.9768	-1.6064
Cl	3.4415	0.8692	-0.1754
Cl	1.9331	-1.5780	1.8364

A-IM5

C	-1.5026	-1.3435	0.5799
C	-2.2339	-0.4913	1.5867
C	-3.4872	-0.3902	1.1722
C	-3.6009	-1.1848	-0.1112
O	-2.5278	-2.0993	-0.0220
H	-0.7311	-2.0032	0.9792
H	-1.7579	-0.0683	2.4490
H	-4.3037	0.1566	1.6014
H	-4.5328	-1.7370	-0.2366
C	-3.3298	-0.2545	-1.3193
H	-3.2899	-0.8704	-2.2200
H	-4.1056	0.5024	-1.4408
C	-1.9966	0.4220	-1.1301
C	-0.9070	-0.4372	-0.5434
H	-0.5961	-1.1338	-1.3366
O	0.2188	0.2988	-0.1391
O	-1.7935	1.5798	-1.4025
Si	0.2202	2.0201	0.1715
C	1.5046	2.0883	1.5773
H	2.4429	1.6204	1.2973
H	1.7214	3.1208	1.8317
H	1.1541	1.5962	2.4806
C	0.8791	2.8933	-1.3832
H	0.1250	2.9998	-2.1557
H	1.2305	3.8862	-1.1234
H	1.7201	2.3484	-1.8027
C	-1.3394	2.7878	0.9648
H	-2.0015	2.0578	1.4170
H	-1.0395	3.4879	1.7371
H	-1.9154	3.3362	0.2290
Al	1.9481	-0.8524	-0.0182
Cl	1.4897	-2.4529	-1.2578
Cl	3.4526	0.2867	-0.8316
Cl	1.9032	-1.3205	1.9880

A-TS2

C	-0.1915	2.0018	1.0985
C	-1.3877	2.4700	0.5611
C	-1.2393	2.3907	-0.8219
C	0.0488	1.9390	-1.0396
O	0.6973	1.8098	0.1159
H	0.1939	2.0516	2.0996

H	-2.2567	2.7666	1.1105
H	-1.9694	2.6176	-1.5707
H	0.6192	1.8322	-1.9424
C	-0.7963	-0.1437	1.2296
H	-1.6149	-0.0337	1.9183
H	0.1793	-0.3577	1.6359
C	-1.0459	-0.4332	-0.0861
C	0.0144	-0.6470	-1.0131
H	-0.2932	-0.8011	-2.0541
O	1.2256	-0.7644	-0.7211
O	-2.2705	-0.3928	-0.6404
Si	-3.7909	-0.7423	-0.0710
C	-4.4891	0.6714	1.0028
H	-4.4468	1.6212	0.4773
H	-5.5302	0.4857	1.2467
H	-3.9571	0.7888	1.9431
C	-4.7468	-0.8855	-1.7023
H	-4.6649	0.0224	-2.2915
H	-4.3778	-1.7046	-2.3112
H	-5.8003	-1.0628	-1.5162
C	-3.7518	-2.3684	0.9186
H	-3.1543	-2.2797	1.8221
H	-4.7474	-2.6793	1.2173
H	-3.3229	-3.1724	0.3268
Al	2.8795	-0.4491	-0.0243
Cl	3.5347	1.0894	-1.2510
Cl	2.6577	0.0961	1.9810
Cl	3.7531	-2.3064	-0.2431

A-IM2

C	0.2892	-1.6755	1.1209
C	1.4418	-2.3876	0.5331
C	1.2625	-2.3291	-0.8083
C	-0.0195	-1.7404	-1.0101
O	-0.6870	-1.6834	0.1257
H	-0.1184	-1.9719	2.0825
H	2.2853	-2.7438	1.0876
H	1.9278	-2.6399	-1.5882
H	-0.5933	-1.6940	-1.9201
C	0.7435	-0.1166	1.1889
H	1.6132	-0.0414	1.8335
H	-0.1235	0.3942	1.6100
C	1.0199	0.3209	-0.1816
C	-0.0061	0.6500	-1.0627

H	0.2860	0.9061	-2.0851
O	-1.2287	0.7949	-0.7349
O	2.2668	0.3051	-0.6620
Si	3.7602	0.7406	-0.0684
C	4.4920	-0.6264	1.0437
H	4.5037	-1.5860	0.5342
H	5.5188	-0.3906	1.3042
H	3.9492	-0.7517	1.9772
C	4.7444	0.8965	-1.6808
H	4.7175	-0.0248	-2.2544
H	4.3511	1.6863	-2.3131
H	5.7845	1.1262	-1.4769
C	3.6053	2.3753	0.8932
H	3.0270	2.2597	1.8063
H	4.5773	2.7677	1.1731
H	3.1087	3.1319	0.2916
Al	-2.8321	0.4196	-0.0383
Cl	-3.4899	-1.2001	-1.1725
Cl	-2.5600	-0.0289	2.0006
Cl	-3.8845	2.1828	-0.2784

A-IM2-TS1

C	0.3611	-1.6355	1.1540
C	1.4783	-2.3392	0.4681
C	1.1973	-2.2783	-0.8451
C	-0.1100	-1.6537	-0.9550
O	-0.6919	-1.6912	0.2494
H	0.0481	-1.9516	2.1449
H	2.3516	-2.7174	0.9582
H	1.7909	-2.5998	-1.6772
H	-0.7850	-1.7854	-1.7889
C	0.7641	-0.0614	1.2101
H	1.6313	0.0585	1.8521
H	-0.1286	0.4195	1.6188
C	1.0261	0.3236	-0.1699
C	-0.0321	0.3389	-1.1192
H	0.2827	0.4974	-2.1573
O	-1.2406	0.6756	-0.8226
O	2.2477	0.3171	-0.6528
Si	3.7755	0.7226	-0.0826
C	4.5076	-0.6478	1.0215
H	4.5450	-1.6000	0.4997
H	5.5262	-0.3974	1.3005
H	3.9522	-0.7943	1.9443

C	4.7116	0.8564	-1.7250
H	4.6714	-0.0736	-2.2845
H	4.2969	1.6355	-2.3574
H	5.7567	1.0923	-1.5564
C	3.6286	2.3667	0.8619
H	3.0977	2.2557	1.8041
H	4.6054	2.7812	1.0886
H	3.0894	3.1043	0.2733
Al	-2.8217	0.4238	-0.0534
Cl	-3.5858	-1.3110	-0.9257
Cl	-2.4846	0.2263	2.0224
Cl	-3.8487	2.1682	-0.4739

A-TS1

C	0.4764	2.7820	-0.1171
C	1.8551	3.0314	-0.3492
C	2.5391	2.2875	0.5916
C	1.5621	1.7270	1.4094
O	0.3618	2.1272	1.0692
H	-0.3465	3.4262	-0.3831
H	2.2590	3.5842	-1.1736
H	3.5959	2.1428	0.6806
H	1.6605	1.0852	2.2668
C	-2.0061	1.5012	-2.2535
H	-1.5706	1.9435	-3.1291
H	-3.0638	1.3198	-2.2583
C	-1.2548	1.1438	-1.2123
C	0.2356	1.2946	-1.2788
H	0.5879	1.7262	-2.2245
O	0.9486	0.3586	-0.7806
O	-1.6736	0.6175	-0.0723
Si	-2.9303	-0.3698	0.4005
C	-2.7042	-0.3319	2.2809
H	-2.8089	0.6719	2.6826
H	-3.4395	-0.9620	2.7699
H	-1.7200	-0.6970	2.5553
C	-4.6164	0.3840	-0.0959
H	-4.8743	0.1793	-1.1316
H	-5.4117	-0.0306	0.5149
H	-4.6306	1.4617	0.0435
C	-2.6966	-2.0736	-0.4041
H	-1.6980	-2.4511	-0.1991
H	-3.4079	-2.8015	-0.0277
H	-2.8078	-2.0311	-1.4851

Al	1.5661	-1.1842	-0.1198
Cl	3.6189	-0.8319	-0.0180
Cl	0.7620	-1.5174	1.7727
Cl	0.9665	-2.5506	-1.5611

A-IM1

C	0.4203	2.7163	-0.2119
C	1.8215	2.9860	-0.3733
C	2.4609	2.2955	0.6282
C	1.4443	1.7597	1.4250
O	0.2633	2.1308	1.0181
H	-0.3686	3.4011	-0.4915
H	2.2592	3.5033	-1.2037
H	3.5130	2.1663	0.7792
H	1.5119	1.1546	2.3128
C	-2.0274	1.3458	-2.3430
H	-1.5815	1.7208	-3.2443
H	-3.0785	1.1285	-2.3580
C	-1.2999	1.1104	-1.2546
C	0.1965	1.3103	-1.2819
H	0.5454	1.6900	-2.2526
O	0.8997	0.3409	-0.7882
O	-1.7401	0.6734	-0.0841
Si	-2.9257	-0.3889	0.4098
C	-2.6862	-0.3075	2.2873
H	-2.8047	0.7028	2.6681
H	-3.4070	-0.9393	2.7950
H	-1.6939	-0.6508	2.5613
C	-4.6522	0.2624	-0.0923
H	-4.8970	0.0352	-1.1264
H	-5.4236	-0.1901	0.5220
H	-4.7257	1.3390	0.0365
C	-2.5986	-2.0887	-0.3691
H	-1.5779	-2.4040	-0.1666
H	-3.2628	-2.8515	0.0239
H	-2.7197	-2.0697	-1.4497
Al	1.5943	-1.1405	-0.1180
Cl	3.6372	-0.7057	-0.0281
Cl	0.8230	-1.4516	1.7989
Cl	1.0743	-2.6055	-1.4919

A-IM1-TS2

C	-0.0394	2.5545	-0.5783
C	0.3899	2.2494	0.8147

C	-0.7039	1.9062	1.5081
C	-1.8156	2.0178	0.5757
O	-1.4638	2.7330	-0.4643
H	0.4113	3.4291	-1.0414
H	1.4193	2.2291	1.1203
H	-0.7728	1.5489	2.5140
H	-2.8627	2.0264	0.8405
C	-1.8150	0.1124	-2.5834
H	-1.3464	0.2566	-3.5383
H	-2.7542	-0.4096	-2.5626
C	-1.2317	0.5399	-1.4763
C	0.1217	1.2546	-1.4827
H	0.3230	1.5905	-2.5120
O	1.0901	0.4192	-1.0546
O	-1.7744	0.3398	-0.2318
Si	-2.4016	-1.1706	0.3594
C	-2.2925	-0.9795	2.2393
H	-2.8268	-0.1110	2.6151
H	-2.7215	-1.8538	2.7181
H	-1.2535	-0.9151	2.5525
C	-4.2287	-1.2246	-0.2067
H	-4.3442	-1.6373	-1.2052
H	-4.8086	-1.8537	0.4607
H	-4.6899	-0.2399	-0.2056
C	-1.3184	-2.5175	-0.4054
H	-0.2661	-2.2453	-0.3343
H	-1.4446	-3.4589	0.1195
H	-1.5408	-2.6908	-1.4552
Al	2.1866	-0.4417	-0.0443
Cl	3.7701	0.8711	0.3570
Cl	1.1927	-0.8373	1.7910
Cl	2.6527	-2.2074	-1.0283

A-IM9

C	0.1807	2.7057	0.6315
C	-0.2456	2.5022	-0.7994
C	0.7488	1.8885	-1.4216
C	1.8420	1.7102	-0.3970
O	1.6154	2.6707	0.5483
H	-0.1275	3.6405	1.0937
H	-1.2197	2.7558	-1.1654
H	0.8043	1.5248	-2.4254
H	2.8757	1.7286	-0.7420
C	1.2835	-0.2158	2.5409

H	0.7009	-0.1375	3.4391
H	2.0944	-0.9203	2.5399
C	0.9597	0.5043	1.4845
C	-0.2404	1.4431	1.4530
H	-0.4721	1.7338	2.4892
O	-1.2955	0.7805	0.9018
O	1.6652	0.3866	0.2806
Si	2.3742	-1.1705	-0.3771
C	2.3752	-0.9394	-2.2546
H	2.9081	-0.0521	-2.5868
H	2.8545	-1.7951	-2.7208
H	1.3529	-0.8970	-2.6256
C	4.1487	-1.0651	0.3268
H	4.1831	-1.1133	1.4121
H	4.7463	-1.8901	-0.0478
H	4.6522	-0.1471	0.0314
C	1.3004	-2.5612	0.3051
H	0.3025	-2.2171	0.5764
H	1.1645	-3.3210	-0.4587
H	1.7352	-3.0483	1.1739
Al	-2.1773	-0.3726	-0.0082
Cl	-3.9623	0.5200	-0.5677
Cl	-0.9977	-0.8188	-1.7281
Cl	-2.3041	-2.1217	1.1395

A-IM1-TS3

C	0.1633	2.0627	0.1195
C	0.9981	1.3577	-0.8890
C	2.0833	2.2424	-1.1549
C	1.9612	3.2872	-0.2953
O	0.8720	3.2435	0.4561
H	-0.8439	2.3147	-0.2355
H	0.5562	0.6647	-1.5875
H	2.8729	2.0802	-1.8586
H	2.5952	4.1474	-0.1672
C	1.8821	0.0722	2.5879
H	1.4572	0.3736	3.5261
H	2.7857	-0.5079	2.6116
C	1.2756	0.3798	1.4549
C	-0.0431	1.1197	1.3520
H	-0.2429	1.7295	2.2449
O	-1.0436	0.2421	1.1127
O	1.7182	0.0436	0.2144
Si	2.1537	-1.5710	-0.2793

C	2.1132	-1.4810	-2.1698
H	2.6709	-0.6359	-2.5636
H	2.5526	-2.3818	-2.5861
H	1.0921	-1.4203	-2.5366
C	3.9527	-1.7654	0.3390
H	4.0043	-2.1240	1.3631
H	4.4779	-2.4903	-0.2742
H	4.5065	-0.8317	0.2862
C	0.9327	-2.7601	0.5411
H	-0.0915	-2.4407	0.3532
H	1.0378	-3.7683	0.1538
H	1.0644	-2.8038	1.6198
Al	-2.2891	-0.2799	0.0257
Cl	-3.3528	1.4712	-0.4593
Cl	-1.3380	-0.8854	-1.7826
Cl	-3.2609	-1.8561	0.9271

A-IM10

C	0.6096	2.2289	-0.0228
C	1.4128	1.1224	-0.7198
C	2.7749	1.7304	-0.7926
C	2.7495	2.8793	-0.1185
O	1.5461	3.2257	0.3734
H	-0.1507	2.6863	-0.6600
H	0.9721	0.7618	-1.6478
H	3.6152	1.2755	-1.2727
H	3.5412	3.5811	0.0732
C	1.0158	-0.2966	2.5547
H	0.5059	0.0414	3.4369
H	1.6198	-1.1799	2.6423
C	0.8361	0.3470	1.4201
C	-0.0727	1.5277	1.1700
H	-0.1342	2.2004	2.0365
O	-1.3057	1.0683	0.8138
O	1.4067	-0.0188	0.2068
Si	1.9676	-1.6853	-0.2751
C	2.0024	-1.6334	-2.1649
H	2.6746	-0.8809	-2.5686
H	2.3306	-2.5951	-2.5474
H	1.0033	-1.4495	-2.5564
C	3.7234	-1.6848	0.4773
H	3.7265	-1.4915	1.5467
H	4.1943	-2.6506	0.3241
H	4.3659	-0.9387	0.0155

C	0.7387	-2.8939	0.4877
H	-0.2395	-2.4360	0.6456
H	0.5838	-3.7342	-0.1828
H	1.0713	-3.3004	1.4390
Al	-2.3504	0.0156	-0.0512
Cl	-4.0942	1.0183	-0.5162
Cl	-1.2510	-0.5012	-1.8140
Cl	-2.5721	-1.7326	1.0887

A-IM2-TS3

C	-0.0089	2.1631	0.9471
C	-1.1451	2.9400	0.3866
C	-1.2383	2.5623	-0.9027
C	-0.1167	1.6854	-1.1351
O	0.7781	1.8365	-0.1688
H	0.5915	2.5660	1.7559
H	-1.8032	3.5586	0.9620
H	-1.9872	2.8051	-1.6287
H	0.2530	1.3661	-2.0959
C	-0.6509	0.7467	1.3276
H	-1.4878	0.8926	2.0046
H	0.1497	0.1754	1.7973
C	-1.0869	0.1206	0.0558
C	-0.2316	-0.6833	-0.7026
H	-0.6660	-1.1556	-1.5901
O	0.9878	-0.8840	-0.4509
O	-2.3920	0.1871	-0.2637
Si	-3.6292	-0.9030	-0.0239
C	-3.8749	-1.1685	1.8467
H	-4.0889	-0.2341	2.3591
H	-4.7021	-1.8415	2.0471
H	-2.9886	-1.5999	2.3055
C	-5.0615	0.0379	-0.8351
H	-5.2138	1.0082	-0.3733
H	-4.8830	0.2025	-1.8930
H	-5.9864	-0.5200	-0.7414
C	-3.2423	-2.5608	-0.8735
H	-2.3413	-3.0139	-0.4676
H	-4.0499	-3.2729	-0.7393
H	-3.0958	-2.4384	-1.9433
Al	2.7078	-0.5302	-0.0231
Cl	3.3195	0.6532	-1.6187
Cl	2.7423	0.4158	1.8459
Cl	3.5180	-2.4303	0.0818

A-IM8

C	-0.0711	2.1481	0.9203
C	-1.1992	3.0401	0.4595
C	-1.4474	2.6718	-0.7914
C	-0.4396	1.5914	-1.0816
O	0.6457	1.9063	-0.2817
H	0.5869	2.4967	1.7084
H	-1.6998	3.7574	1.0778
H	-2.2058	2.9861	-1.4781
H	-0.1689	1.3910	-2.1128
C	-0.7380	0.7626	1.1685
H	-1.6455	0.8377	1.7597
H	-0.0258	0.0916	1.6466
C	-1.0539	0.3076	-0.2654
C	-0.1947	-0.7742	-0.7723
H	-0.6558	-1.4099	-1.5420
O	0.9906	-0.9088	-0.4986
O	-2.3777	0.1299	-0.5824
Si	-3.4652	-0.9642	0.0285
C	-4.1650	-0.3637	1.6990
H	-4.3616	0.7053	1.6835
H	-5.1035	-0.8598	1.9247
H	-3.4917	-0.5666	2.5282
C	-4.7893	-0.9445	-1.3285
H	-5.1852	0.0550	-1.4760
H	-4.3970	-1.2847	-2.2816
H	-5.6183	-1.5910	-1.0632
C	-2.6455	-2.6690	0.2609
H	-1.6903	-2.5802	0.7743
H	-3.2674	-3.3342	0.8508
H	-2.4609	-3.1633	-0.6887
Al	2.7461	-0.4569	0.0068
Cl	3.2453	0.5025	-1.7492
Cl	2.7692	0.6077	1.7811
Cl	3.4325	-2.3887	0.2910

A-IM3-TS2

C	-1.63851400	-3.00754600	-0.01910800
C	-0.93747200	-2.57533000	-1.13441600
C	-0.07951900	-1.58624600	-0.69616800
C	-0.28793200	-1.43903800	0.76033300
O	-1.31287100	-2.37612600	1.07460900
H	-2.36749000	-3.79701900	0.06276800

H	-1.04667000	-2.95009900	-2.13207800
H	0.74624600	-1.15626500	-1.22876000
H	0.66051600	-1.69723400	1.30720000
C	-0.68325700	-0.00820800	1.11422300
H	-1.59220800	-0.00666400	1.71159100
H	0.11584000	0.48578800	1.66854300
C	-0.90639100	0.73128100	-0.19974900
C	0.14233300	1.24003400	-0.88602900
H	-0.03657300	1.75980100	-1.82373900
O	1.39235200	1.07595000	-0.52621500
O	-2.15517000	0.94188700	-0.65166700
Si	-3.69344100	0.91408300	-0.06430800
C	-3.86209200	1.99115200	1.50025400
H	-4.90155100	2.22506000	1.70613100
H	-3.46031400	1.50764200	2.38696000
H	-3.33406900	2.93261500	1.37683000
C	-4.65597100	1.61806700	-1.53751000
H	-4.49326500	1.03207100	-2.43651800
H	-5.72163600	1.62434100	-1.33578500
H	-4.35562200	2.63797100	-1.75454600
C	-4.25681200	-0.87259000	0.31979800
H	-3.80831200	-1.26990800	1.22634700
H	-5.33295900	-0.91451100	0.45253700
H	-4.00420400	-1.54447600	-0.49725700
Al	2.95267500	0.46774700	-0.09378300
Cl	2.61547600	-0.88041900	1.56770900
Cl	3.55191200	-0.73090400	-1.69523300
Cl	4.17702100	1.99778600	0.53491900

A-IM6-TS

C	2.42469700	-2.77459400	-0.02281500
C	2.02529700	-1.71154400	1.03764400
C	0.62908000	-1.90626200	1.27509600
C	0.19780800	-2.48107600	0.03787500
O	1.18541900	-3.33336700	-0.43710600
H	3.11575100	-3.57773300	0.22659500
H	2.71988900	-1.39994000	1.80139600
H	-0.01208700	-1.35106900	1.94280400
H	-0.80576500	-2.84979100	-0.13577100
C	2.87318100	-1.68321700	-0.99506900
H	2.66547000	-1.85424700	-2.05006400
H	3.91098400	-1.39206000	-0.85370700
C	1.91517200	-0.71715500	-0.28041600
C	0.43286000	-1.01654300	-0.77273400

H	0.43287000	-1.33177700	-1.83161300
O	-0.45707300	-0.08879300	-0.53167400
O	2.29400800	0.54011700	-0.17715900
Si	1.65156500	2.08315300	0.00969300
C	0.56168000	2.24506800	1.55472500
H	0.97456400	1.70517800	2.40346400
H	0.46365900	3.28631800	1.84519800
H	-0.44000600	1.86362300	1.37309000
C	3.30035300	3.00006800	0.27424300
H	3.98415100	2.84478600	-0.55415600
H	3.13158800	4.06740400	0.36252200
H	3.80476900	2.67488200	1.17871700
C	0.84896100	2.63718500	-1.62136200
H	-0.11732400	2.16638800	-1.77019400
H	0.68977500	3.71056700	-1.61930800
H	1.47790800	2.39506800	-2.47433300
Al	-2.16484800	0.15306700	-0.06728000
Cl	-3.19702300	-1.38005900	-1.03948400
Cl	-2.13620200	-0.26794200	2.00458600
Cl	-2.69080200	2.08289300	-0.54518000

A-IM6

C	-2.93075400	-3.08098800	0.24952700
C	-1.78188900	-2.57854400	0.69160900
C	-1.82816300	-1.09222400	0.55353200
C	-3.19493800	-0.89514600	-0.13260200
O	-3.82452000	-2.16337700	-0.20999700
H	-3.27085000	-4.09962200	0.21787300
H	-0.96124200	-3.11533900	1.12113700
H	-1.57586000	-0.55166400	1.46184500
H	-3.89953400	-0.18046500	0.29227900
C	-2.48809800	-0.44509600	-1.42173500
H	-2.55093100	-1.19256700	-2.20976000
H	-2.78036000	0.52415600	-1.80857400
C	-1.11561800	-0.42490600	-0.70268400
C	-0.08079000	-1.35124600	-1.21378400
H	-0.43314900	-2.24161400	-1.75645100
O	1.10986900	-1.21121200	-1.00134400
O	-0.55289100	0.79736600	-0.45719700
Si	-1.10067300	2.22493700	0.18983400
C	-1.68626000	2.05019600	1.99815800
H	-0.94531200	1.52960500	2.59856400
H	-1.82513100	3.03041900	2.44367300
H	-2.63209500	1.52202900	2.09391600

C	0.44239400	3.30872900	0.04837000
H	0.80516800	3.36153000	-0.97284400
H	0.23515700	4.31977700	0.38253500
H	1.24965500	2.91524500	0.65774100
C	-2.54193500	2.95692700	-0.83288700
H	-3.48696100	2.44667500	-0.66487600
H	-2.69395500	3.99627000	-0.56033700
H	-2.33237900	2.93668000	-1.89901200
Al	2.44051500	-0.31795900	0.04254500
Cl	3.73628300	-1.92872600	0.17532200
Cl	2.97537000	1.17446200	-1.26122600
Cl	1.53288600	0.03160600	1.86912800

A-IM7

C	2.95030200	-1.54467100	-0.75885200
C	1.49153700	-1.71736500	-0.25196000
C	1.70140400	-1.75604700	1.23704500
C	3.00090000	-1.74272700	1.48609700
O	3.79699900	-1.68474500	0.37304600
H	3.26005400	-2.28246100	-1.50179700
H	0.98990300	-2.62128500	-0.60107700
H	0.89181200	-1.82133200	1.93324700
H	3.53494300	-1.78797600	2.41632100
C	3.05994100	-0.09837700	-1.30316200
H	3.13016200	-0.07777800	-2.39214600
H	3.93833000	0.40679400	-0.89909300
C	1.79658000	0.54720700	-0.86450500
C	0.72213400	-0.46095900	-0.73273800
H	0.38253500	-0.67624300	-1.77245800
O	-0.32043100	-0.00603100	0.00730800
O	1.66788200	1.73081000	-0.55095700
Si	0.20758100	2.50402400	0.24665400
C	0.04745100	1.90222400	2.03974900
H	-0.85065500	1.30398800	2.16028300
H	0.89146700	1.28329000	2.33510600
H	-0.01056900	2.74540900	2.72181400
C	1.09031400	4.19910900	0.31144300
H	1.37347300	4.55377100	-0.67505700
H	0.43958200	4.94833200	0.74851000
H	1.99321000	4.16601100	0.91405200
C	-1.15525000	2.66060500	-1.06200400
H	-1.75824900	1.76097200	-1.14382600
H	-1.82088900	3.47658900	-0.79837500
H	-0.73867300	2.87950200	-2.04351300

Al	-1.94707400	-0.80496900	-0.00724100
Cl	-2.19196200	-1.05204800	-2.09243100
Cl	-1.75754900	-2.66400500	0.91628500
Cl	-3.26390600	0.47466500	0.94068300

A-IM7-TS

C	-2.99654000	-1.50111300	0.64151400
C	-1.55707200	-1.62547300	0.06007000
C	-1.80118000	-1.33375300	-1.39452000
C	-3.10002600	-1.16718200	-1.58728000
O	-3.86728700	-1.29230300	-0.46278600
H	-3.34146300	-2.39064000	1.17370600
H	-1.10292200	-2.60804000	0.19441100
H	-1.01494400	-1.31336300	-2.11939900
H	-3.65266800	-0.96718200	-2.48593900
C	-3.01161000	-0.24871300	1.55188900
H	-2.98626500	-0.52149900	2.60752000
H	-3.89618400	0.36186000	1.37114500
C	-1.75720200	0.45140800	1.17263700
C	-0.72148800	-0.53872700	0.78070200
H	-0.31908500	-0.98399500	1.71085100
O	0.28714300	0.09976200	0.10453500
O	-1.57001700	1.64639500	1.02954100
Si	-0.11303400	2.18822800	-0.22363200
C	-1.20914100	3.75943300	-0.41444000
H	-1.48054000	4.18758600	0.54381400
H	-0.66694300	4.51443300	-0.97194000
H	-2.12994200	3.56111400	-0.95376700
C	1.27307400	2.67948200	0.97573800
H	0.87422200	3.14316500	1.87448800
H	1.88428000	1.83186000	1.27190700
H	1.92644900	3.40109300	0.49825100
C	0.10786200	1.71398300	-2.06103000
H	-0.79310100	1.26575000	-2.47393900
H	0.32856900	2.60134100	-2.64485800
H	0.92107300	1.00863300	-2.19950100
Al	1.95401900	-0.76778700	0.00931600
Cl	2.24245100	-1.18022200	2.04665800
Cl	1.55542900	-2.49957800	-1.07345500
Cl	3.34746800	0.47315300	-0.86196800

A-IM11

C	-3.07883100	-1.41580700	0.19638000
C	-1.70505500	-1.28099100	-0.51492100

C	-2.05864300	-0.41455200	-1.68992900
C	-3.35927700	-0.15859600	-1.65601600
O	-4.02505400	-0.70647700	-0.60448900
H	-3.42936800	-2.44900900	0.26945700
H	-1.29839800	-2.23669400	-0.85142600
H	-1.34943900	-0.10917400	-2.42962200
H	-3.97365500	0.40616600	-2.33306100
C	-2.94767300	-0.76450800	1.58083600
H	-2.92434900	-1.50986200	2.37702700
H	-3.78092900	-0.09004100	1.77834500
C	-1.63996400	-0.01737200	1.57470700
C	-0.75339100	-0.64673900	0.51891400
H	-0.23260100	-1.46854300	1.03945500
O	0.24623800	0.20650500	0.02229100
O	-1.32279200	0.90633200	2.26973300
Si	0.07057500	1.91227800	-0.19762100
C	-1.70571900	2.58039400	-0.05099400
H	-1.93850000	2.85683600	0.97101100
H	-1.78889300	3.47502100	-0.65928400
H	-2.46373600	1.88331000	-0.39080600
C	1.21624200	2.67240100	1.11074300
H	0.82927100	2.50637200	2.11304700
H	2.20700900	2.22693400	1.06075900
H	1.32422800	3.74253500	0.96711500
C	0.63642300	2.12295700	-2.00650400
H	-0.20034800	2.23387300	-2.69104500
H	1.25029500	3.01152400	-2.10630600
H	1.23490800	1.27840000	-2.33767300
Al	2.03782000	-0.76414000	-0.03188600
Cl	2.22487900	-1.16830300	1.98723600
Cl	1.47418400	-2.33559400	-1.25296000
Cl	3.49170200	0.45038400	-0.82106200

DFT-optimized cartesian coordinates of all species involved in the asymmetric Mannich-type reaction

B-TS-SR

N	-1.0929	-1.3631	0.9490
O	0.2833	-1.1968	0.7591
C	-1.7362	-1.3750	-0.3699
C	-1.5659	-0.3510	1.8591

C	-2.0104	-2.8355	-0.7319
H	-1.0052	-0.9638	-1.0883
H	-1.5191	0.6692	1.4331
H	-2.5862	-0.5745	2.1729
H	-0.9172	-0.3830	2.7323
H	-1.0948	-3.4025	-0.5964
H	-2.7701	-3.2463	-0.0712
H	-2.3419	-2.9310	-1.7638
C	-2.8105	0.9370	-0.5499
C	-3.0307	-0.5528	-0.5065
O	-1.8980	1.4563	-1.1589
H	-3.7559	-0.8223	0.2601
H	-3.4559	-0.8161	-1.4800
Li	1.3324	-0.0509	0.2125
O	1.7411	1.8193	0.1433
O	3.0289	-0.8987	-0.2457
C	2.9083	2.3359	0.7213
H	3.5368	2.8309	-0.0295
H	2.6724	3.0603	1.5098
H	3.4526	1.5026	1.1539
C	0.9447	2.8195	-0.4444
H	1.4780	3.3104	-1.2667
H	0.0488	2.3392	-0.8277
H	0.6624	3.5777	0.2953
C	4.2054	-0.5830	-0.9374
H	4.3221	-1.2121	-1.8281
H	4.1379	0.4569	-1.2452
H	5.0894	-0.7146	-0.2995
C	2.9867	-2.2455	0.1659
H	3.7804	-2.4579	0.8911
H	2.0080	-2.3781	0.6206
H	3.0933	-2.9182	-0.6916
C	-3.8843	1.7605	0.1261
H	-4.8398	1.5585	-0.3556
H	-3.9707	1.4855	1.1740
H	-3.6574	2.8206	0.0431

B-SR

N	-1.7770	-0.4984	0.8457
O	-0.6755	0.3745	0.6753
C	-2.3337	-0.6155	-0.5057
C	-2.6917	0.0479	1.8376
C	-3.7217	-1.2299	-0.5583
H	-1.6285	-1.2616	-1.0464

H	-3.2411	0.9358	1.5060
H	-3.4007	-0.7294	2.1176
H	-2.1003	0.3112	2.7137
H	-3.7342	-2.1828	-0.0334
H	-4.4643	-0.5716	-0.1137
H	-3.9975	-1.4010	-1.5961
C	-0.9000	1.3293	-0.4972
C	-2.2282	0.7906	-1.0965
O	0.1413	1.2233	-1.2848
H	-3.0814	1.4069	-0.8104
H	-2.1434	0.7728	-2.1793
Li	1.0206	0.0651	-0.3685
O	2.7426	0.7518	0.2139
O	1.3722	-1.8287	-0.2883
C	3.7298	0.3354	1.1158
H	4.7104	0.2664	0.6268
H	3.8089	1.0254	1.9643
H	3.4400	-0.6449	1.4823
C	2.9874	2.0344	-0.3099
H	3.9124	2.0478	-0.8982
H	2.1333	2.2562	-0.9434
H	3.0611	2.7772	0.4921
C	2.4070	-2.4500	-1.0004
H	2.0196	-3.2489	-1.6441
H	2.8742	-1.6872	-1.6157
H	3.1537	-2.8789	-0.3204
C	0.6794	-2.7188	0.5513
H	1.3455	-3.1315	1.3182
H	-0.1197	-2.1534	1.0221
H	0.2500	-3.5466	-0.0253
C	-1.0628	2.7346	0.1126
H	-1.2237	3.4422	-0.6960
H	-1.8934	2.7811	0.8117
H	-0.1418	2.9928	0.6286

B-S3

N	-0.8827	-1.3385	0.8781
O	0.5134	-1.2575	0.7560
C	-1.4323	-1.1784	-0.4656
C	-1.3073	-0.3018	1.7935
C	-1.0999	-2.4440	-1.2534
H	-0.9277	-0.3163	-0.9574
H	-1.1101	0.7128	1.4020
H	-2.3656	-0.4067	2.0302

H	-0.7321	-0.4331	2.7077
H	-0.0397	-2.6464	-1.1330
H	-1.6611	-3.2841	-0.8523
H	-1.3311	-2.3203	-2.3089
C	-3.3287	0.5226	-0.3421
C	-2.9467	-0.9308	-0.4773
O	-2.6455	1.4299	-0.7543
H	-3.4389	-1.5394	0.2805
H	-3.3298	-1.2425	-1.4546
Li	1.4860	-0.0091	0.2571
O	1.4820	1.9123	-0.0021
O	3.2857	-0.6677	-0.0898
C	2.6167	2.6304	0.3990
H	3.0889	3.1338	-0.4536
H	2.3607	3.3856	1.1511
H	3.3149	1.9214	0.8312
C	0.4858	2.7493	-0.5416
H	0.8364	3.2303	-1.4619
H	-0.3833	2.1341	-0.7615
H	0.1991	3.5258	0.1765
C	4.4550	-0.2865	-0.7617
H	4.6695	-0.9641	-1.5967
H	4.3022	0.7165	-1.1514
H	5.3194	-0.2835	-0.0846
C	3.3565	-1.9844	0.4089
H	4.1274	-2.0678	1.1830
H	2.3748	-2.1965	0.8236
H	3.5778	-2.6927	-0.3962
C	-4.6705	0.7644	0.3072
H	-5.4245	0.1212	-0.1402
H	-4.6026	0.5193	1.3654
H	-4.9606	1.8068	0.2027

B-TS-SS

N	1.6292	-1.2874	0.8186
O	0.2806	-0.9683	0.7966
C	2.4229	-0.1631	0.4262
C	1.8558	-2.5249	0.1037
C	3.9145	-0.4203	0.6331
H	2.1090	0.6424	1.0993
H	2.8942	-2.8339	0.2111
H	1.2135	-3.2758	0.5599
H	1.5961	-2.4671	-0.9662
H	4.0727	-0.8686	1.6105

H	4.3094	-1.0892	-0.1276
H	4.4657	0.5164	0.5881
C	1.2262	1.4756	-1.0905
C	2.1668	0.3387	-1.0634
O	0.0174	1.3581	-1.2617
H	1.7378	-0.4968	-1.6176
H	3.1293	0.6232	-1.4842
Li	-0.7549	0.0150	-0.1726
O	-2.1669	-1.0495	-0.9689
O	-1.8410	1.0526	1.0690
C	-2.2927	-2.2523	-0.2562
H	-3.2051	-2.2604	0.3508
H	-2.3067	-3.1134	-0.9352
H	-1.4200	-2.2956	0.3887
C	-3.2310	-0.8110	-1.8446
H	-4.1840	-0.7403	-1.3046
H	-3.0284	0.1335	-2.3413
H	-3.3128	-1.6071	-2.5964
C	-2.7726	2.0848	0.9260
H	-2.5059	2.9506	1.5461
H	-2.7640	2.3771	-0.1199
H	-3.7821	1.7540	1.2049
C	-1.7153	0.5927	2.3891
H	-2.6574	0.1641	2.7511
H	-0.9443	-0.1721	2.3503
H	-1.4088	1.4024	3.0617
C	1.8452	2.8400	-0.8879
H	2.5182	2.8244	-0.0342
H	2.4244	3.1110	-1.7696
H	1.0692	3.5845	-0.7318

B-SS

N	1.8351	0.0345	1.2291
O	0.9529	0.8749	0.5103
C	3.0589	0.0636	0.4171
C	1.2488	-1.2857	1.3893
C	4.0779	-1.0060	0.7710
H	3.4977	1.0517	0.6140
H	1.8975	-1.8744	2.0347
H	0.2868	-1.1519	1.8837
H	1.0872	-1.8050	0.4386
H	4.2931	-0.9849	1.8369
H	3.7239	-1.9967	0.4971
H	4.9996	-0.8118	0.2270

C	1.1792	0.7127	-0.9845
C	2.5724	0.0239	-1.0334
O	0.2025	0.0083	-1.5142
H	2.4361	-1.0017	-1.3784
H	3.2490	0.5372	-1.7115
Li	-1.0923	0.0717	-0.4346
O	-2.0838	-1.5957	-0.3018
O	-2.3170	1.2977	0.3837
C	-3.0269	-2.1470	0.5765
H	-3.9409	-2.4402	0.0442
H	-2.6228	-3.0281	1.0895
H	-3.2690	-1.3860	1.3127
C	-1.6732	-2.5033	-1.2961
H	-2.5136	-2.7849	-1.9408
H	-0.9155	-1.9842	-1.8740
H	-1.2477	-3.4080	-0.8469
C	-3.6847	1.4272	0.1047
H	-3.9038	2.3932	-0.3647
H	-3.9524	0.6288	-0.5806
H	-4.2843	1.3389	1.0196
C	-1.8494	2.2945	1.2599
H	-2.3499	2.2240	2.2328
H	-0.7837	2.1279	1.3796
H	-2.0198	3.2934	0.8437
C	1.2551	2.1598	-1.5031
H	2.0560	2.7054	-1.0122
H	1.4197	2.1346	-2.5758
H	0.3040	2.6459	-1.3014

B-S1

N	1.5271	-1.5997	0.3709
O	0.1761	-1.4559	0.1014
C	2.1466	-0.3085	0.4521
C	2.1222	-2.5233	-0.5747
C	3.6090	-0.3785	0.8755
H	1.5725	0.2155	1.2252
H	3.1228	-2.7966	-0.2428
H	1.4960	-3.4131	-0.5867
H	2.1715	-2.1264	-1.6009
H	3.7039	-1.0077	1.7568
H	4.2307	-0.7900	0.0839
H	3.9742	0.6167	1.1193
C	1.2731	1.7785	-0.6202
C	1.9877	0.5228	-0.8906

O	0.0666	1.7972	-0.3807
H	1.3408	-0.0980	-1.5208
H	2.9599	0.6776	-1.3538
Li	-0.7275	0.0383	-0.0737
O	-2.1470	-0.2661	-1.3471
O	-1.7636	0.2836	1.5530
C	-2.3679	-1.6382	-1.5447
H	-3.3101	-1.9578	-1.0852
H	-2.3894	-1.8836	-2.6133
H	-1.5342	-2.1374	-1.0616
C	-3.1465	0.5416	-1.8995
H	-4.1242	0.3320	-1.4470
H	-2.8687	1.5716	-1.6963
H	-3.2229	0.3923	-2.9846
C	-2.5456	1.3355	2.0396
H	-2.2132	1.6497	3.0373
H	-2.4273	2.1623	1.3457
H	-3.6052	1.0526	2.0963
C	-1.7786	-0.8530	2.3764
H	-2.7873	-1.2770	2.4488
H	-1.1073	-1.5602	1.8967
H	-1.4161	-0.6160	3.3835
C	2.0935	3.0465	-0.5871
H	2.9184	2.9348	0.1136
H	2.5148	3.2397	-1.5724
H	1.4752	3.8895	-0.2900

B-TS-RS

N	1.5015	-1.6262	0.7748
O	0.2272	-1.0911	0.7701
C	2.5126	-0.6913	0.3938
C	1.5237	-2.8746	0.0465
C	2.5638	0.4392	1.4151
H	3.4625	-1.2420	0.4235
H	1.1999	-2.7643	-1.0024
H	2.5268	-3.3017	0.0760
H	0.8285	-3.5524	0.5386
H	1.6342	1.0001	1.3790
H	2.6674	0.0103	2.4084
H	3.4072	1.0987	1.2261
C	1.5191	1.0658	-1.2596
C	2.3997	-0.1109	-1.0886
O	0.3112	0.9949	-1.4662
H	2.0107	-0.9298	-1.6971

H	3.4140	0.1379	-1.3983
Li	-0.6636	-0.0389	-0.2594
O	-2.3276	-0.8638	-0.8197
O	-1.4186	1.3344	0.9200
C	-2.5885	-1.9683	0.0070
H	-3.4403	-1.7754	0.6691
H	-2.7938	-2.8662	-0.5878
H	-1.6859	-2.1058	0.5952
C	-3.4018	-0.5300	-1.6509
H	-4.2909	-0.2615	-1.0653
H	-3.0915	0.3244	-2.2457
H	-3.6614	-1.3620	-2.3184
C	-2.1670	2.4953	0.7064
H	-1.7647	3.3398	1.2817
H	-2.1061	2.7212	-0.3544
H	-3.2185	2.3517	0.9878
C	-1.3891	0.9276	2.2631
H	-2.3958	0.6972	2.6303
H	-0.7734	0.0322	2.2748
H	-0.9451	1.7016	2.9008
C	2.2019	2.4157	-1.2805
H	1.4698	3.2047	-1.1292
H	2.9798	2.4803	-0.5270
H	2.6636	2.5556	-2.2582

B-RS

N	1.8108	-0.3171	1.2505
O	1.1374	0.6179	0.4310
C	2.9940	-0.7030	0.4751
C	0.9485	-1.4465	1.5537
C	4.1070	0.3030	0.7550
H	3.3196	-1.6984	0.7916
H	0.6406	-1.9974	0.6582
H	1.4858	-2.1085	2.2318
H	0.0617	-1.0625	2.0575
H	3.7706	1.3029	0.4961
H	4.3603	0.2883	1.8122
H	4.9926	0.0605	0.1726
C	1.2798	0.2220	-1.0137
C	2.5447	-0.6865	-0.9908
O	0.1887	-0.3926	-1.4317
H	2.2476	-1.6785	-1.3306
H	3.3201	-0.3048	-1.6499
Li	-1.0873	0.0615	-0.4346

O	-2.3984	-1.3458	-0.1484
O	-2.0324	1.5926	0.2237
C	-3.4329	-1.6026	0.7622
H	-4.3853	-1.7710	0.2434
H	-3.2094	-2.4823	1.3776
H	-3.5238	-0.7319	1.4053
C	-2.1700	-2.4229	-1.0257
H	-3.0460	-2.6045	-1.6589
H	-1.3205	-2.1325	-1.6350
H	-1.9351	-3.3367	-0.4684
C	-3.3554	1.9499	-0.0715
H	-3.3955	2.8854	-0.6412
H	-3.7818	1.1499	-0.6690
H	-3.9451	2.0738	0.8457
C	-1.3696	2.5688	0.9906
H	-1.8565	2.6957	1.9646
H	-0.3519	2.2176	1.1287
H	-1.3572	3.5332	0.4709
C	1.5462	1.5491	-1.7470
H	0.6785	2.1910	-1.6151
H	2.4302	2.0449	-1.3573
H	1.6717	1.3366	-2.8041

B-R2

N	-1.5196	1.6540	0.7114
O	-0.1961	1.3264	0.4825
C	-2.4086	0.5604	0.4501
C	-1.8574	2.8721	0.0052
C	-2.2258	-0.4970	1.5337
H	-3.4293	0.9582	0.5183
H	-1.7914	2.7704	-1.0907
H	-2.8665	3.1834	0.2770
H	-1.1479	3.6368	0.3152
H	-1.2086	-0.8758	1.5022
H	-2.3908	-0.0360	2.5045
H	-2.9280	-1.3166	1.4052
C	-1.5625	-1.3592	-1.0537
C	-2.2355	-0.0466	-1.0123
O	-0.3452	-1.4909	-0.9537
H	-1.5911	0.6681	-1.5342
H	-3.2150	-0.0962	-1.4850
Li	0.6264	-0.0484	-0.1747
O	2.1244	0.5630	-1.2454
O	1.6326	-0.8681	1.2926

C	2.4139	1.9016	-0.9364
H	3.3454	1.9838	-0.3650
H	2.4979	2.5069	-1.8469
H	1.5793	2.2442	-0.3327
C	3.1169	-0.0540	-2.0139
H	4.0784	-0.0709	-1.4846
H	2.7892	-1.0732	-2.1971
H	3.2545	0.4620	-2.9733
C	2.3968	-2.0313	1.4236
H	2.0621	-2.6330	2.2786
H	2.2633	-2.6021	0.5093
H	3.4615	-1.7976	1.5565
C	1.6789	-0.0380	2.4239
H	2.6984	0.3181	2.6130
H	1.0270	0.7992	2.1882
H	1.3118	-0.5622	3.3141
C	-2.4459	-2.5674	-1.2718
H	-1.9072	-3.4777	-1.0217
H	-3.3553	-2.4986	-0.6815
H	-2.7303	-2.6103	-2.3232

B-TS-RR

N	-1.7588	0.0226	-1.3117
O	-0.4247	0.1357	-0.9505
C	-2.6289	0.5056	-0.2510
C	-2.0192	-1.3456	-1.7149
C	-3.0323	1.9452	-0.5773
H	-3.5355	-0.1142	-0.2346
H	-1.7071	-2.0736	-0.9517
H	-3.0778	-1.4725	-1.9444
H	-1.4373	-1.5339	-2.6165
H	-2.1373	2.5524	-0.6798
H	-3.5712	1.9678	-1.5206
H	-3.6642	2.3610	0.2045
C	-1.1973	-0.7075	1.5325
C	-1.9814	0.5052	1.1697
O	-0.0108	-0.6704	1.8409
H	-2.8044	0.6181	1.8791
H	-1.3081	1.3598	1.2326
Li	0.7822	-0.0607	0.2426
O	2.1572	-1.2620	-0.3696
O	1.7982	1.5887	0.3698
C	2.0947	-1.3866	-1.7668
H	2.9627	-0.9188	-2.2457

H	2.0441	-2.4398	-2.0668
H	1.1846	-0.8711	-2.0595
C	3.2713	-1.8947	0.1924
H	4.2072	-1.4557	-0.1775
H	3.2075	-1.7560	1.2677
H	3.2745	-2.9686	-0.0351
C	2.8481	2.0729	1.1572
H	2.6121	3.0629	1.5687
H	2.9872	1.3696	1.9733
H	3.7782	2.1480	0.5788
C	1.4943	2.4205	-0.7210
H	2.3532	2.5202	-1.3942
H	0.6736	1.9264	-1.2352
H	1.1873	3.4174	-0.3830
C	-1.9942	-1.9752	1.7545
H	-2.2793	-1.9984	2.8083
H	-2.8958	-2.0035	1.1527
H	-1.3792	-2.8488	1.5570

B-RR

N	-1.8270	0.2914	-0.9298
O	-0.6447	-0.4138	-0.5356
C	-2.8266	0.1188	0.1336
C	-2.2752	-0.2006	-2.2165
C	-3.4914	1.4594	0.4273
H	-3.6012	-0.5987	-0.1839
H	-2.5678	-1.2593	-2.2031
H	-3.1272	0.3999	-2.5331
H	-1.4650	-0.0687	-2.9324
H	-2.7423	2.1855	0.7318
H	-3.9998	1.8355	-0.4578
H	-4.2193	1.3446	1.2267
C	-0.8793	-1.2320	0.7008
C	-2.0630	-0.4478	1.3234
O	0.2381	-1.2198	1.3810
H	-2.6788	-1.0828	1.9547
H	-1.6310	0.3520	1.9262
Li	1.0798	-0.0754	0.4023
O	2.7702	-0.7464	-0.2615
O	1.3459	1.8356	0.3096
C	3.7268	-0.3236	-1.1931
H	4.7313	-0.3062	-0.7502
H	3.7426	-0.9796	-2.0715
H	3.4532	0.6803	-1.5045

C	3.0003	-2.0554	0.2011
H	3.9522	-2.1182	0.7412
H	2.1724	-2.2771	0.8685
H	3.0111	-2.7680	-0.6310
C	2.4316	2.5030	0.8920
H	2.0997	3.1823	1.6860
H	3.0828	1.7459	1.3180
H	2.9861	3.0836	0.1434
C	0.4178	2.7177	-0.2727
H	0.8818	3.2907	-1.0846
H	-0.3929	2.1119	-0.6678
H	0.0243	3.4188	0.4729
C	-1.3134	-2.6488	0.2674
H	-1.3633	-3.2722	1.1554
H	-2.2808	-2.6481	-0.2286
H	-0.5593	-3.0543	-0.4025

B-R1

N	-1.5761	-0.0195	-1.4938
O	-0.2803	0.4163	-1.2586
C	-2.5029	0.5436	-0.5525
C	-1.6363	-1.4586	-1.5311
C	-2.7764	1.9961	-0.9348
H	-3.4350	-0.0332	-0.6152
H	-1.4477	-1.9064	-0.5395
H	-2.6189	-1.7745	-1.8874
H	-0.8715	-1.8109	-2.2186
H	-1.8285	2.5240	-0.9908
H	-3.2464	2.0315	-1.9138
H	-3.4241	2.4818	-0.2089
C	-1.4607	-0.7416	1.4630
C	-2.0049	0.5350	0.9454
O	-0.2551	-0.9462	1.5915
H	-2.8639	0.8422	1.5421
H	-1.2069	1.2772	0.9938
Li	0.6788	-0.0716	0.1195
O	2.1175	-1.2139	-0.4714
O	1.7012	1.4822	0.6914
C	2.3202	-1.1119	-1.8568
H	3.2851	-0.6425	-2.0829
H	2.2821	-2.0981	-2.3345
H	1.5074	-0.4894	-2.2206
C	3.0590	-2.0261	0.1700
H	4.0739	-1.6209	0.0625

H	2.7890	-2.0528	1.2214
H	3.0442	-3.0463	-0.2343
C	2.6264	1.7429	1.7082
H	2.4212	2.7059	2.1939
H	2.5313	0.9442	2.4381
H	3.6524	1.7592	1.3192
C	1.7088	2.4504	-0.3270
H	2.6925	2.5139	-0.8047
H	0.9701	2.1168	-1.0493
H	1.4386	3.4375	0.0671
C	-2.4740	-1.7322	1.9982
H	-2.7913	-1.4012	2.9877
H	-3.3508	-1.7800	1.3587
H	-2.0280	-2.7188	2.0950

B-TS-S2

N	1.8739	-0.9739	1.3122
O	0.6454	-0.2757	1.5156
C	2.5859	-0.3241	0.1768
C	1.6060	-2.3789	1.1647
C	2.4466	-1.0083	-1.1863
H	3.6477	-0.3605	0.4596
H	2.5503	-2.9014	1.0202
H	1.1527	-2.7263	2.0928
H	0.9197	-2.6097	0.3344
H	2.8939	-1.9997	-1.1725
H	1.4037	-1.0864	-1.4787
H	2.9643	-0.4085	-1.9312
C	1.1580	1.6335	-0.7205
C	2.1293	1.1319	0.1609
O	0.2399	0.9536	-1.2442
H	1.2007	0.7748	1.0895
H	2.8627	1.8426	0.5216
Li	-0.5052	-0.1287	0.0455
O	-1.5258	-1.5314	-0.7754
O	-1.8934	0.9862	0.7809
C	-2.2374	-2.5873	-0.1873
H	-3.3184	-2.4082	-0.2319
H	-2.0157	-3.5385	-0.6878
H	-1.9232	-2.6420	0.8509
C	-1.8398	-1.3489	-2.1324
H	-2.9036	-1.1213	-2.2629
H	-1.2435	-0.5108	-2.4742
H	-1.5919	-2.2434	-2.7162
C	-2.5985	1.9244	0.0147

H	-2.3811	2.9463	0.3451
H	-2.2633	1.8020	-1.0098
H	-3.6811	1.7545	0.0765
C	-2.1406	1.0992	2.1576
H	-3.1843	0.8556	2.3923
H	-1.4744	0.3971	2.6471
H	-1.9224	2.1117	2.5144
C	1.1457	3.1400	-0.9366
H	1.2566	3.6658	0.0084
H	1.9750	3.4217	-1.5830
H	0.2145	3.4367	-1.4127

B-S2-IM1

N	1.9531	-0.9745	1.3507
O	0.6745	-0.3101	1.5015
C	2.5724	-0.6376	0.0544
C	1.7226	-2.3781	1.5744
C	2.0102	-1.4061	-1.1492
H	3.6182	-0.9439	0.1916
H	2.6608	-2.9089	1.4318
H	1.3976	-2.5047	2.6074
H	0.9551	-2.8000	0.9098
H	2.2406	-2.4682	-1.0867
H	0.9348	-1.2674	-1.2171
H	2.4597	-1.0065	-2.0536
C	1.5109	1.5833	-0.6197
C	2.5556	0.8547	-0.1622
O	0.3207	1.1394	-0.8852
H	0.9406	0.6171	1.3883
H	3.4841	1.3634	0.0456
Li	-0.6338	0.0032	-0.0138
O	-1.6844	-1.2338	-1.0454
O	-2.0306	0.9201	0.9569
C	-2.5277	-2.3240	-0.7911
H	-3.5801	-2.0546	-0.9413
H	-2.2845	-3.1713	-1.4444
H	-2.3768	-2.6143	0.2450
C	-1.7821	-0.7654	-2.3686
H	-2.7998	-0.4265	-2.5904
H	-1.0928	0.0684	-2.4463
H	-1.5008	-1.5483	-3.0823
C	-2.2123	2.2387	0.5052
H	-2.0746	2.9562	1.3217
H	-1.4551	2.3979	-0.2562

H	-3.2107	2.3747	0.0744
C	-2.9121	0.5685	1.9874
H	-3.9549	0.6121	1.6494
H	-2.6698	-0.4477	2.2836
H	-2.7943	1.2324	2.8517
C	1.7076	3.0791	-0.8360
H	2.7315	3.3815	-0.6389
H	1.4516	3.3282	-1.8645
H	1.0370	3.6382	-0.1828

B-S2

N	1.5535	-1.4892	0.9301
O	0.2239	-1.1408	0.6799
C	2.4741	-0.5799	0.2676
C	1.7646	-2.8811	0.5845
C	2.3850	-0.5673	-1.2625
H	3.4799	-0.9121	0.5604
H	2.8023	-3.1502	0.7859
H	1.1104	-3.4758	1.2210
H	1.5234	-3.1144	-0.4639
H	2.7479	-1.5107	-1.6649
H	1.3443	-0.4368	-1.5549
H	2.9812	0.2389	-1.6819
C	1.5695	1.8080	-0.0367
C	2.2509	0.8431	0.8771
O	0.3939	1.7273	-0.3459
H	1.5912	0.6824	1.7307
H	3.2051	1.2489	1.2101
Li	-0.6550	0.1704	0.0260
O	-1.6806	-0.3954	-1.5371
O	-2.1456	0.5805	1.2163
C	-1.7998	-1.7939	-1.5002
H	-2.8176	-2.0992	-1.2329
H	-1.5342	-2.2385	-2.4670
H	-1.1034	-2.1181	-0.7307
C	-2.5335	0.2084	-2.4663
H	-3.5866	0.0077	-2.2311
H	-2.3520	1.2783	-2.4176
H	-2.3283	-0.1486	-3.4843
C	-3.1933	1.5041	1.2786
H	-3.1965	2.0385	2.2375
H	-3.0418	2.2170	0.4728
H	-4.1671	1.0126	1.1506
C	-2.2081	-0.3970	2.2241

H	-3.1258	-0.9907	2.1422
H	-1.3370	-1.0271	2.0611
H	-2.1639	0.0597	3.2194
C	2.4254	2.9401	-0.5574
H	2.6478	3.6186	0.2653
H	3.3699	2.5643	-0.9417
H	1.8992	3.4884	-1.3346

B-S0

N	-1.4709	-0.6871	-1.3714
O	-0.1405	-0.2998	-1.3785
C	-2.3099	0.4280	-1.0605
C	-1.6831	-1.8350	-0.5280
C	-3.7891	0.0560	-1.0234
H	-2.1557	1.1485	-1.8716
H	-2.6127	-2.3338	-0.8038
H	-0.8511	-2.5181	-0.6785
H	-1.7202	-1.5595	0.5411
H	-4.0435	-0.5044	-1.9195
H	-4.0329	-0.5510	-0.1559
H	-4.3978	0.9574	-0.9980
C	-1.4760	0.4571	1.4502
C	-1.8783	1.2276	0.2516
O	-0.2960	0.2129	1.7123
H	-2.7153	1.8846	0.4844
H	-1.0026	1.8021	-0.0533
Li	0.7569	0.0096	0.1004
O	2.1061	-1.3576	0.3179
O	1.9160	1.5421	-0.2284
C	2.3496	-2.0308	-0.8898
H	3.3486	-1.7998	-1.2782
H	2.2589	-3.1161	-0.7617
H	1.5897	-1.6710	-1.5780
C	2.9795	-1.7275	1.3460
H	4.0202	-1.4875	1.0908
H	2.6847	-1.1671	2.2282
H	2.9099	-2.8021	1.5587
C	2.8601	2.2495	0.5238
H	2.7484	3.3327	0.3835
H	2.6867	2.0020	1.5671
H	3.8838	1.9654	0.2484
C	2.0187	1.7775	-1.6098
H	3.0040	1.4845	-1.9883
H	1.2506	1.1617	-2.0673

H	1.8468	2.8353	-1.8421
C	-2.5560	0.1645	2.4729
H	-2.7344	1.0726	3.0503
H	-3.4907	-0.1229	2.0010
H	-2.2277	-0.6141	3.1574

B-R0

N	-1.5761	-0.0189	-1.4938
O	-0.2804	0.4169	-1.2584
C	-2.5031	0.5440	-0.5526
C	-1.6364	-1.4580	-1.5313
C	-2.7765	1.9967	-0.9346
H	-3.4352	-0.0327	-0.6156
H	-1.4485	-1.9058	-0.5396
H	-2.6187	-1.7738	-1.8883
H	-0.8710	-1.8103	-2.2183
H	-1.8287	2.5247	-0.9899
H	-3.2459	2.0323	-1.9138
H	-3.4247	2.4820	-0.2089
C	-1.4613	-0.7418	1.4629
C	-2.0053	0.5350	0.9454
O	-0.2558	-0.9466	1.5913
H	-2.8644	0.8422	1.5420
H	-1.2072	1.2770	0.9940
Li	0.6786	-0.0717	0.1200
O	2.1170	-1.2141	-0.4719
O	1.7010	1.4818	0.6928
C	2.3195	-1.1108	-1.8573
H	3.2841	-0.6407	-2.0831
H	2.2817	-2.0966	-2.3359
H	1.5063	-0.4883	-2.2204
C	3.0596	-2.0254	0.1687
H	4.0739	-1.6187	0.0616
H	2.7897	-2.0536	1.2201
H	3.0464	-3.0453	-0.2366
C	2.6279	1.7404	1.7086
H	2.4261	2.7044	2.1938
H	2.5311	0.9424	2.4392
H	3.6536	1.7533	1.3187
C	1.7103	2.4495	-0.3260
H	2.6939	2.5109	-0.8041
H	0.9706	2.1172	-1.0479
H	1.4422	3.4374	0.0678
C	-2.4748	-1.7322	1.9980

H	-2.7922	-1.4011	2.9875
H	-3.3516	-1.7800	1.3585
H	-2.0289	-2.7189	2.0949

B-TS-R

N	-1.2511	-0.1106	-1.5182
O	-0.0599	0.3425	-1.7520
C	-2.2399	0.6435	-1.1545
C	-1.4312	-1.5306	-1.7273
C	-2.1008	2.1246	-1.1946
H	-3.2168	0.1978	-1.0802
H	-2.3741	-1.8644	-1.3049
H	-1.4054	-1.7335	-2.8009
H	-0.6059	-2.0498	-1.2482
H	-1.0962	2.3977	-0.8813
H	-2.2367	2.4780	-2.2198
H	-2.8396	2.6029	-0.5603
C	-1.6861	-0.6481	1.3698
C	-2.2796	0.5706	1.3873
O	-0.4624	-0.8399	1.0409
H	-3.3115	0.6876	1.6706
H	-1.6801	1.4603	1.3261
Li	0.6365	-0.0540	-0.0781
O	2.1439	-1.2199	-0.3122
O	1.4397	1.5306	0.6560
C	3.1253	-1.1750	-1.3090
H	4.0850	-0.8246	-0.9087
H	3.2730	-2.1626	-1.7641
H	2.7668	-0.4794	-2.0610
C	2.4336	-2.1204	0.7219
H	3.3472	-1.8333	1.2563
H	1.5849	-2.0806	1.3958
H	2.5536	-3.1403	0.3360
C	1.4229	1.7141	2.0450
H	0.9295	2.6554	2.3151
H	0.8578	0.8817	2.4509
H	2.4389	1.7139	2.4594
C	2.0689	2.5687	-0.0405
H	3.1329	2.6369	0.2208
H	1.9644	2.3370	-1.0958
H	1.5930	3.5347	0.1708
C	-2.5205	-1.8834	1.6947
H	-2.5144	-2.0478	2.7718
H	-3.5528	-1.7580	1.3769

H	-2.0918	-2.7623	1.2173

B-TS-S

N	1.1840	-1.3575	1.0166
O	-0.0133	-1.2911	1.4478
C	2.1502	-0.6808	1.5204
C	1.4145	-2.2629	-0.0909
C	3.5463	-0.7418	1.0187
H	1.8913	-0.0069	2.3206
H	2.3010	-2.8727	0.0741
H	0.5300	-2.8869	-0.1893
H	1.5250	-1.6658	-0.9941
H	3.9893	-1.7272	1.1775
H	3.5750	-0.5220	-0.0473
H	4.1521	-0.0047	1.5353
C	1.5379	1.3020	-0.9875
C	1.9190	2.1212	0.0045
O	0.5252	0.4905	-0.9309
H	2.7706	2.7714	-0.0887
H	1.3586	2.1678	0.9244
Li	-0.6975	-0.1006	0.1382
O	-1.9687	-1.1074	-0.9163
O	-1.8252	1.2308	0.9318
C	-3.0620	-1.8994	-0.5518
H	-4.0073	-1.3545	-0.6665
H	-3.1093	-2.8137	-1.1581
H	-2.9228	-2.1644	0.4920
C	-1.9961	-0.7018	-2.2587
H	-2.8854	-0.0993	-2.4749
H	-1.1032	-0.1023	-2.4000
H	-1.9785	-1.5668	-2.9331
C	-1.7644	2.5270	0.4004
H	-1.4564	3.2529	1.1616
H	-1.0181	2.4977	-0.3861
H	-2.7344	2.8330	-0.0120
C	-2.6794	1.1208	2.0345
H	-3.7200	1.3330	1.7550
H	-2.5960	0.0991	2.3911
H	-2.3834	1.8087	2.8357
C	2.3261	1.2851	-2.2942
H	1.7088	1.6888	-3.0965
H	3.2328	1.8784	-2.2161
H	2.5884	0.2619	-2.5668

DFT-optimized cartesian coordinates of all species involved in the platinum-catalyzed reaction

C

Pt	-1.7202	0.0144	0.2318
Cl	-2.1176	-2.0866	-0.3777
Cl	-3.9385	0.1430	0.5935
C	1.1476	3.3043	-0.3213
C	2.3577	3.3600	-0.9865
C	2.9469	2.2008	-1.4680
C	2.3377	0.9716	-1.2768
C	1.1254	0.9021	-0.6069
C	0.5255	2.0722	-0.1320
H	0.6729	4.1998	0.0488
H	2.8424	4.3124	-1.1352
H	3.8889	2.2507	-1.9914
H	2.8013	0.0685	-1.6451
C	-0.7278	1.9457	0.5323
C	-1.7440	1.9841	1.2282
H	-2.5433	2.2909	1.8535
N	0.4883	-0.3607	-0.3400
C	1.0339	-1.0254	0.8755
C	2.5378	-1.0186	0.9377
H	0.6546	-2.0505	0.8589
H	0.6280	-0.5008	1.7495
C	3.2026	0.0443	1.5393
C	4.5850	0.0798	1.5707
C	5.3170	-0.9481	1.0010
C	4.6616	-2.0135	0.4070
C	3.2785	-2.0507	0.3750
H	2.6349	0.8474	1.9897
H	5.0902	0.9102	2.0413
H	6.3962	-0.9206	1.0245
H	5.2290	-2.8201	-0.0327
H	2.7664	-2.8758	-0.0956
C	0.4258	-1.2054	-1.4977
C	-0.1999	-0.5286	-2.6902
H	-0.9167	0.2392	-2.3902
H	-0.7092	-1.2859	-3.2809
H	0.5763	-0.0767	-3.3067
O	0.8538	-2.3238	-1.4976

C-TS1

Pt	2.6613	-0.1394	-0.1531
Cl	4.8278	0.1081	-0.0946
Cl	1.9997	1.5761	-1.3871
C	0.2089	1.3113	1.8555
C	-0.7557	1.9709	2.5993
C	-2.0865	1.5949	2.5151
C	-2.4836	0.5625	1.6761
C	-1.5304	-0.0920	0.9188
C	-0.1781	0.2786	1.0188
H	1.2523	1.5916	1.9082
H	-0.4685	2.7818	3.2506
H	-2.8306	2.1109	3.1020
H	-3.5236	0.2805	1.6111
C	0.6669	-0.5436	0.1720
C	0.1560	-1.4697	-0.5215
H	0.1961	-2.2776	-1.2075
N	-1.8265	-1.1214	-0.0188
C	-2.3338	-0.6381	-1.3152
C	-3.6306	0.1057	-1.1459
H	-2.4765	-1.5191	-1.9453
H	-1.5729	0.0139	-1.7498
C	-3.6407	1.4941	-1.0989
C	-4.8286	2.1758	-0.9004
C	-6.0126	1.4755	-0.7408
C	-6.0079	0.0911	-0.7855
C	-4.8219	-0.5924	-0.9873
H	-2.7160	2.0395	-1.2206
H	-4.8290	3.2549	-0.8689
H	-6.9389	2.0078	-0.5845
H	-6.9301	-0.4576	-0.6651
H	-4.8153	-1.6721	-1.0226
C	-2.2062	-2.3960	0.4018
C	-1.8728	-2.7053	1.8354
H	-0.8560	-2.4005	2.0701
H	-1.9883	-3.7730	2.0013
H	-2.5529	-2.1698	2.4953
O	-2.7085	-3.1821	-0.3625

C-IM3

Pt	-2.7093	-0.0054	-0.2167
Cl	-2.4632	1.8347	1.1292
Cl	-3.3941	-1.7190	-1.4346

C	-0.2662	-1.9050	1.4718
C	0.7364	-2.4430	2.2617
C	2.0242	-1.9248	2.2358
C	2.3609	-0.8639	1.4031
C	1.3685	-0.3665	0.5859
C	0.0568	-0.8498	0.6327
H	-1.2797	-2.2763	1.4959
H	0.5135	-3.2675	2.9211
H	2.7830	-2.3443	2.8778
H	3.3577	-0.4531	1.4145
C	-0.7664	-0.0399	-0.2646
C	0.0605	0.8406	-0.8609
H	-0.1412	1.6125	-1.5779
N	1.4459	0.7301	-0.3606
C	2.3811	0.5015	-1.5024
C	3.7575	0.0644	-1.0899
H	2.4316	1.4420	-2.0575
H	1.9140	-0.2635	-2.1263
C	4.0926	-1.2851	-1.1189
C	5.3571	-1.6991	-0.7400
C	6.2965	-0.7690	-0.3268
C	5.9712	0.5770	-0.3024
C	4.7085	0.9947	-0.6854
H	3.3601	-2.0118	-1.4386
H	5.6087	-2.7482	-0.7681
H	7.2828	-1.0920	-0.0301
H	6.7036	1.3055	0.0107
H	4.4599	2.0452	-0.6735
C	1.6094	2.1041	0.2928
C	0.9015	2.2513	1.5731
H	-0.1902	2.1835	1.4169
H	1.1293	3.2301	1.9871
H	1.1652	1.4725	2.2831
O	2.2222	2.9231	-0.3137

C-IM3-TS1

Pt	-2.4032	-0.4580	-0.3375
Cl	-2.7833	1.8040	-0.8186
Cl	-2.4558	-2.6142	0.0297
C	-0.1206	-0.6343	2.4992
C	0.8154	-0.5551	3.5065
C	2.0654	0.0321	3.2922
C	2.4124	0.5598	2.0659
C	1.4745	0.4838	1.0443

C	0.2046	-0.1098	1.2483
H	-1.0829	-1.0998	2.6482
H	0.5902	-0.9615	4.4807
H	2.7778	0.0643	4.1027
H	3.3862	0.9951	1.9018
C	-0.4917	-0.0503	-0.0154
C	0.3626	0.5471	-0.8905
H	0.2299	0.7737	-1.9326
N	1.5293	0.9528	-0.2498
C	2.7623	1.2405	-0.9390
C	3.6966	0.0510	-0.9539
H	3.2606	2.0910	-0.4599
H	2.5151	1.5303	-1.9657
C	3.1984	-1.2333	-1.1290
C	4.0575	-2.3174	-1.1378
C	5.4197	-2.1287	-0.9783
C	5.9214	-0.8500	-0.8075
C	5.0625	0.2348	-0.7911
H	2.1366	-1.3899	-1.2512
H	3.6593	-3.3119	-1.2693
H	6.0890	-2.9758	-0.9853
H	6.9828	-0.6958	-0.6810
H	5.4570	1.2320	-0.6533
C	0.5830	3.2270	-0.0064
C	-0.5113	2.9294	0.7210
H	-1.4450	2.5594	-0.0127
H	-0.9027	3.7843	1.2651
H	-0.4221	2.0442	1.3571
O	1.3878	3.7037	-0.6791

C-IM5

Pt	-2.3211	-0.8325	0.0494
Cl	-3.3477	1.2835	-0.0248
Cl	-1.7839	-2.9430	0.1292
C	-0.2478	1.6087	1.8427
C	0.6256	2.5260	2.3831
C	1.9046	2.7144	1.8464
C	2.3401	1.9850	0.7627
C	1.4631	1.0602	0.2042
C	0.1561	0.8689	0.7277
H	-1.2257	1.4472	2.2735
H	0.3310	3.1047	3.2465
H	2.5645	3.4369	2.3027
H	3.3364	2.1149	0.3685

C	-0.4640	-0.1258	-0.0916
C	0.4683	-0.4995	-1.0196
H	0.3953	-1.2343	-1.7994
N	1.6204	0.2259	-0.8617
C	2.8332	0.0608	-1.6156
C	3.9470	-0.5196	-0.7743
H	3.1435	1.0286	-2.0267
H	2.6179	-0.6093	-2.4541
C	3.6714	-1.5060	0.1639
C	4.6890	-2.0383	0.9347
C	5.9907	-1.5948	0.7727
C	6.2715	-0.6145	-0.1633
C	5.2530	-0.0772	-0.9309
H	2.6570	-1.8533	0.2948
H	4.4640	-2.8024	1.6635
H	6.7845	-2.0110	1.3750
H	7.2846	-0.2634	-0.2936
H	5.4750	0.6909	-1.6588
C	-0.4689	2.8332	-1.5559
C	-1.2665	3.3652	-0.6633
H	-2.3622	2.2432	-0.3580
H	-1.9233	4.1613	-0.9724
H	-0.9069	3.4123	0.3560
O	0.1638	2.3025	-2.3575

C-IM5-TS

Pt	2.1022	-0.5514	-0.3085
Cl	3.6574	0.9820	-0.9117
Cl	1.1420	-2.5927	-0.4345
C	-0.7253	3.4431	-1.1689
C	-2.0294	3.4322	-0.6676
C	0.1502	2.4117	-0.9115
C	-2.4926	2.3900	0.1069
C	-1.6162	1.3462	0.3767
C	-0.2921	1.3447	-0.1269
H	1.1531	2.4116	-1.3133
H	-0.4047	4.2767	-1.7749
H	-2.6883	4.2556	-0.8973
H	-3.5043	2.3798	0.4811
C	0.3064	0.1270	0.3421
C	-0.6637	-0.5341	1.0672
H	-0.5880	-1.4728	1.5827
N	-1.7990	0.2019	1.1129
C	-3.0421	-0.2033	1.7157

C	-4.0763	-0.5474	0.6674
H	-3.4132	0.6023	2.3579
H	-2.8429	-1.0783	2.3419
C	-5.3848	-0.1033	0.7915
C	-6.3268	-0.4256	-0.1702
C	-5.9647	-1.1882	-1.2669
C	-4.6591	-1.6314	-1.3968
C	-3.7184	-1.3151	-0.4335
H	-5.6706	0.4939	1.6462
H	-7.3432	-0.0768	-0.0638
H	-6.6983	-1.4361	-2.0192
H	-4.3717	-2.2269	-2.2500
H	-2.7008	-1.6624	-0.5386
C	2.0247	0.1748	1.6213
C	2.2787	1.6277	1.8479
H	1.8121	2.2674	1.1062
H	3.3576	1.7807	1.7946
H	1.9352	1.8852	2.8493
O	2.0483	-0.6895	2.4364

C-IM10

Pt	1.70068400	-0.78432200	-0.41949500
Cl	3.40890100	0.18622600	-1.45721100
Cl	0.04867800	-2.20669600	0.25570800
C	-0.32655400	3.36423500	-1.31350100
C	-1.68703200	3.20243700	-1.07785600
C	0.61241500	2.54958500	-0.70484500
C	-2.15585200	2.21527700	-0.22762400
C	-1.21922900	1.39767200	0.38041600
C	0.16402100	1.55725600	0.15998800
H	1.66291600	2.66770700	-0.91425100
H	0.00401800	4.13866400	-1.98811500
H	-2.39309000	3.85347800	-1.56978000
H	-3.21223700	2.08617600	-0.05740200
C	0.84950600	0.52369300	0.95980800
C	-0.24794200	-0.18444100	1.61236900
H	-0.13079800	-0.82973300	2.46551900
N	-1.41113200	0.36868100	1.30035600
C	-2.70008700	-0.03683900	1.80973200
C	-3.69087700	-0.35201400	0.71354200
H	-3.09683100	0.76569700	2.44274800
H	-2.54486300	-0.92382500	2.43077500
C	-5.00614200	0.08134100	0.81756200
C	-5.92021000	-0.21459600	-0.17858400

C	-5.52393100	-0.93884800	-1.28977800
C	-4.21354700	-1.37344300	-1.39647900
C	-3.29953300	-1.08630600	-0.39836800
H	-5.31822500	0.65089600	1.68200500
H	-6.94123800	0.12478500	-0.08754500
H	-6.23585700	-1.16522200	-2.06914700
H	-3.90068900	-1.94199400	-2.25892500
H	-2.28171900	-1.43518100	-0.47828100
C	2.12098600	0.66822600	1.77602400
C	2.97129500	1.87698300	1.50876700
H	3.44721500	1.78032900	0.53473000
H	3.73942700	1.94219200	2.27472900
H	2.35893800	2.77514200	1.51034900
O	2.36562800	-0.13076400	2.65240300

C-IM3-TS2

Pt	-2.6800	-0.1084	0.2014
Cl	-3.0361	-0.3633	-1.9929
Cl	-3.0565	0.1430	2.3797
C	-0.3713	2.3573	-0.6426
C	0.6055	3.2551	-1.0305
C	1.9461	2.8881	-1.0474
C	2.3565	1.6166	-0.6679
C	1.3845	0.7344	-0.2416
C	0.0228	1.0828	-0.2497
H	-1.4213	2.6115	-0.6449
H	0.3274	4.2516	-1.3365
H	2.6898	3.6012	-1.3676
H	3.3986	1.3456	-0.7037
C	-0.7589	-0.0633	0.1733
C	0.1410	-1.0901	0.4046
H	-0.0123	-1.9722	0.9999
N	1.5222	-0.6272	0.1673
C	2.5026	-0.9773	1.2083
C	3.9068	-0.5652	0.8643
H	2.4562	-2.0664	1.3135
H	2.1737	-0.5061	2.1379
C	4.5306	0.4623	1.5614
C	5.8255	0.8348	1.2444
C	6.5038	0.1875	0.2253
C	5.8886	-0.8410	-0.4696
C	4.5973	-1.2210	-0.1495
H	4.0009	0.9718	2.3532
H	6.3039	1.6316	1.7932

H	7.5127	0.4801	-0.0231
H	6.4179	-1.3536	-1.2584
H	4.1226	-2.0328	-0.6798
C	1.2869	-1.6910	-0.9473
C	0.9090	-1.1345	-2.2819
H	0.0041	-0.5355	-2.2623
H	0.7495	-1.9824	-2.9442
H	1.7295	-0.5338	-2.6741
O	1.7307	-2.7919	-0.7351

C-IM6

Pt	-2.54864600	-0.09455500	0.04586700
Cl	-2.82681600	-1.04348400	-1.95675900
Cl	-3.16802600	0.74618100	2.00058400
C	-0.32343100	2.41552900	-1.01622900
C	0.60968500	3.38282000	-1.28522900
C	1.93530200	3.24288000	-0.84421200
C	2.35985200	2.14308100	-0.13335900
C	1.42667200	1.14334100	0.14782100
C	0.07453800	1.27894900	-0.29195400
H	-1.35045500	2.49387100	-1.34264700
H	0.33394900	4.26586600	-1.83998000
H	2.64390200	4.02570600	-1.07014400
H	3.38280100	2.05421000	0.19418900
C	-0.65674100	0.16646200	0.15513000
C	0.31506100	-0.73563400	0.84629900
H	0.01116000	-0.96352900	1.87575700
N	1.57380600	-0.01184000	0.82517600
C	2.72797900	-0.44034600	1.57871800
C	3.99602800	-0.43713400	0.76017300
H	2.52734000	-1.45718400	1.92971000
H	2.85415700	0.20651100	2.45645700
C	5.12300100	0.24073100	1.20567400
C	6.28545900	0.23949700	0.45420200
C	6.32981600	-0.43499700	-0.75362700
C	5.21094700	-1.11684900	-1.20171000
C	4.05179500	-1.12126400	-0.44725800
H	5.09214200	0.77077800	2.14731600
H	7.15608100	0.76842900	0.81209100
H	7.23486900	-0.43290600	-1.34192100
H	5.24240700	-1.65117800	-2.13925000
H	3.18663500	-1.66652400	-0.79144100
C	0.57814300	-2.10570100	0.13610300
C	0.44005000	-2.07843000	-1.34155300

H	-0.62689200	-2.04969700	-1.58469600
H	0.87943400	-2.97045800	-1.78148200
H	0.87851500	-1.17605100	-1.76137900
O	0.91739000	-3.05039700	0.79057300

C-IM6-TS1

Pt	2.2173	-0.2714	-0.2032
Cl	2.8051	1.5324	-1.3413
Cl	4.3690	-0.8626	-0.1850
C	-1.1632	3.4773	0.6333
C	-2.4147	3.0329	1.0756
C	-0.2012	2.5912	0.2053
C	-2.7425	1.6944	1.1067
C	-1.7804	0.7885	0.6695
C	-0.5079	1.2287	0.2226
H	0.7651	2.9123	-0.1508
H	-0.9560	4.5358	0.6293
H	-3.1444	3.7597	1.3994
H	-3.7145	1.3624	1.4355
C	0.2477	0.0807	-0.1866
C	-0.6208	-1.0450	0.1170
H	-0.6128	-2.0141	-0.3613
N	-1.8287	-0.5749	0.6062
C	-2.9832	-1.3827	0.9066
C	-4.1636	-1.0398	0.0255
H	-3.2584	-1.2482	1.9598
H	-2.7047	-2.4316	0.7626
C	-5.4488	-1.0360	0.5496
C	-6.5317	-0.7312	-0.2562
C	-6.3372	-0.4199	-1.5909
C	-5.0571	-0.4200	-2.1182
C	-3.9745	-0.7317	-1.3153
H	-5.6036	-1.2753	1.5923
H	-7.5274	-0.7326	0.1611
H	-7.1812	-0.1772	-2.2184
H	-4.8988	-0.1784	-3.1582
H	-2.9785	-0.7291	-1.7339
C	0.7630	-1.0237	1.1078
C	0.5687	-0.5332	2.5155
H	0.2143	-1.3668	3.1231
H	-0.1343	0.2931	2.5751
H	1.5327	-0.2137	2.9146
O	1.5224	-1.9845	0.8276

C-TS2

Pt	-2.0947	-0.1228	-0.2121
Cl	-3.1427	-1.7542	-1.2305
Cl	-3.7511	0.7826	0.9170
C	1.0784	-2.0820	2.3794
C	1.9835	-1.1023	2.7550
C	0.3220	-1.9480	1.2256
C	2.1603	0.0402	1.9844
C	1.4350	0.1575	0.8211
C	0.5004	-0.8186	0.4412
H	-0.3925	-2.7014	0.9286
H	0.9552	-2.9570	2.9988
H	2.5551	-1.2169	3.6629
H	2.8508	0.8087	2.2971
C	-0.1567	-0.4459	-0.8144
C	0.4095	0.5487	-1.4103
H	0.3912	1.1641	-2.2785
N	1.4650	1.2820	-0.0768
C	2.7855	1.7552	-0.4876
C	3.7484	0.6015	-0.6279
H	3.1772	2.4678	0.2478
H	2.6869	2.2736	-1.4470
C	4.9295	0.5840	0.1007
C	5.8088	-0.4782	-0.0156
C	5.5104	-1.5361	-0.8561
C	4.3349	-1.5232	-1.5878
C	3.4607	-0.4573	-1.4803
H	5.1685	1.4061	0.7608
H	6.7251	-0.4798	0.5551
H	6.1927	-2.3679	-0.9419
H	4.0968	-2.3439	-2.2468
H	2.5436	-0.4552	-2.0509
C	0.4119	2.2205	0.2351
C	0.6200	3.6247	-0.2409
H	-0.2866	4.1887	-0.0367
H	0.8256	3.6545	-1.3089
H	1.4528	4.0906	0.2837
O	-0.5842	1.8433	0.8071

C-IM1-TS2

Pt	0.3650	-0.8553	-0.1456
Cl	1.4782	-2.8337	-0.4338
Cl	-1.2255	-2.1909	0.8739
C	-4.0416	0.3097	0.1444

C	-4.5089	0.4953	-1.1457
C	-3.6556	0.9240	-2.1491
C	-2.3178	1.1886	-1.8889
C	-1.8461	1.0118	-0.6010
C	-2.7097	0.5715	0.4125
H	-4.6935	-0.0315	0.9330
H	-5.5460	0.3007	-1.3727
H	-4.0314	1.0619	-3.1515
H	-1.6840	1.5217	-2.6972
C	-2.0160	0.4257	1.6622
C	-1.4727	-0.5917	2.1833
H	-0.9722	-0.8827	3.0764
N	-0.5218	1.1781	-0.0979
C	0.3511	2.0665	-0.8851
C	1.7848	1.7061	-0.6314
H	0.1371	3.1091	-0.6247
H	0.1379	1.9300	-1.9500
C	2.1246	0.3531	-0.7558
C	3.4266	-0.0714	-0.5105
C	4.3836	0.8544	-0.1271
C	4.0499	2.1936	-0.0054
C	2.7539	2.6204	-0.2537
H	1.4149	-0.3260	-1.3540
H	3.6838	-1.1135	-0.6274
H	5.3937	0.5300	0.0685
H	4.8003	2.9111	0.2892
H	2.4945	3.6637	-0.1516
C	-0.6613	1.8117	1.3205
C	0.5048	1.4399	2.2160
H	1.2862	2.1859	2.0727
H	0.9041	0.4458	1.9916
H	0.1831	1.4831	3.2521
O	-1.1896	2.9046	1.3619

C-IM4

Pt	0.7002	-0.2929	0.0586
Cl	2.1051	-2.0289	-0.2882
Cl	-0.8695	-0.1086	-2.4333
C	-3.9414	0.4023	0.4498
C	-4.5179	1.6370	0.4984
C	-3.7488	2.7951	0.2643
C	-2.4051	2.7101	0.0640
C	-1.7477	1.4478	0.0997
C	-2.5692	0.2498	0.1389

H	-4.5372	-0.4877	0.5699
H	-5.5763	1.7343	0.6826
H	-4.2332	3.7605	0.2633
H	-1.8167	3.6031	-0.0801
C	-1.9847	-1.0037	-0.1213
C	-0.8315	-1.1423	-0.9858
H	-0.6238	-2.1587	-1.3248
N	-0.4497	1.3180	0.2851
C	0.4796	2.4243	0.3414
C	1.8307	1.7310	0.3602
H	0.3229	3.0205	1.2502
H	0.3916	3.0857	-0.5304
C	2.7079	1.7917	-0.7277
C	3.9315	1.1463	-0.6704
C	4.3046	0.4415	0.4660
C	3.4542	0.3776	1.5557
C	2.2075	0.9979	1.5089
H	2.4318	2.3600	-1.6051
H	4.5973	1.1905	-1.5187
H	5.2590	-0.0601	0.4984
H	3.7490	-0.1622	2.4431
H	1.5854	1.0368	2.3999
C	-2.5189	-2.2467	0.5042
C	-1.4574	-3.2298	0.9352
H	-1.8234	-3.7908	1.7916
H	-0.5157	-2.7355	1.1755
H	-1.2795	-3.9385	0.1275
O	-3.6954	-2.4596	0.6835

C-IM1

Pt	0.8266	-0.8448	-0.0113
Cl	2.5974	-2.2112	-0.1650
Cl	-0.1350	-2.4508	1.2135
C	-4.5387	0.3256	-0.6841
C	-4.5782	-0.8729	-1.3706
C	-3.4080	-1.5724	-1.6187
C	-2.1969	-1.0744	-1.1695
C	-2.1475	0.1130	-0.4584
C	-3.3255	0.8368	-0.2249
H	-5.4440	0.8824	-0.4940
H	-5.5241	-1.2604	-1.7166
H	-3.4348	-2.5050	-2.1593
H	-1.2744	-1.6202	-1.3535
C	-3.2999	2.0740	0.4647

C	-3.2888	3.1285	1.0393
H	-3.2759	4.0454	1.5491
N	-0.8777	0.6272	-0.0240
C	-0.4333	1.7536	-0.8787
C	1.0422	2.0093	-0.7422
H	-0.9990	2.6523	-0.6074
H	-0.6732	1.4869	-1.9154
C	1.9220	0.9373	-0.9105
C	3.2912	1.1153	-0.7557
C	3.7846	2.3687	-0.4364
C	2.9165	3.4381	-0.2861
C	1.5512	3.2611	-0.4345
H	1.5436	-0.0154	-1.3912
H	3.9580	0.2764	-0.8881
H	4.8463	2.5109	-0.3070
H	3.3031	4.4150	-0.0387
H	0.8773	4.0924	-0.2930
C	-0.6752	0.8453	1.3678
C	-1.5818	0.0994	2.3065
H	-1.0264	-0.1253	3.2140
H	-1.9713	-0.8204	1.8822
H	-2.4057	0.7631	2.5687
O	0.1387	1.6579	1.7410

C-IM1-TS1

Pt	0.5888	-0.9493	-0.1043
Cl	2.1251	-2.5826	0.1511
Cl	-0.9071	-2.2801	0.9002
C	-4.4000	1.1742	-0.2783
C	-4.8355	0.0368	-0.9179
C	-3.9052	-0.8798	-1.3979
C	-2.5541	-0.6656	-1.2236
C	-2.0582	0.4745	-0.5938
C	-3.0356	1.4183	-0.1038
H	-5.1035	1.9050	0.0905
H	-5.8911	-0.1393	-1.0535
H	-4.2379	-1.7633	-1.9202
H	-1.8313	-1.3761	-1.6071
C	-2.4862	2.4637	0.6247
C	-1.3778	2.6541	1.2270
H	-0.9069	3.5539	1.5689
N	-0.7126	0.6308	-0.3681
C	-0.0827	1.6561	-1.2108
C	1.3857	1.7529	-0.9131

H	-0.5709	2.6294	-1.0909
H	-0.2185	1.3542	-2.2634
C	2.1178	0.5651	-0.8606
C	3.4628	0.5767	-0.5200
C	4.0853	1.7830	-0.2463
C	3.3711	2.9670	-0.3275
C	2.0249	2.9543	-0.6528
H	1.6748	-0.3732	-1.3144
H	4.0122	-0.3517	-0.4740
H	5.1293	1.7991	0.0265
H	3.8609	3.9067	-0.1204
H	1.4670	3.8787	-0.6914
C	-0.4903	1.3624	1.6088
C	-1.2700	0.4309	2.4827
H	-1.4567	0.9484	3.4243
H	-0.6736	-0.4577	2.6853
H	-2.2198	0.1404	2.0464
O	0.7018	1.5369	1.6695

C-IM2

Pt	0.5281	-0.9705	-0.3318
Cl	1.9976	-2.3775	0.6542
Cl	-1.1284	-2.0783	0.7090
C	-4.0101	1.5798	0.0499
C	-4.7725	0.5487	-0.3887
C	-4.1641	-0.5625	-1.0196
C	-2.8244	-0.5941	-1.2491
C	-1.9715	0.4955	-0.9169
C	-2.5909	1.5544	-0.1077
H	-4.4449	2.4320	0.5497
H	-5.8434	0.5631	-0.2574
H	-4.7840	-1.3808	-1.3516
H	-2.3708	-1.4147	-1.7846
C	-1.7624	2.2840	0.6572
C	-0.7905	2.4225	1.4931
H	-0.1901	3.2979	1.6673
N	-0.7000	0.4320	-1.2560
C	0.0260	1.6313	-1.6413
C	1.4059	1.6889	-1.0426
H	-0.5354	2.5430	-1.4128
H	0.1332	1.5784	-2.7388
C	2.1435	0.5072	-0.9297
C	3.4104	0.5197	-0.3619
C	3.9489	1.7139	0.0866

C	3.2265	2.8891	-0.0331
C	1.9590	2.8771	-0.5924
H	1.7901	-0.4326	-1.4563
H	3.9647	-0.4017	-0.2679
H	4.9311	1.7259	0.5332
H	3.6456	3.8195	0.3205
H	1.3941	3.7948	-0.6766
C	-0.3235	1.1041	2.1087
C	-1.2494	0.4273	3.0552
H	-2.0461	1.0782	3.3992
H	-0.6816	0.0303	3.8942
H	-1.6679	-0.4368	2.5263
O	0.7091	0.6382	1.6932

C-IM2-TS

Pt	0.3046	-0.5308	-0.6085
Cl	1.3036	-2.3208	-1.6475
Cl	-1.3045	-1.8953	0.2666
C	-4.1391	0.2493	0.0180
C	-4.5987	1.1899	-0.8985
C	-3.7218	2.1120	-1.4348
C	-2.3839	2.1286	-1.0670
C	-1.9062	1.2214	-0.1315
C	-2.8226	0.2719	0.4066
H	-4.8083	-0.4869	0.4355
H	-5.6364	1.1921	-1.1944
H	-4.0739	2.8341	-2.1566
H	-1.7172	2.8421	-1.5319
C	-2.1021	-0.6667	1.3028
C	-1.1527	0.0524	2.0199
H	-1.5354	0.8826	2.6041
N	-0.6026	1.1093	0.3407
C	0.2571	2.2703	0.4065
C	1.6903	1.8710	0.1713
H	0.1577	2.7465	1.3919
H	-0.0266	3.0254	-0.3433
C	1.9697	0.8592	-0.7613
C	3.2801	0.4448	-0.9811
C	4.3143	1.0357	-0.2769
C	4.0442	2.0363	0.6427
C	2.7410	2.4456	0.8701
H	1.1721	0.5597	-1.5594
H	3.4794	-0.3377	-1.6985
H	5.3303	0.7130	-0.4432

H	4.8513	2.4932	1.1954
H	2.5364	3.2148	1.6008
C	0.0602	-0.5992	2.5932
C	-0.1045	-1.0530	4.0189
H	-0.2585	-0.1891	4.6623
H	0.7792	-1.5966	4.3416
H	-0.9842	-1.6883	4.0964
O	1.0822	-0.7639	1.9699

C-IM8

Pt	-0.2175	0.7912	0.1210
Cl	-0.9224	2.8825	0.6856
Cl	1.3447	0.6639	1.7991
C	3.9929	-0.0814	-0.3359
C	4.3200	0.1485	-1.6660
C	3.3933	-0.0679	-2.6712
C	2.1108	-0.5193	-2.3833
C	1.7923	-0.7573	-1.0608
C	2.7310	-0.5474	-0.0359
H	4.7053	0.0969	0.4538
H	5.3059	0.5069	-1.9196
H	3.6626	0.1201	-3.6995
H	1.3998	-0.6616	-3.1853
C	2.1026	-0.8517	1.2867
C	0.9364	-1.7643	0.8108
H	1.3382	-2.7745	0.6465
N	0.5512	-1.1635	-0.4890
C	-0.4181	-1.8906	-1.3078
C	-1.7794	-1.2403	-1.2273
H	-0.4834	-2.9316	-0.9707
H	-0.0777	-1.9012	-2.3492
C	-1.8854	0.1615	-1.2146
C	-3.1345	0.7670	-1.0967
C	-4.2738	-0.0110	-0.9951
C	-4.1725	-1.3923	-1.0144
C	-2.9330	-2.0009	-1.1224
H	-1.0048	0.8208	-1.5055
H	-3.2022	1.8450	-1.0832
H	-5.2396	0.4600	-0.8975
H	-5.0600	-2.0010	-0.9311
H	-2.8612	-3.0791	-1.1169
C	-0.1765	-1.7931	1.8182
C	0.1235	-2.6117	3.0373
H	0.1328	-3.6691	2.7802

H	-0.6261	-2.4322	3.8022
H	1.1140	-2.3460	3.4019
O	-1.1947	-1.1501	1.6739

C-TS3

Pt	2.6099	-0.4642	-0.2910
Cl	0.8306	-1.5863	-1.1586
Cl	4.5466	0.5751	0.0167
C	0.7923	2.5999	0.4317
C	0.1227	3.5907	-0.2552
C	-1.1547	3.3500	-0.7423
C	-1.7721	2.1278	-0.5379
C	-1.1152	1.1146	0.1497
C	0.1878	1.3514	0.6298
H	1.7911	2.7570	0.8112
H	0.5927	4.5480	-0.4168
H	-1.6792	4.1223	-1.2842
H	-2.7723	1.9672	-0.9097
C	0.9090	0.3176	1.2665
C	1.8353	-0.4714	1.5938
H	2.1164	-1.1308	2.3928
N	-1.7278	-0.1276	0.3717
C	-2.5698	-0.6663	-0.6773
C	-4.0514	-0.4332	-0.4892
H	-2.3683	-1.7368	-0.7700
H	-2.2457	-0.2135	-1.6191
C	-4.5543	0.3607	0.5326
C	-5.9196	0.5579	0.6587
C	-6.7949	-0.0337	-0.2343
C	-6.2998	-0.8271	-1.2567
C	-4.9375	-1.0257	-1.3822
H	-3.8828	0.8274	1.2393
H	-6.2970	1.1768	1.4589
H	-7.8584	0.1212	-0.1347
H	-6.9769	-1.2927	-1.9568
H	-4.5508	-1.6445	-2.1787
C	-1.5413	-0.7707	1.5544
C	-2.3192	-2.0370	1.7727
H	-2.2081	-2.3303	2.8130
H	-1.8930	-2.8183	1.1447
H	-3.3720	-1.9199	1.5318
O	-0.7736	-0.3472	2.4123

C-IM11

Pt	3.0831	-0.1388	-0.0559
Cl	2.4767	0.3012	-2.1879
Cl	4.1187	-0.1620	1.9312
C	0.5084	1.6574	0.4938
C	-0.0143	2.9313	0.3388
C	-1.3104	3.0998	-0.1086
C	-2.0971	1.9949	-0.4114
C	-1.5617	0.7251	-0.2967
C	-0.2490	0.5432	0.1580
H	1.5129	1.5088	0.8790
H	0.5917	3.7905	0.5784
H	-1.7277	4.0882	-0.2202
H	-3.1184	2.1498	-0.7194
C	0.2575	-0.8195	0.2944
C	1.5303	-1.2484	0.3124
H	1.6910	-2.3245	0.4253
N	-2.3313	-0.4443	-0.5860
C	-3.5900	-0.3294	-1.2910
C	-4.7955	-0.1370	-0.3948
H	-3.7320	-1.2245	-1.9025
H	-3.5038	0.5153	-1.9808
C	-4.6747	0.1898	0.9484
C	-5.8067	0.3682	1.7267
C	-7.0653	0.2237	1.1714
C	-7.1915	-0.1011	-0.1695
C	-6.0630	-0.2823	-0.9473
H	-3.7005	0.3123	1.3984
H	-5.6995	0.6228	2.7700
H	-7.9454	0.3644	1.7796
H	-8.1700	-0.2149	-0.6102
H	-6.1618	-0.5352	-1.9929
C	-1.9269	-1.6169	-0.1040
C	-2.8187	-2.8104	-0.1038
H	-2.3723	-3.5734	0.5283
H	-2.9119	-3.2179	-1.1109
H	-3.8122	-2.5600	0.2628
O	-0.7517	-1.8080	0.3796

C-IM1-TS3

Pt	1.0191	-0.9215	-0.1680
Cl	1.2111	-2.3691	1.5326
Cl	3.0271	-1.6950	-0.8516
C	-4.4267	-0.5494	-0.7035
C	-4.3177	-1.8850	-1.0378

C	-3.0965	-2.5328	-0.9356
C	-1.9830	-1.8375	-0.4975
C	-2.0827	-0.4964	-0.1674
C	-3.3117	0.1653	-0.2642
H	-5.3756	-0.0392	-0.7720
H	-5.1894	-2.4255	-1.3735
H	-3.0095	-3.5778	-1.1875
H	-1.0248	-2.3445	-0.3899
C	-3.4466	1.5303	0.0814
C	-3.5787	2.6886	0.3690
H	-3.7044	3.6948	0.6388
N	-0.8981	0.1995	0.2661
C	-0.1000	0.7464	-0.8184
C	0.5472	2.0875	-0.6120
H	-0.7526	0.7868	-1.7013
H	0.9382	-0.1002	-1.4733
C	1.9291	2.2267	-0.5350
C	2.4929	3.4808	-0.3758
C	1.6861	4.6027	-0.2940
C	0.3099	4.4697	-0.3806
C	-0.2598	3.2203	-0.5458
H	2.5693	1.3536	-0.6027
H	3.5657	3.5780	-0.3167
H	2.1272	5.5795	-0.1677
H	-0.3233	5.3421	-0.3257
H	-1.3330	3.1142	-0.6134
C	-0.8881	0.5662	1.6301
C	0.3759	1.1695	2.1770
H	0.3513	1.0424	3.2571
H	0.4199	2.2336	1.9541
H	1.2710	0.6780	1.7827
O	-1.8604	0.3417	2.3024

C-IM12

Pt	0.8355	-1.0899	-0.2762
Cl	0.4128	-2.1207	1.7537
Cl	2.5495	-2.5406	-0.4262
C	-4.4089	0.2912	-0.6751
C	-4.5564	-1.0210	-1.0805
C	-3.4738	-1.8858	-1.0520
C	-2.2415	-1.4354	-0.6098
C	-2.0868	-0.1224	-0.1999
C	-3.1726	0.7607	-0.2314
H	-5.2465	0.9719	-0.6922

H	-5.5195	-1.3721	-1.4176
H	-3.5864	-2.9115	-1.3658
H	-1.3925	-2.1169	-0.5644
C	-3.0375	2.1092	0.1751
C	-2.9330	3.2570	0.5115
H	-2.8554	4.2564	0.8219
N	-0.7954	0.3361	0.2437
C	0.0810	0.7461	-0.7799
C	0.9790	1.9306	-0.6087
H	-0.4626	0.8097	-1.7316
H	1.2340	-0.9005	-1.7300
C	2.3628	1.7999	-0.5458
C	3.1633	2.9234	-0.4342
C	2.5927	4.1842	-0.3854
C	1.2164	4.3204	-0.4593
C	0.4108	3.2021	-0.5789
H	2.8109	0.8126	-0.5919
H	4.2352	2.8115	-0.3863
H	3.2179	5.0593	-0.2957
H	0.7667	5.3013	-0.4309
H	-0.6632	3.3073	-0.6390
C	-0.7299	0.6845	1.6287
C	0.5929	1.1487	2.1600
H	0.5831	0.9835	3.2357
H	0.7128	2.2151	1.9739
H	1.4329	0.6062	1.7247
O	-1.7228	0.5887	2.2946

C-IM13

Pt	1.0692	-0.8459	0.5069
Cl	2.0810	0.9721	1.6169
Cl	2.8895	-1.0580	-0.7970
C	-3.0282	0.4058	2.1000
C	-4.2413	0.8957	1.6574
C	-4.5293	0.9199	0.3015
C	-3.6008	0.4592	-0.6156
C	-2.3759	-0.0305	-0.1846
C	-2.0856	-0.0729	1.1865
H	-2.7927	0.3797	3.1526
H	-4.9646	1.2564	2.3721
H	-5.4781	1.2991	-0.0443
H	-3.8194	0.4777	-1.6713
C	-0.8551	-0.5765	1.6913
C	0.0261	-1.0168	2.4330

H	0.5873	-1.3704	3.2638
N	-1.4454	-0.5344	-1.1400
C	-0.1348	-0.0746	-1.1868
C	0.1964	1.3629	-1.0830
H	0.3970	-0.5159	-2.0307
H	0.6394	-2.2180	0.0063
C	-0.5549	2.3402	-0.4189
C	-0.1313	3.6555	-0.3924
C	1.0408	4.0356	-1.0231
C	1.7820	3.0880	-1.7119
C	1.3659	1.7745	-1.7466
H	-1.4701	2.0907	0.0896
H	-0.7206	4.3870	0.1401
H	1.3759	5.0606	-0.9756
H	2.6945	3.3719	-2.2134
H	1.9615	1.0432	-2.2729
C	-1.9024	-1.6426	-1.8775
C	-0.9425	-2.2622	-2.8499
H	-1.4509	-3.0975	-3.3249
H	-0.0579	-2.6330	-2.3339
H	-0.6395	-1.5521	-3.6158
O	-3.0264	-2.0458	-1.7145

C-IM13-TS1

Pt	-1.4400	-0.3261	-0.5071
Cl	-3.5429	0.3565	-1.2884
Cl	-2.3759	-1.8217	1.1291
C	2.8693	0.8297	-2.3544
C	4.2444	0.7285	-2.3024
C	4.8625	0.1224	-1.2166
C	4.1178	-0.4107	-0.1792
C	2.7342	-0.3279	-0.2293
C	2.1065	0.3227	-1.3020
H	2.3736	1.3090	-3.1834
H	4.8404	1.1307	-3.1069
H	5.9390	0.0603	-1.1788
H	4.6057	-0.8907	0.6521
C	0.6944	0.4380	-1.2835
C	-0.3891	0.8851	-1.7852
H	-0.7584	1.5785	-2.5111
N	1.8500	-0.8786	0.7109
C	0.5728	-0.3020	0.7441
C	0.4094	1.0808	1.2357
H	-0.1148	-0.9749	1.2640

H	-1.5448	-1.5034	-1.4247
C	1.4613	1.9118	1.6103
C	1.2027	3.2060	2.0254
C	-0.0944	3.6911	2.0557
C	-1.1501	2.8728	1.6810
C	-0.9004	1.5761	1.2773
H	2.4760	1.5435	1.5872
H	2.0226	3.8419	2.3232
H	-0.2861	4.7038	2.3746
H	-2.1633	3.2439	1.7099
H	-1.7526	0.9084	1.0396
C	2.1519	-2.1037	1.3204
C	1.1248	-2.6535	2.2697
H	1.5563	-3.5276	2.7512
H	0.2250	-2.9663	1.7401
H	0.8526	-1.9244	3.0299
O	3.2016	-2.6550	1.1026

C-IM14

Pt	-1.8208	-0.6552	0.1941
Cl	-2.3365	-0.4253	-2.0204
Cl	-1.5227	-0.8345	2.4907
C	2.9172	-2.5517	-0.6623
C	4.2722	-2.5976	-0.9430
C	5.0661	-1.4717	-0.7731
C	4.5388	-0.2729	-0.3184
C	3.1853	-0.2245	-0.0297
C	2.3799	-1.3611	-0.2032
H	2.2919	-3.4204	-0.7917
H	4.7136	-3.5157	-1.2989
H	6.1202	-1.5259	-0.9994
H	5.1588	0.5974	-0.1876
C	1.0076	-1.0107	0.1249
C	-0.1037	-1.6995	-0.0601
H	-0.1739	-2.7216	-0.4137
N	2.4288	0.8467	0.4512
C	1.0462	0.4251	0.6470
C	0.0264	1.2494	-0.1091
H	0.8015	0.4166	1.7189
H	-2.6598	-1.8877	0.2384
C	0.2519	1.5668	-1.4481
C	-0.6702	2.3177	-2.1529
C	-1.8311	2.7643	-1.5371
C	-2.0657	2.4709	-0.2059

C	-1.1473	1.7064	0.5212
H	1.1567	1.2239	-1.9285
H	-0.4851	2.5554	-3.1888
H	-2.5479	3.3467	-2.0952
H	-2.9470	2.8549	0.2885
H	-1.2446	1.6467	1.6011
C	2.9339	2.0693	0.8151
C	1.9557	3.0029	1.4878
H	2.5038	3.8802	1.8211
H	1.4881	2.5249	2.3457
H	1.1811	3.3147	0.7911
O	4.0867	2.3760	0.6107

C-IM13-TS2

Pt	1.9071	-0.5073	-0.2285
Cl	1.8803	-2.5918	-1.1857
Cl	2.3139	1.5517	0.6437
C	-0.5268	-1.8248	1.6313
C	-1.3626	-2.1794	2.6738
C	-2.6848	-1.7636	2.6744
C	-3.1793	-0.9824	1.6415
C	-2.3487	-0.6114	0.5974
C	-1.0127	-1.0463	0.5849
H	0.5082	-2.1530	1.5968
H	-0.9848	-2.7840	3.4833
H	-3.3408	-2.0469	3.4829
H	-4.2084	-0.6586	1.6465
C	-0.1797	-0.6566	-0.5024
C	0.0003	0.0230	-1.5220
H	0.2129	0.3577	-2.5035
N	-2.8359	0.1533	-0.5042
C	-2.1107	1.2099	-0.9775
C	-1.3340	2.0981	-0.2190
H	-2.2632	1.4520	-2.0193
H	3.2374	-0.9199	0.3395
C	-1.1957	2.0395	1.1901
C	-0.2107	2.7503	1.8185
C	0.6897	3.5193	1.0736
C	0.4308	3.7563	-0.2820
C	-0.5557	3.0588	-0.9183
H	-1.8711	1.4294	1.7681
H	-0.0894	2.6751	2.8880
H	1.4879	4.0469	1.5697
H	1.0523	4.4466	-0.8308

H	-0.7244	3.1929	-1.9772
C	-3.9381	-0.3827	-1.1879
C	-4.4723	0.4363	-2.3320
H	-5.4354	0.0204	-2.6170
H	-3.8069	0.3669	-3.1911
H	-4.5943	1.4800	-2.0527
O	-4.4293	-1.4245	-0.8354

C-IM15

Pt	1.9480	-0.1621	-0.1299
Cl	1.6253	-2.1126	-1.4682
Cl	1.9747	1.7726	0.9385
C	-0.0127	-2.6750	1.1147
C	-0.7881	-3.1557	2.1512
C	-2.1001	-2.7286	2.2763
C	-2.6415	-1.8260	1.3788
C	-1.8794	-1.3343	0.3239
C	-0.5487	-1.7726	0.1982
H	1.0163	-2.9935	1.0139
H	-0.3696	-3.8535	2.8592
H	-2.7105	-3.0914	3.0888
H	-3.6604	-1.4995	1.5002
C	0.2424	-1.2059	-0.8825
C	-0.1489	-0.0821	-1.5326
H	0.3383	0.2591	-2.4383
N	-2.3886	-0.3944	-0.5921
C	-1.4650	0.5747	-1.1743
C	-1.2332	1.7871	-0.2848
H	-1.8953	0.9250	-2.1176
H	3.3550	-0.3063	0.3722
C	-1.3501	1.7199	1.0961
C	-1.1028	2.8416	1.8684
C	-0.7293	4.0321	1.2725
C	-0.6077	4.1031	-0.1045
C	-0.8631	2.9878	-0.8793
H	-1.6314	0.7963	1.5787
H	-1.1919	2.7765	2.9420
H	-0.5264	4.9012	1.8791
H	-0.3107	5.0271	-0.5770
H	-0.7667	3.0462	-1.9548
C	-3.7305	-0.3855	-0.9101
C	-4.2379	0.8216	-1.6657
H	-5.3240	0.7969	-1.6249
H	-3.9415	0.7792	-2.7127

H	-3.8777	1.7473	-1.2232
O	-4.4680	-1.2929	-0.5982

C-IM1-TS4

Pt	-1.8367	-0.1975	-0.1143
Cl	-2.5035	-2.2664	-0.7880
Cl	-3.6294	-0.3493	1.3199
C	0.3922	3.6768	-0.4227
C	1.6700	4.1612	-0.6044
C	2.7524	3.3173	-0.4064
C	2.5598	2.0023	-0.0276
C	1.2759	1.4914	0.1626
C	0.1846	2.3503	-0.0348
H	-0.4679	4.3114	-0.5693
H	1.8234	5.1873	-0.8987
H	3.7573	3.6820	-0.5524
H	3.4165	1.3617	0.0999
C	-1.1555	1.9192	0.1499
C	-2.3862	1.9499	0.2324
H	-3.3955	2.2053	0.4314
N	1.1055	0.1624	0.5746
C	2.1536	-0.3917	1.4426
C	3.3724	-0.9038	0.7153
H	1.6988	-1.2224	1.9919
H	2.4380	0.3846	2.1580
C	4.6305	-0.7195	1.2785
C	5.7619	-1.2006	0.6444
C	5.6492	-1.8661	-0.5644
C	4.3996	-2.0555	-1.1291
C	3.2661	-1.5813	-0.4925
H	4.7227	-0.1979	2.2211
H	6.7323	-1.0530	1.0945
H	6.5318	-2.2389	-1.0617
H	4.3041	-2.5805	-2.0676
H	2.2965	-1.7515	-0.9321
C	0.1303	-0.7227	0.2124
C	-0.4361	-0.3121	-1.7586
H	-1.2327	0.0289	-2.4409
H	-0.1749	-1.3236	-2.0600
H	0.4124	0.3539	-1.8936
O	0.1997	-1.8877	0.4928

C-IM16

Pt	-1.6882	-0.2356	-0.2593

Cl	-1.8961	-2.3701	-0.9032
Cl	-3.3353	-0.6387	1.4442
C	0.1928	3.7249	-0.3853
C	1.4505	4.2423	-0.6200
C	2.5619	3.4393	-0.4228
C	2.4225	2.1447	0.0452
C	1.1647	1.6151	0.3243
C	0.0374	2.4168	0.0769
H	-0.6913	4.3179	-0.5603
H	1.5620	5.2559	-0.9709
H	3.5492	3.8195	-0.6343
H	3.3040	1.5381	0.1661
C	-1.2864	1.9111	0.2059
C	-2.5153	1.8088	0.1885
H	-3.5563	1.9434	0.3373
N	1.0487	0.3399	0.8994
C	2.2139	-0.1593	1.6476
C	3.2271	-0.8369	0.7594
H	1.8337	-0.8821	2.3767
H	2.6631	0.6760	2.1901
C	4.5856	-0.6440	0.9800
C	5.5217	-1.2796	0.1834
C	5.1082	-2.1079	-0.8458
C	3.7559	-2.3053	-1.0690
C	2.8184	-1.6767	-0.2690
H	4.9120	0.0012	1.7840
H	6.5746	-1.1253	0.3665
H	5.8382	-2.6010	-1.4696
H	3.4273	-2.9557	-1.8651
H	1.7659	-1.8482	-0.4413
C	-0.0086	-0.4993	0.8941
C	-0.5732	0.0077	-2.0104
H	-1.3185	-0.0050	-2.8098
H	0.1158	-0.8150	-2.1852
H	-0.0218	0.9442	-2.0673
O	-0.0934	-1.4907	1.5524

C-IM16-TS

Pt	-1.7570	-0.2458	0.0403
Cl	-0.6805	-2.2086	-0.6463
Cl	-3.9358	-1.1137	-0.1787
C	0.3052	3.6000	-0.5385
C	1.5313	4.0555	-0.9808
C	2.6747	3.2897	-0.7926

C	2.6159	2.0589	-0.1614
C	1.3914	1.5937	0.2924
C	0.2315	2.3723	0.1185
H	-0.5923	4.1810	-0.6796
H	1.6002	5.0120	-1.4747
H	3.6265	3.6545	-1.1462
H	3.5075	1.4674	-0.0358
C	-0.9938	1.8033	0.5639
C	-2.2435	1.7000	0.7519
H	-3.1470	2.1516	1.0912
N	1.1915	0.4022	0.9809
C	2.2860	-0.2949	1.6524
C	3.2688	-0.9065	0.6867
H	1.8256	-1.0789	2.2622
H	2.7940	0.4115	2.3184
C	4.6338	-0.7719	0.9111
C	5.5441	-1.3453	0.0409
C	5.0969	-2.0496	-1.0642
C	3.7377	-2.1858	-1.2903
C	2.8236	-1.6211	-0.4181
H	4.9857	-0.2213	1.7730
H	6.6027	-1.2393	0.2263
H	5.8078	-2.4941	-1.7445
H	3.3817	-2.7394	-2.1454
H	1.7652	-1.7462	-0.5945
C	-0.0749	0.0221	1.1952
C	-1.9569	0.2348	-1.9582
H	-2.2583	-0.6484	-2.5181
H	-0.9792	0.5834	-2.2997
H	-2.7131	1.0131	-2.0606
O	-0.5322	-0.6007	2.1241

C-IM17

Pt	-2.3608	-0.3906	0.0048
Cl	-1.6229	-1.7300	-1.7030
Cl	-3.0819	0.6478	1.9102
C	1.1952	3.2819	-1.1750
C	2.4488	3.8785	-1.1807
C	3.5083	3.3198	-0.4830
C	3.3561	2.1490	0.2503
C	2.1092	1.5606	0.2615
C	1.0297	2.1178	-0.4499
H	0.3719	3.7135	-1.7213
H	2.6013	4.7890	-1.7393

H	4.4741	3.8009	-0.5082
H	4.1886	1.7168	0.7817
C	-0.1095	1.2381	-0.2411
C	-1.3688	1.2004	-0.6686
H	-1.8464	1.9266	-1.3210
N	1.6928	0.4021	0.9177
C	2.5474	-0.4740	1.6844
C	3.5811	-1.1345	0.8060
H	1.8925	-1.2290	2.1325
H	3.0277	0.0970	2.4853
C	4.9202	-1.1478	1.1689
C	5.8596	-1.7545	0.3530
C	5.4677	-2.3452	-0.8357
C	4.1325	-2.3342	-1.2034
C	3.1926	-1.7338	-0.3859
H	5.2297	-0.6878	2.0972
H	6.8991	-1.7625	0.6456
H	6.2010	-2.8152	-1.4736
H	3.8206	-2.7969	-2.1275
H	2.1507	-1.7339	-0.6726
C	0.3935	0.1535	0.6500
C	-4.1378	-0.0600	-0.9239
H	-4.7674	-0.9029	-0.6217
H	-4.0207	-0.0610	-2.0060
H	-4.5891	0.8712	-0.5874
O	-0.2637	-0.7962	1.0695

C-IM17-TS

Pt	-2.2693	-0.3177	-0.0901
Cl	-3.1540	-1.4849	1.5853
Cl	-4.3050	0.5166	-0.5293
C	1.3467	3.4857	-0.2366
C	2.5937	4.0346	0.0250
C	3.6193	3.2633	0.5506
C	3.4373	1.9160	0.8379
C	2.1967	1.3720	0.5832
C	1.1486	2.1461	0.0433
H	0.5497	4.0861	-0.6457
H	2.7691	5.0786	-0.1844
H	4.5804	3.7155	0.7420
H	4.2428	1.3214	1.2381
C	0.0155	1.2543	-0.1083
C	-1.2450	1.3246	-0.5888
H	-1.6718	2.2439	-0.9714

N	1.7550	0.0606	0.7855
C	2.5517	-1.0194	1.3184
C	3.6966	-1.3667	0.3999
H	1.8774	-1.8759	1.4268
H	2.9263	-0.7386	2.3081
C	4.9843	-1.5186	0.8942
C	6.0280	-1.8351	0.0418
C	5.7937	-1.9945	-1.3129
C	4.5108	-1.8440	-1.8124
C	3.4671	-1.5341	-0.9599
H	5.1712	-1.3945	1.9516
H	7.0259	-1.9539	0.4368
H	6.6083	-2.2374	-1.9782
H	4.3225	-1.9714	-2.8679
H	2.4648	-1.4247	-1.3491
C	0.4759	-0.0488	0.3819
C	-1.7054	-0.1091	-2.1214
H	-2.1578	-1.0792	-2.3862
H	-0.6528	-0.1444	-2.3866
H	-2.2125	0.6588	-2.6964
O	-0.2260	-1.0777	0.4157

C-IM18

Pt	2.1094	-0.4763	0.1287
Cl	1.4239	-1.8046	-1.4915
Cl	4.0562	-0.1773	-0.8669
C	-1.1297	3.6166	-0.2554
C	-2.1911	3.9164	-1.0974
C	-2.9678	2.9104	-1.6516
C	-2.7146	1.5706	-1.3861
C	-1.6606	1.2764	-0.5466
C	-0.8673	2.2882	0.0224
H	-0.5270	4.4026	0.1722
H	-2.4144	4.9474	-1.3248
H	-3.7870	3.1707	-2.3044
H	-3.3247	0.7940	-1.8176
C	0.1561	1.6344	0.8240
C	1.1923	2.1779	1.4867
H	1.2950	3.2544	1.4912
N	-1.2049	0.0346	-0.1145
C	-1.7998	-1.2326	-0.4686
C	-3.2271	-1.3295	0.0040
H	-1.1861	-2.0059	0.0069
H	-1.7394	-1.3665	-1.5530

C	-4.2113	-1.8514	-0.8242
C	-5.5208	-1.9411	-0.3855
C	-5.8598	-1.5022	0.8828
C	-4.8828	-0.9812	1.7143
C	-3.5729	-0.8983	1.2785
H	-3.9493	-2.1934	-1.8154
H	-6.2782	-2.3510	-1.0370
H	-6.8822	-1.5678	1.2235
H	-5.1412	-0.6416	2.7064
H	-2.8103	-0.4994	1.9323
C	-0.1374	0.1725	0.7101
C	2.2298	1.3925	2.2154
H	1.7900	0.8636	3.0637
H	3.0356	2.0317	2.5668
H	2.6926	0.6090	1.5557
O	0.3694	-0.7709	1.3565

C-IM3-TS3

Pt	-2.6912	-0.1002	-0.2486
Cl	-2.3778	-1.5074	-1.9449
Cl	-3.6486	1.1356	1.3213
C	-0.1787	-1.6366	1.6799
C	0.8758	-2.1829	2.3908
C	2.1352	-1.5998	2.3550
C	2.3860	-0.4551	1.6094
C	1.3295	0.1022	0.9122
C	0.0542	-0.4908	0.9347
H	-1.1648	-2.0778	1.6873
H	0.7224	-3.0745	2.9790
H	2.9449	-2.0443	2.9130
H	3.3779	-0.0352	1.5969
C	-0.8180	0.3092	0.0906
C	-0.0534	1.3188	-0.4023
H	-0.3131	2.1320	-1.0539
N	1.2902	1.1973	0.0129
C	2.2180	0.4120	-1.6380
C	3.4501	-0.1427	-1.2228
H	2.2209	1.3459	-2.1783
H	1.4037	-0.2600	-1.8644
C	3.5333	-1.4961	-0.8364
C	4.7293	-2.0288	-0.4065
C	5.8624	-1.2304	-0.3254
C	5.7974	0.1102	-0.6902

C	4.6114	0.6520	-1.1313
H	2.6506	-2.1135	-0.9046
H	4.7844	-3.0699	-0.1268
H	6.7962	-1.6498	0.0168
H	6.6826	0.7260	-0.6295
H	4.5578	1.6911	-1.4227
C	1.9995	2.4369	0.1589
C	2.7866	2.6082	1.4181
H	2.9383	3.6723	1.5847
H	3.7640	2.1420	1.2987
H	2.2817	2.1615	2.2698
O	1.9217	3.2614	-0.7060

C-IM19

Pt	-2.1892	-0.0791	-0.1239
Cl	-2.2433	-2.2807	-0.3629
Cl	-3.1932	1.8842	0.0566
C	0.0897	0.1521	2.5010
C	1.0398	0.3072	3.4828
C	2.2969	0.8191	3.1632
C	2.6331	1.2068	1.8767
C	1.6839	1.0751	0.8720
C	0.4114	0.5230	1.1910
H	-0.8883	-0.2625	2.7021
H	0.8253	0.0233	4.5011
H	3.0384	0.9150	3.9421
H	3.6244	1.5784	1.6904
C	-0.3599	0.4218	0.0110
C	0.5326	0.8077	-1.1187
H	0.0924	1.5500	-1.7926
N	1.7235	1.3681	-0.4745
C	0.9316	-0.4421	-1.9637
C	1.9075	-1.2809	-1.1907
H	1.3838	-0.0889	-2.8900
H	0.0187	-0.9974	-2.1861
C	1.4683	-2.1350	-0.1866
C	2.3777	-2.8386	0.5820
C	3.7368	-2.6952	0.3584
C	4.1823	-1.8553	-0.6477
C	3.2726	-1.1509	-1.4167
H	0.4084	-2.2490	-0.0128
H	2.0239	-3.4995	1.3589
H	4.4467	-3.2406	0.9623
H	5.2411	-1.7483	-0.8345

H	3.6233	-0.4963	-2.2020
C	2.6136	2.0650	-1.2648
C	3.8107	2.6815	-0.5981
H	4.3148	3.3097	-1.3285
H	4.4976	1.9024	-0.2746
H	3.5118	3.2858	0.2542
O	2.3975	2.1733	-2.4503

C-TS4

Pt	-2.2338	0.3233	-0.2314
Cl	-3.4115	-1.5599	-0.6184
Cl	-1.6728	2.4135	0.4890
C	2.5481	-1.7330	-2.0663
C	3.7655	-2.2607	-1.6590
C	4.1256	-2.3335	-0.3203
C	3.2858	-1.8691	0.6899
C	2.0963	-1.3282	0.2759
C	1.7192	-1.2652	-1.0603
H	2.2651	-1.6933	-3.1052
H	4.4492	-2.6368	-2.4051
H	5.0759	-2.7694	-0.0532
H	3.5609	-1.9491	1.7292
C	0.4251	-0.6763	-1.0115
C	-0.6847	-0.1622	-1.3660
H	-0.9869	0.0560	-2.3930
N	0.9636	-0.7645	0.9822
C	1.1294	0.5886	1.5213
C	2.2152	1.3267	0.7841
H	1.3830	0.5244	2.5856
H	0.1826	1.1295	1.4023
C	1.9091	2.0671	-0.3504
C	2.9122	2.7086	-1.0548
C	4.2277	2.6201	-0.6320
C	4.5362	1.9012	0.5099
C	3.5342	1.2592	1.2150
H	0.8809	2.1512	-0.6664
H	2.6648	3.2845	-1.9336
H	5.0097	3.1200	-1.1834
H	5.5579	1.8428	0.8545
H	3.7803	0.7088	2.1119
C	0.1364	-1.7109	1.6251
C	-1.0220	-1.1428	2.3757
H	-1.5888	-1.9589	2.8154
H	-1.6757	-0.5971	1.6591

H	-0.7136	-0.4421	3.1465
O	0.3652	-2.8858	1.4937

C-IM20

Pt	-2.3049	0.3239	-0.2326
Cl	-3.5714	-1.4579	-0.7382
Cl	-1.6070	2.3202	0.6231
C	2.5380	-1.6442	-2.1256
C	3.7847	-2.1656	-1.7830
C	4.2124	-2.2907	-0.4687
C	3.4232	-1.8897	0.6158
C	2.2203	-1.3633	0.2530
C	1.7612	-1.2461	-1.0602
H	2.2177	-1.5682	-3.1510
H	4.4439	-2.4953	-2.5719
H	5.1842	-2.7154	-0.2717
H	3.7564	-2.0060	1.6338
C	0.4945	-0.6904	-0.6069
C	-0.6537	-0.2512	-1.1067
H	-0.7121	-0.2672	-2.1997
N	0.9962	-0.7854	0.8409
C	1.1962	0.5335	1.5005
C	2.2482	1.3220	0.7684
H	1.5093	0.3474	2.5309
H	0.2461	1.0782	1.4677
C	1.8967	2.0821	-0.3404
C	2.8683	2.7680	-1.0470
C	4.1939	2.7046	-0.6515
C	4.5460	1.9649	0.4644
C	3.5770	1.2758	1.1710
H	0.8601	2.1458	-0.6328
H	2.5880	3.3581	-1.9059
H	4.9509	3.2400	-1.2046
H	5.5757	1.9253	0.7863
H	3.8562	0.7065	2.0462
C	0.2236	-1.7853	1.6500
C	-1.0050	-1.2627	2.2632
H	-1.5180	-2.0690	2.7805
H	-1.6682	-0.8566	1.4570
H	-0.8129	-0.4401	2.9467
O	0.6517	-2.8976	1.6902

C-IM6-TS2

Pt	-1.9264	-0.4757	0.0412

Cl	-3.2106	1.0509	1.0027
Cl	-3.5877	-1.9578	0.1314
C	0.1824	4.3799	0.2184
C	1.4970	4.4216	-0.2515
C	-0.5065	3.1900	0.2870
C	2.1580	3.2840	-0.6693
C	1.4739	2.0747	-0.6098
C	0.1451	2.0247	-0.1187
H	-1.5226	3.1226	0.6423
H	-0.2935	5.2963	0.5299
H	2.0113	5.3700	-0.2947
H	3.1710	3.3487	-1.0368
C	-0.3231	0.6712	-0.1689
C	0.8620	-0.0756	-0.6961
H	0.5650	-0.0905	0.5198
N	1.8873	0.8180	-0.9927
C	3.2645	0.4374	-1.2105
C	3.7947	-0.4154	-0.0811
H	3.8545	1.3546	-1.2831
H	3.3606	-0.0931	-2.1621
C	3.4774	-0.1066	1.2362
C	3.9455	-0.8948	2.2723
C	4.7414	-1.9953	2.0026
C	5.0742	-2.2997	0.6938
C	4.6003	-1.5153	-0.3436
H	2.8716	0.7622	1.4570
H	3.6896	-0.6466	3.2910
H	5.1047	-2.6110	2.8107
H	5.7007	-3.1517	0.4778
H	4.8682	-1.7530	-1.3638
C	0.5544	-1.3968	-1.2208
C	1.4914	-2.1551	-2.1078
H	2.4169	-2.3746	-1.5777
H	1.7228	-1.5726	-2.9979
H	1.0154	-3.0857	-2.4057
O	-0.5645	-1.8570	-0.9434

C-IM9

Pt	-2.1857	-0.4603	0.1863
Cl	-3.5838	1.2618	0.3516
Cl	-3.8215	-1.6824	-0.6299
C	0.4891	4.2990	0.0639
C	1.7198	4.1401	-0.5875
C	-0.2641	3.2128	0.4356
C	2.2270	2.8968	-0.8908

C	1.4720	1.7833	-0.5273
C	0.2289	1.9386	0.1398
H	-1.2207	3.3175	0.9226
H	0.1345	5.2964	0.2725
H	2.2852	5.0191	-0.8580
H	3.1817	2.7982	-1.3846
C	-0.2729	0.6312	0.3637
C	0.6875	-0.2658	-0.1438
H	-0.9826	0.3851	1.2051
N	1.7315	0.4487	-0.6884
C	2.8734	-0.0799	-1.3869
C	4.0014	-0.4917	-0.4657
H	3.2358	0.6981	-2.0676
H	2.5629	-0.9290	-2.0010
C	4.1830	0.1240	0.7653
C	5.2302	-0.2554	1.5875
C	6.1079	-1.2469	1.1852
C	5.9361	-1.8596	-0.0449
C	4.8870	-1.4855	-0.8649
H	3.5061	0.9025	1.0861
H	5.3600	0.2283	2.5438
H	6.9240	-1.5412	1.8273
H	6.6178	-2.6326	-0.3656
H	4.7536	-1.9665	-1.8233
C	0.4281	-1.6783	-0.1022
C	1.5005	-2.7181	-0.2323
H	2.4342	-2.3894	0.2162
H	1.6724	-2.9414	-1.2849
H	1.1553	-3.6331	0.2439
O	-0.7471	-2.0457	0.0901
